# Trastuzumab deruxtecan in metastatic breast cancer with variable HER2 expression: the phase 2 DAISY trial

**DOI:** 10.1038/s41591-023-02478-2

**Published:** 2023-07-24

**Authors:** Fernanda Mosele, Elise Deluche, Amelie Lusque, Loïc Le Bescond, Thomas Filleron, Yoann Pradat, Agnes Ducoulombier, Barbara Pistilli, Thomas Bachelot, Frederic Viret, Christelle Levy, Nicolas Signolle, Alexia Alfaro, Diep T. N. Tran, Ingrid Judith Garberis, Hugues Talbot, Stergios Christodoulidis, Maria Vakalopoulou, Nathalie Droin, Aurelie Stourm, Maki Kobayashi, Tomoya Kakegawa, Ludovic Lacroix, Patrick Saulnier, Bastien Job, Marc Deloger, Marta Jimenez, Celine Mahier, Vianney Baris, Pierre Laplante, Patricia Kannouche, Virginie Marty, Magali Lacroix-Triki, Veronique Diéras, Fabrice André

**Affiliations:** 1 INSERM U981, Gustave Roussy, Villejuif, France; 2 Department of Medical Oncology, Gustave Roussy, Villejuif, France; 3 Department of Medical Oncology, CHU Dupuytren, Limoges, France; 4 Department of Biostatistics, Institut Claudius-Regaud, IUCT Oncopole, Toulouse, France; 5 CVN Lab, CentraleSupélec,Université Paris-Saclay, Gif-Sur-Yvette, France; 6 OPIS, Inria, CentraleSupélec, Université Paris-Saclay, Gif-Sur-Yvette, France; 7 MICS Lab, CentraleSupélec, Université Paris-Saclay, Gif-Sur-Yvette, France; 8 Department of Medical Oncology, Centre Antoine Lacassagne, Nice, France; 9 Department of Medical Oncology, Centre Léon Bérard, Lyon, France; 10 Department of Medical Oncology, Centre Paoli Calmettes, Marseille, France; 11 Department of Medical Oncology, Centre François Baclesse, Caen, France; 12 AMMICa Platform, INSERM US23, CNRS UAR 3655, AMMICa, Villejuif, France; 13 Imaging and Cytometry Platform, Gustave Roussy, UAR 23/3655, Université Paris-Saclay, Villejuif, France; 14 Department of Medical Biology and Pathology, Gustave Roussy, Villejuif, France; 15 Translational Research Department, Daiichi Sankyo RD Novare, Tokyo, Japan; 16 R&D Department, Unicancer, Paris, France; 17 UMR9019, CNRS, Gustave Roussy, Université Paris-Saclay, Villejuif, France; 18 Department of Medical Oncology, Centre Eugène Marquis, Rennes, France; 19 Faculty of Medicine, Université Paris-Saclay, Kremlin Bicêtre, France

**Keywords:** Breast cancer, Targeted therapies

## Abstract

The mechanisms of action of and resistance to trastuzumab deruxtecan (T-DXd), an anti-HER2–drug conjugate for breast cancer treatment, remain unclear. The phase 2 DAISY trial evaluated the efficacy of T-DXd in patients with HER2-overexpressing (*n* = 72, cohort 1), HER2-low (*n* = 74, cohort 2) and HER2 non-expressing (*n* = 40, cohort 3) metastatic breast cancer. In the full analysis set population (*n* = 177), the confirmed objective response rate (primary endpoint) was 70.6% (95% confidence interval (CI) 58.3–81) in cohort 1, 37.5% (95% CI 26.4–49.7) in cohort 2 and 29.7% (95% CI 15.9–47) in cohort 3. The primary endpoint was met in cohorts 1 and 2. Secondary endpoints included safety. No new safety signals were observed. During treatment, HER2-expressing tumors (*n* = 4) presented strong T-DXd staining. Conversely, HER2 immunohistochemistry 0 samples (*n* = 3) presented no or very few T-DXd staining (Pearson correlation coefficient *r* = 0.75, *P* = 0.053). Among patients with HER2 immunohistochemistry 0 metastatic breast cancer, 5 of 14 (35.7%, 95% CI 12.8–64.9) with *ERBB2* expression below the median presented a confirmed objective response as compared to 3 of 10 (30%, 95% CI 6.7–65.2) with *ERBB2* expression above the median. Although HER2 expression is a determinant of T-DXd efficacy, our study suggests that additional mechanisms may also be involved. (ClinicalTrials.gov identifier NCT04132960.)

## Abstract (web summary)

Trastuzumab deruxtecan, an anti-HER2–drug conjugate, exhibits the highest objective response rate in patients with HER2-overexpressing metastatic breast cancer, but clinical activity is also observed in patients with HER2-low or non-expressing tumors, potentially pointing to additional determinants of drug efficacy.

## Main

Breast cancer is the fifth leading cause of cancer mortality^1^. Despite advances in precision medicine and improvements in treatment, the 5-year survival rate of patients with metastases is only 30%^2–4^. Breast cancer includes three main subtypes: hormone receptor-positive; HER2-overexpressing; and triple-negative breast cancer (TNBC)^5^. The standard first-line treatment for patients with HER2-overexpressing metastatic breast cancer (mBC) is anti-HER2 in combination with taxanes^6^. Antibody–drug conjugates (ADCs) are drugs that deliver a cytotoxic payload to cells that express a specific target protein. Trastuzumab deruxtecan (T-DXd, DS-8201a) is a third-generation ADC composed of a humanized monoclonal anti‐HER2 (trastuzumab), a cleavable tetra peptide linker and a topoisomerase 1 (TOP1) inhibitor (DXd) as the cytotoxic payload. T-DXd is characterized by a high drug-to-antibody ratio (DAR) of 8:1 (ref. ^7^). Trastuzumab emtansine (T-DM1) is a second-generation ADC that consists of trastuzumab conjugated by a non-cleavable linker to the cytotoxic payload emtansine (DM1), a microtubule inhibitory agent. The DAR is 3.5:1 (ref. ^8^). The standard second-line treatment for HER2-overexpressing mBC was T-DM1 until the approval of T-DXd in 2022 (refs. ^9,10^). The approval was based on DB-03, a phase 3 clinical trial, in which T-DXd improved progression-free survival (PFS) as compared to T-DM1 in patients with HER2-overexpressing mBC (hazard ratio (HR): 0.28, *P* < 0.001)^10^. In addition, in DB-01, a phase 2 single-arm study, T-DXd demonstrated high anti-tumor activity in patients with HER2-overexpressing mBC who had previously received T-DM1 (ref. ^11^). These results were confirmed in the phase 3 randomized trial DB-02 (ref. ^12^). Patients with HER2-low mBC are treated according to expression of hormone receptors^13^. In this group of patients, T-DXd was recently shown to be superior to systemic chemotherapy in the second line of therapy and beyond, improving PFS (HR: 0.50, *P* < 0.001) and overall survival (OS; HR: 0.64, *P* = 0.001) in patients with HER2-low mBC as compared to chemotherapy^14^.

Although T-DXd provides some clinical benefit in patients with HER2-overexpressing and HER2-low mBC, most of them will ultimately experience disease progression and die. Although the overall structure of T-DXd is well defined, several questions remain regarding its mechanisms of action and resistance. These include the impact of HER2 expression and its spatial distribution on drug efficacy; the distribution of T-DXd in the tumor; the potential impact on the tumor microenvironment; and the molecular mechanisms of resistance. Understanding these mechanisms of action and resistance could lead to improved treatment selection for patients and the potential development of more effective combinatorial treatment strategies. To address these questions, we designed DAISY, a phase 2 trial that evaluated T-DXd efficacy in patients with mBC according to HER2 expression levels and explored treatment response and resistance through biomarker analyses of tumor samples at different timepoints.

## Results

### Study design

Patients with mBC were eligible if they had received at least one line of chemotherapy in the metastatic setting and had at least one non-bone metastatic site easily accessible to biopsy. Patients with HER2-overexpressing mBC had to be pretreated with taxanes and to be resistant to trastuzumab and TDM-1. Patients with HER2-low or HER2 immunohistochemistry (IHC) 0 tumor had to be pretreated with anthracyclines and taxanes. Patients with tumors expressing hormone receptors (estrogen and/or progesterone) had to be resistant to endocrine therapy and CDK4/6 inhibitors. Tumor biopsy was mandatory at baseline and at resistance and was optional during treatment. Additional details about patient selection and the trial design are provided in the Methods section.

**Fig. 1:**
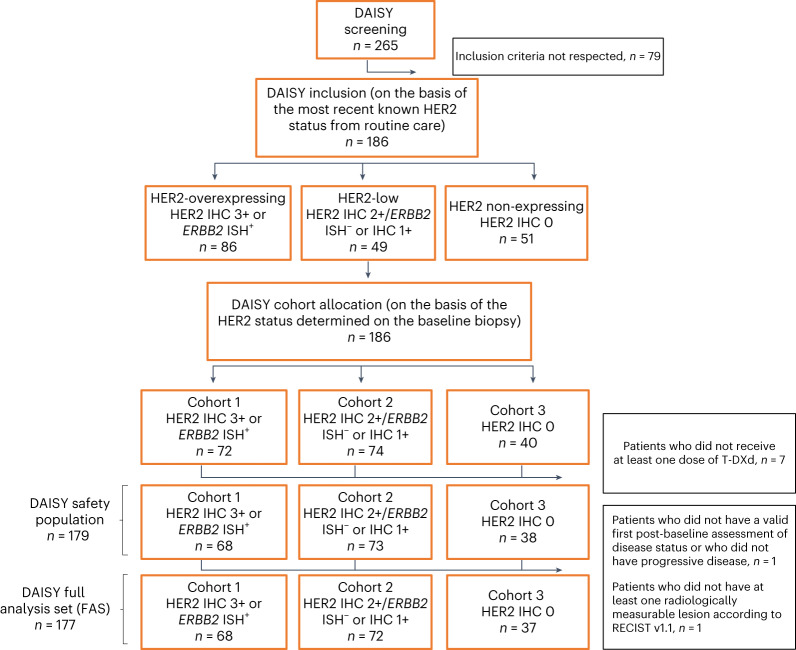
CONSORT diagram. IHC^+^, IHC-positive; IHC^−^, IHC-negative.

**Extended Data Fig. 1:**
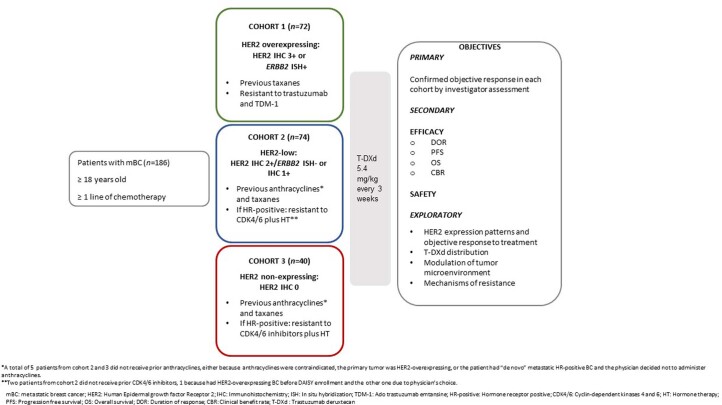
Study Design.

**Extended Data Fig. 2:**
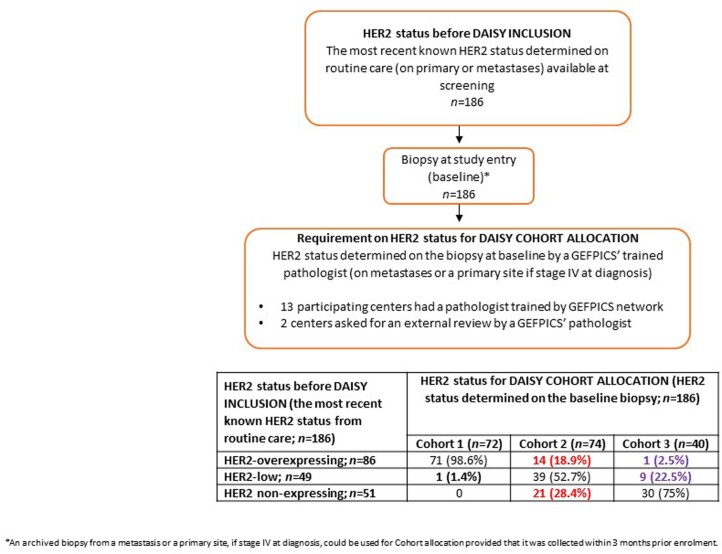
Determination of HER2 status by standard immunohistochemistry before DAISY inclusion and for cohort allocation.

In total, 186 patients were enrolled into the DAISY trial between 4 November 2019 and 3 March 2021 (Extended Data Fig. 1 and Fig. 1). Patients were assigned to a specific cohort according to HER2 status defined based on the baseline biopsy performed at study entry (Methods). Seventy-two patients with HER2-overexpressing mBC defined as IHC 3+ or *ERBB2* in situ hybridization (ISH)-positive were assigned to cohort 1; 74 patients with HER2-low mBC defined as IHC 2+/*ERBB2* ISH-negative or IHC 1+ were assigned to cohort 2; and 40 patients with non-expressing mBC defined as HER2 IHC 0 were assigned to cohort 3. Some patients presented a change in the HER2 status of the biopsy at baseline as compared to routine care and were, therefore, reassigned to different cohorts (Extended Data Fig. 2). Of the 86 patients with HER2-overexpressing mBC, 14 were enrolled after baseline biopsy into cohort 2 (HER2-low, *n* = 74) and one into cohort 3 (HER2 IHC 0, *n* = 40). Of the patients with HER2-low tumors (*n* = 49), one was enrolled into cohort 1 (HER2-overexpressing, *n* = 72) and nine into cohort 3 (HER2 IHC 0, *n* = 40). Twenty-one of the fifty-one patients with HER2-non expressing mBC were enrolled into cohort 2 (HER2-low, *n* = 74). Patients received T-DXd 5.4 mg kg^−1^ every 3 weeks until progressive disease or unacceptable toxicity. Baseline patient characteristics in the safety population (*n* = 179) are reported in Table 1. A total of 44 patients (64.7%) in cohort 1, 58 (79.5%) in cohort 2, and 26 (68.4%) in cohort 3 had hormone receptor-positive primary breast cancer (*P* = 0.13 among the three cohorts). Most patients (53.1%) were heavily pretreated with ≥5 lines of previous therapies in the metastatic setting. A total of 53 patients (72.6%) in cohort 2 and 27 (71.1%) in cohort 3 presented liver metastases at inclusion as compared to 23 (33.8%) in cohort 1 (*P* < 0.0001). There were 21 patients (30.9%) in cohort 1, 33 (45.2%) in cohort 2 and 23 (60.5%) in cohort 3 that presented an Eastern Cooperative Oncology Group (ECOG) performance status of 0 (*P* = 0.011). Prior therapy exposures were consistent with the molecular profile of breast cancer. Patient characteristics were balanced among the cohorts, except for site of metastases and performance status.

**Table 1:** Patient characteristics in the safety population

	Overall population *n* = 179	Cohort 1 *n* = 68	Cohort 2 *n* = 73	Cohort 3 *n* = 38	*P* value
Age at inclusion (years)					0.68
Median (range)	55 (24–82)	56 (30–81)	55 (24–82)	54 (36–74)	
Sex					NA
Male	1 (0.6%)	0	1 (1.4%)	0	
Female	178 (99.4%)	68 (100%)	72 (98.6%)	38 (100%)	
ECOG performance status at inclusion					0.011
ECOG 0	77 (43.0%)	21 (30.9%)	33 (45.2%)	23 (60.5%)	
ECOG 1	102 (57.0%)	47 (69.1%)	40 (54.8%)	15 (39.5%)	
Hormone receptor status of primary tumor					0.13
Hormone receptor-negative	51 (28.5%)	24 (35.3%)	15 (20.5%)	12 (31.6%)	
Hormone receptor-positive	128 (71.5%)	44 (64.7%)	58 (79.5%)	26 (68.4%)	
Most recent known HER2 status from routine care before DAISY inclusion (on primary or metastases)					NA
IHC 0	49 (27.4%)	0	21 (28.8%)	28 (73.7%)	
IHC 1+	25 (14%)	1 (1.5%)	19 (26%)	5 (13.2%)	
IHC 2+/*ERBB2* ISH-negative	23 (12.8%)	0	19 (26%)	4 (10.5%)	
IHC 2+/*ERBB2* ISH-positive	21 (11.7%)	11 (16.2%)	9 (12.3%)	1 (2.6%)	
IHC 3+	60 (33.5%)	56 (82.3%)	4 (5.5%)	0	
IHC 1+/*ERBB2* ISH-positive	1 (0.6%)	0	1 (1.4%)	0	
HER2 status determined on the baseline biopsy for DAISY cohort allocation (on primary or metastases)					NA
IHC 0	38 (21.2%)	0	0	38 (100%)	
IHC 1+	41 (22.9%)	0	41 (56.2%)	0	
IHC 2+/*ERBB2* ISH-negative	32 (17.9%)	0	32 (43.8%)	0	
IHC 2+/*ERBB2* ISH-positive	17 (9.5%)	17 (25%)	0	0	
IHC 3+	50 (27.9%)	50 (73.5%)	0	0	
IHC 1+/*ERBB2* ISH-positive	1 (0.6%)	1 (1.5%)	0	0	
Interval from initial diagnosis to metastatic disease					0.17
0–3 months	49 (27.4%)	24 (35.3%)	17 (23.3%)	8 (21.1%)	
>3 months	130 (72.6%)	44 (64.7%)	56 (76.7%)	30 (78.9%)	
Interval from metastatic disease to inclusion					0.62
0–24 months	45 (25.1%)	13 (19.1%)	20 (27.4%)	12 (31.6%)	
24–60 months	74 (41.3%)	29 (42.6%)	30 (41.1%)	15 (39.5%)	
>60 months	60 (33.5%)	26 (38.2%)	23 (31.5%)	11 (28.9%)	
Number of metastatic sites at inclusion					0.96
<3	63 (35.2%)	24 (35.3%)	25 (34.2%)	14 (36.8%)	
≥3	116 (64.8%)	44 (64.7%)	48 (65.8%)	24 (63.2%)	
Sites of metastasis at inclusion					
Liver					<0.001
No	76 (42.5%)	45 (66.2%)	20 (27.4%)	11 (28.9%)	
Yes	103 (57.5%)	23 (33.8%)	53 (72.6%)	27 (71.1%)	
Lung					0.56
No	103 (57.5%)	36 (52.9%)	43 (58.9%)	24 (63.2%)	
Yes	76 (42.5%)	32 (47.1%)	30 (41.1%)	14 (36.8%)	
Previous chemotherapy in any setting					NA
Yes	179 (100%)	68 (100%)	73 (100%)	38 (100%)	
Neoadjuvant					0.88
No	119 (66.5%)	46 (67.6%)	49 (67.1%)	24 (63.2%)	
Yes	60 (33.5%)	22 (32.4%)	24 (32.9%)	14 (36.8%)	
Adjuvant					0.058
No	105 (58.7%)	47 (69.1%)	36 (49.3%)	22 (57.9%)	
Yes	74 (41.3%)	21 (30.9%)	37 (50.7%)	16 (42.1%)	
Metastatic					0.83
No	4 (2.2%)	2 (2.9%)	1 (1.4%)	1 (2.6%)	
Yes	175 (97.8%)	66 (97.1%)	72 (98.6%)	37 (97.4%)	
Previous hormonotherapy if hormone receptor-positive on primary tumor in any setting (*n* = 128)					0.002
No	9 (7.0%)	8 (18.2%)	1 (1.7%)	0	
Yes	119 (93.0%)	36 (81.8%)	57 (98.3%)	26 (100%)	
Neoadjuvant					0.59
No	115 (97.5%)	36 (100%)	54 (96.4%)	25 (96.2%)	
Yes	3 (2.5%)	0	2 (3.6%)	1 (3.8%)	
Missing	1	0	1	0	
Adjuvant					0.18
No	42 (35.6%)	17 (47.2%)	16 (28.6%)	9 (34.6%)	
Yes	76 (64.4%)	19 (52.8%)	40 (71.4%)	17 (65.4%)	
Missing	1	0	1	0	
Metastatic					0.30
No	13 (11.0%)	6 (16.7%)	6 (10.7%)	1 (3.8%)	
Yes	105 (89.0%)	30 (83.3%)	50 (89.3%)	25 (96.2%)	
Missing	1	0	1	0	
Previous targeted therapy in any setting					0.001
No	17 (9.5%)	1 (1.5%)	7 (9.6%)	9 (23.7%)	
Yes	162 (90.5%)	67 (98.5%)	66 (90.4%)	29 (76.3%)	
Neoadjuvant					0.001
No	141 (87.6%)	51 (76.1%)	61 (93.8%)	29 (100%)	
Yes	20 (12.4%)	16 (23.9%)	4 (6.2%)	0	
Missing	1	0	1	0	
Adjuvant					<0.001
No	119 (73.9%)	33 (49.3%)	59 (90.8%)	27 (93.1%)	
Yes	42 (26.1%)	34 (50.7%)	6 (9.2%)	2 (6.9%)	
Missing	1	0	1	0	
Metastatic					0.40
No	3 (1.9%)	2 (3.0%)	0	1 (3.4%)	
Yes	158 (98.1%)	65 (97.0%)	65 (100%)	28 (96.6%)	
Missing	1	0	1	0	
Previous lines of treatment in metastatic setting					0.94
<5 lines	84 (46.9%)	32 (47.1%)	35 (47.9%)	17 (44.7%)	
≥5 lines	95 (53.1%)	36 (52.9%)	38 (52.1%)	21 (55.3%)	

Comparison among cohorts was performed using chi-square test or Fisher’s exact test for qualitative variables and Kruskal–Wallis test for continuous variables.

NA, not applicable.

### Primary outcome results

**Fig. 2:**
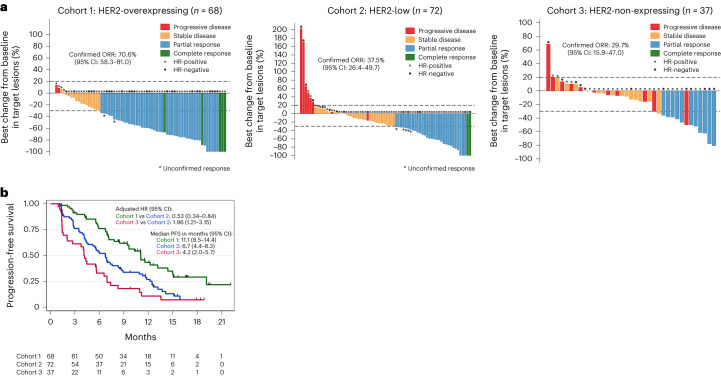
Efficacy of T-DXd per cohort. **a**, Waterfall plot of the best change from baseline in target lesions according to the best objective response per cohort in the FAS population (*n* = 177). The confirmed ORR with T-DXd was 70.6% (*n* = 68, 95% CI 58.3–81) in cohort 1, 37.5% (*n* = 72, 95% CI 26.4–49.7) in cohort 2 and 29.7% (*n* = 37, 95% CI 15.9–47) in cohort 3. The likelihood of confirmed objective response was higher in cohort 1 as compared to cohort 2 (adjusted OR: 3.96, 95% CI 1.78–8.77, *P* = 0.001) and not significantly different between cohort 3 and cohort 2 (adjusted OR: 0.63, 95% CI 0.25–1.54, *P* = 0.30). The adjusted OR and *P* value were derived from a multivariable logistic model taking as reference cohort 2 and adjusted for hormone receptor status, interval from initial diagnosis to metastatic disease, number and type of metastatic site, ECOG performance status and interval from diagnosis of metastatic disease to inclusion. All statistical tests were two-sided. **b**, Kaplan–Meier plot of PFS per cohort in the FAS population (*n* = 177). The median PFS was 11.1 months (95% CI 8.5–14.4) in cohort 1, 6.7 months (95% CI 4.4–8.3) in cohort 2 and 4.2 months (95% CI 2.0–5.7) in cohort 3. PFS was longer in cohort 1 (adjusted HR: 0.53, 95% CI 0.34–0.84, *P* = 0.007) and shorter in cohort 3 (adjusted HR: 1.96 95% CI 1.21–3.15, *P* = 0.006) compared to cohort 2. The adjusted HR and *P* value were derived from a multivariable Cox proportional hazard model taking as reference cohort 2 and adjusted to the same variables used for the confirmed objective response. All statistical tests were two-sided.

In total, 177 patients were included in the full analysis set (FAS) (Fig. 1). The median number of cycles of T-DXd was 12.5 (range, 2–31) in cohort 1, 10 (range, 1–29) in cohort 2 and six (range, 1–26) in cohort 3. The primary endpoint of the study was the confirmed objective response rate (ORR). As of the 19 October 2021 data cutoff, a confirmed objective response occurred in 86 (48.6%) patients: 48 patients (70.6%, 95% CI 58.3–81) in cohort 1 (primary endpoint met), 27 patients (37.5%, 95% CI 26.4–49.7) in cohort 2 (primary endpoint met) and 11 patients (29.7%, 95% CI 15.9–47) in cohort 3 (primary endpoint inconclusive) (Fig. 2a). In addition, seven patients presented an unconfirmed objective response (3.9%). When we looked at the best tumor shrinkage of target lesions, we observed a median reduction of −57.2% (range, −100 to 13.6), −25.3% (range, −100 to 203.2) and −12.5% (range, −80.6 to 68.7) in cohorts 1, 2 and 3, respectively (*P* < 0.0001). In addition, we evaluated the association between confirmed objective response and cohorts adjusting for clinical characteristics listed in the Methods. Patients from cohort 1 presented a higher likelihood of confirmed objective response as compared to cohort 2 (adjusted odds ratio (OR): 3.96, 95% CI 1.78–8.77, *P* = 0.001). The likelihood of confirmed objective response was not significantly different between cohort 3 and cohort 2 (adjusted OR: 0.63, 95% CI 0.25–1.54, *P* = 0.30). We also investigated whether the association between clinical characteristics and confirmed OR differed by cohort. The presence of ≥3 metastatic sites at screening was the only parameter significantly associated with non-response in cohort 2 (*P* = 0.018). Then, we assessed the confirmed ORR in patients who presented a change in the HER2 status of the baseline biopsy as compared to routine care. Among the 14 patients presenting a HER2 overexpression on sample from routine care and who were finally assigned to cohort 2 (HER2-low) after biopsy at baseline, six presented a confirmed objective response (42.9%, 95% CI 17.7–71.1). Confirmed ORRs were 19% (95% CI 5.4–41.9, *n* = 4/21) in patients with HER2 non-expressing mBC who were assigned to cohort 2 (HER2-low) after baseline biopsy and 40% (95% CI 12.2–73.8, *n* = 4/10) in patients with HER2 expression (overexpressed or low) who were assigned to cohort 3 (IHC 0) after baseline biopsy.

**Extended Data Fig. 3:**
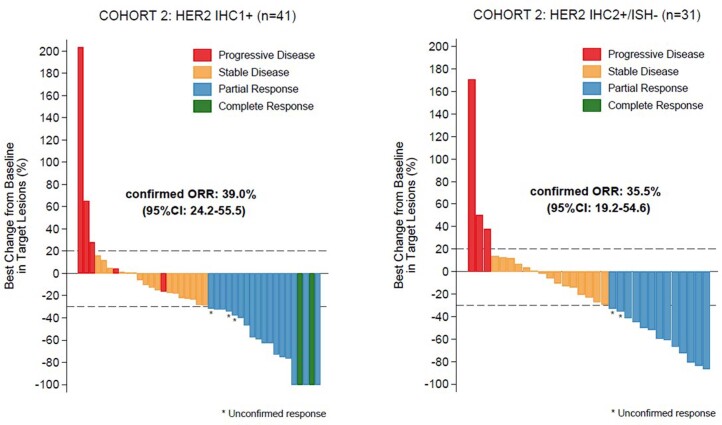
Waterfall plot of the best change from baseline in target lesions according to the best objective response of patients from cohort 2 in FAS population (*n* = 72) according to HER2 status. On the left, patients with HER2 IHC 1+ (*n* = 41) and on the right patients with HER2 IHC 2 + /*ERBB2* ISH- (*n* = 31) treated with T-DXd.

We observed a similar confirmed ORR between patients with HER2 IHC 1+ and IHC 2+/*ERBB2* ISH-negative in cohort 2 (Extended Data Fig. 3).

### Secondary outcome results

**Extended Data Fig. 4:**
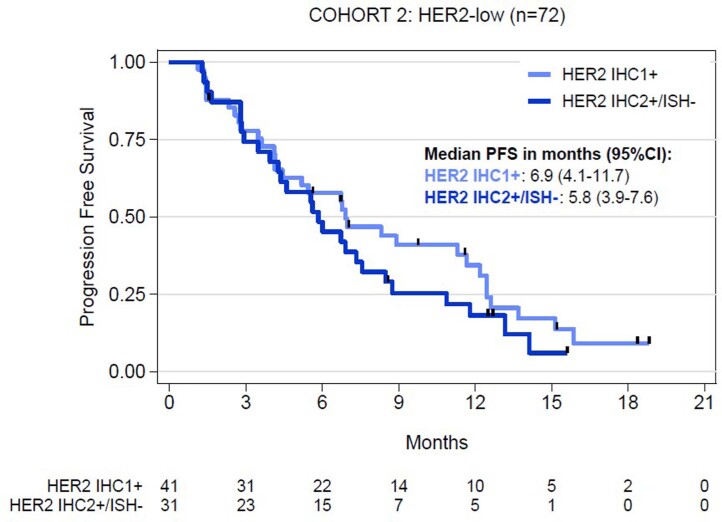
Kaplan-Meier plot of PFS in the FAS population from cohort 2 (*n* = 72) according to HER2 status. The median PFS was 6.9 months (95% CI 4.1-11.7) in patients with IHC 1+ (*n* = 41) and 5.8 months (95% CI 3.9-7.6) in patients with IHC 2 + /*ERBB2* ISH- mBC (*n* = 31).

The secondary endpoints were duration of response, PFS, OS, clinical benefit rate and safety. In 93 patients with a confirmed or unconfirmed objective response, the median duration of response was 9.7 months (95% CI 6.8–13) in cohort 1, 7.6 months (95% CI 4.2–9.2) in cohort 2 and 6.8 months (95% CI 2.8–not reached) in cohort 3. After a median follow-up of 15.6 months (95% CI 12.6–16.7), the median PFS was 11.1 months (95% CI 8.5–14.4) in cohort 1, 6.7 months (95% CI 4.4–8.3) in cohort 2 and 4.2 months (95% CI 2.0–5.7) in cohort 3. In the multivariable analysis adjusted for clinical characteristics, cohort 1 was associated with longer PFS (adjusted HR: 0.53, 95% CI 0.34–0.84, *P* = 0.007) and cohort 3 with shorter PFS (adjusted HR: 1.96, 95% CI 1.21–3.15, *P* = 0.006) as compared to cohort 2 (Fig. 2b). When we assessed the association between clinical characteristics and PFS by cohort, we found that an interval from diagnosis of metastatic disease to inclusion of more than 24 months was associated with a longer PFS (HR 24–60 months versus 0–24 months: 0.35, 95% CI 0.16–0.79; HR >60 months versus 0–24 months: 0.24, 95% CI 0.10–0.57; *P* = 0.002) in cohort 1; the presence of ≥3 metastatic sites at screening with a shorter PFS (HR: 2.24, 95% CI 1.27–3.93, *P* = 0.004) and HR expression with a longer PFS (HR: 0.48, 95% CI 0.26–0.92, *P* = 0.022) in cohort 2. Finally, an ECOG performance status of 1 at screening was associated with a shorter PFS (HR: 2.13, 95% CI 1.02–4.44, *P* = 0.037) in cohort 3. A similar PFS was observed between patients with HER2 IHC 1+ and IHC 2+/*ERBB2* ISH-negative in cohort 2 (Extended Data Fig. 4). PFS for each cohort according to HR status is described in Supplementary Fig. 1. After a median follow-up of 14.1 months (95% CI 13.2–15.2), the median OS was not reached (95% CI 16.7–not reached) in cohort 1, not reached (95% CI 11.5–not reached) in cohort 2 and 11.6 months (95% CI 8.3–17.3) in cohort 3. The clinical benefit rate was 85.3% (95% CI 74.6–92.7) in cohort 1, 56.9% (95% CI 44.7–68.6) in cohort 2 and 35.1% (95% CI 20.2–52.5) in cohort 3.

In total, 145 patients (81%) permanently discontinued treatment: 49 (72.1%) in cohort 1, 61 (83.6%) in cohort 2 and 35 (92.1%) in cohort 3. The reason for discontinuation was disease progression in 125 (86.2%) patients and toxicity in 13 (9%) patients. Adverse events were consistent with previous data^10^ and are reported in Extended Data Tables 1 and 2. The most common adverse effects ≥grade 3 were neutropenia (12%), fatigue (8%) and vomiting (6%), consistent with the toxicity profile of TOP1 inhibitors. Nine patients (5%) presented interstitial lung disease or pneumonitis, all of them grade 1 or grade 2. Three patients (1.7%) presented ejection fraction decreased (one grade 3). Three patients (1.7%) presented a grade 5 adverse effect.

### HER2 expression patterns and treatment response

**Fig. 3:**
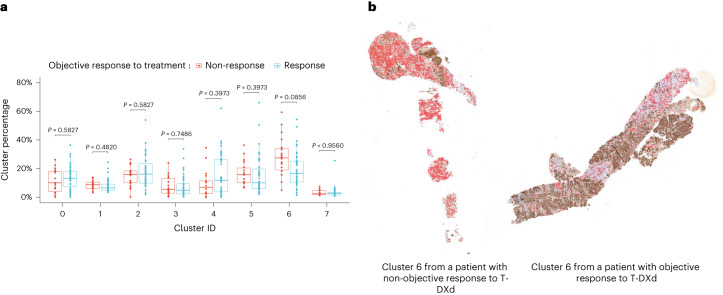
HER2 expression patterns and treatment response. **a**, Clusters’ relative percentage according to T-DXd sensitivity in cohort 1 (HER2-overexpressing). For each patient, the corresponding HER2 pathology slide (*n* = 61) was divided into 64 × 64-px non-overlapping patches that were classified into eight clusters using a Mini-Batch *K*-means algorithm. The following box plot illustrates the relative percentage of each cluster in each slide and its association with the confirmed objective response or non-response to T-DXd. Box center lines, box ranges, whiskers and dots indicate medians, quartiles, 1.5× IQR and outliers, respectively. Cluster 6 presented a significant association with non-response to T-DXd (*P* = 0.011, FDR-adjusted *P* = 0.086). *P* values were calculated using the Mann–Whitney *U*-test and adjusted for multiple hypothesis testing using the Benjamini–Hochberg method. All statistical tests were two-sided. **b**, One pair of pathology slides that shows cluster 6 in red. A patient with resistance (left) and sensitivity (right) to T-DXd. Cluster 6 comprised HER2-negative areas with moderate cell density (mean value of 30% (95% CI 25–34)), containing mainly fibroblasts and immune cells (mean value of 56% (95% CI 50–62) and 27% (95% CI 22–32), respectively).

**Extended Data Fig. 5:**
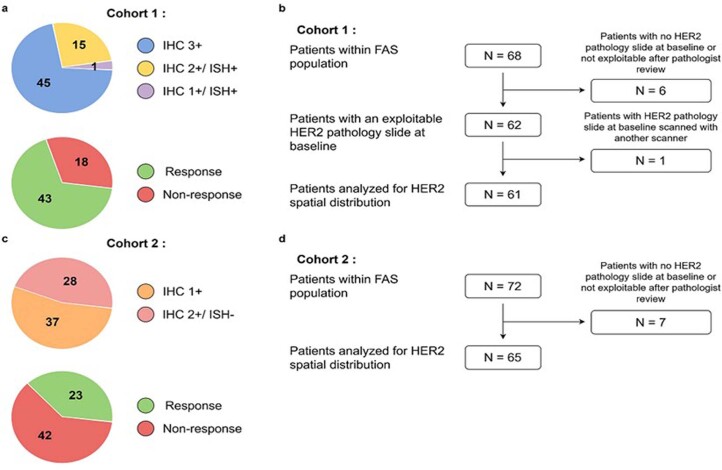
Dataset description for HER2 expression spatial analysis. **a**. Distribution of HER2 IHC score and confirmed objective response in the dataset in cohort 1 (HER2-overexpressing). The numbers represent the number of patients analyzed (n = 61). **b**. Description of the patient set considered for HER2 spatial distribution analysis in cohort 1. **c**. Distribution of HER2 IHC score and confirmed objective response in the dataset in cohort 2 (HER2-low). The numbers represent the number of patients analyzed (n = 65). **d**. Description of the patient set considered for HER2 spatial distribution analysis in cohort 2.

**Extended Data Fig. 6:**
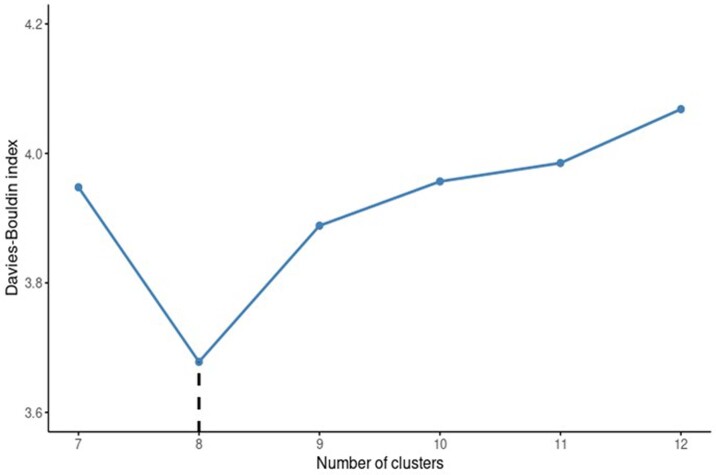
Identifying an optimal number of clusters in cohort 1. The Davies-Bouldin index was computed from Mini-Batch K-Means clustering using a number of clusters ranging from 7 to 12. This index represents how the clusters are similar to each other, with a lower value pointing toward a better segmentation. Minimum is highlighted on the graph at 8 clusters.

We further examined HER2 expression patterns in the three cohorts as an exploratory objective. We first assessed whether HER2 spatial distribution predicts drug response in patients from cohort 1 (HER2-overexpressing mBC; *n* = 61) (Extended Data Fig. 5). Machine learning analyses indicated that HER2 slides could be segmented into eight clusters using an unsupervised clustering algorithm (Extended Data Fig. 6). Intensity of diaminobenzidine (DAB) staining (HER2 expression) and cell density were two core features that drove the unsupervised clustering (Supplementary Fig. 2). We compared non-responders to responders based on the percentage of each of the eight identified clusters, finding that non-responders had a greater percentage of cluster 6 in their tumor (*P* = 0.011, false discovery rate (FDR)-adjusted *P* = 0.086), with no other cluster showing a statistically significant difference among groups (Fig. 3). Cluster 6 was characterized by a low HER2 staining (median, 0.19; interquartile range (IQR), 0.10–0.47) and a moderate cell density (37% of patches without cells and 34% with one nucleus). Cells present in cluster 6 patches were mainly fibroblasts and immune cells (mean value of 56% (95% CI 50–62) and 27% (95% CI 22–32), respectively). In 28 of 60 (47%) patients, cluster 6 also contained tumor cells with a mean value of 40% (95% CI 24–56), 48% (95% CI 33–63) and 12% (95% CI 0–23) of HER2 IHC 0, IHC 1+ and IHC 2+, respectively (Supplementary Table 1). We then used this model to analyze 65 HER2 pathology slides from cohort 2 (HER2-low mBC; Extended Data Fig. 5). No significant association with T-DXd efficacy was observed (Supplementary Fig. 3). Next, we trained a new model using data from 65 patients from cohort 2 (HER2-low mBC; Methods) (Extended Data Fig. 5). We did not find a significant association between the identified clusters and T-DXd response (Supplementary Fig. 4).

**Extended Data Fig. 7:**
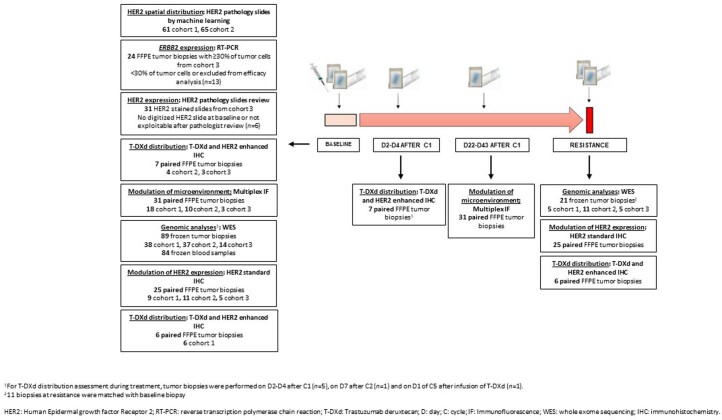
Tissue and blood samples per time point and cohort used for translational analyses. Cohort 1: HER2-overexpressing (HER2 IHC 3+ or *ERBB2* ISH+) mBC, cohort 2: HER2-low (HER2 IHC 2+/*ERBB2* ISH- or IHC 1+) mBC, cohort 3: HER2 non-expressing (HER2 IHC0) mBC.

We then assessed whether levels of HER2 expression could predict drug response in patients from cohort 3 (HER2 IHC 0, *n* = 37). We first evaluated *ERBB2* gene expression by reverse transcription polymerase chain reaction (RT–PCR) in 24 tumor samples obtained at baseline with ≥30% of tumor cells (Extended Data Fig. 7). We found that 5 of 14 (35.7%, 95% CI 12.8–64.9) patients with *ERBB2* expression below the median presented a confirmed objective response as compared to 3 of 10 patients (30%, 95% CI 6.7–65.2) with *ERBB2* expression above the median. HER2-stained slides obtained at baseline biopsy from 31 patients in cohort 3 were reviewed by two pathologists (Extended Data Fig. 7). Some level of HER2 expression was detected in 15 samples (8 ‘ultra-low’ (defined in the Methods section) and 7 IHC 1+). A confirmed objective response was observed in 6 of 15 (40%, 95% CI 16.3–67.7) patients with detectable HER2 expression and in 4 of 16 (25%, 95% CI 7.3–52.4) patients without detectable HER2 expression.

### T-DXd mechanisms of action

**Fig. 4:**
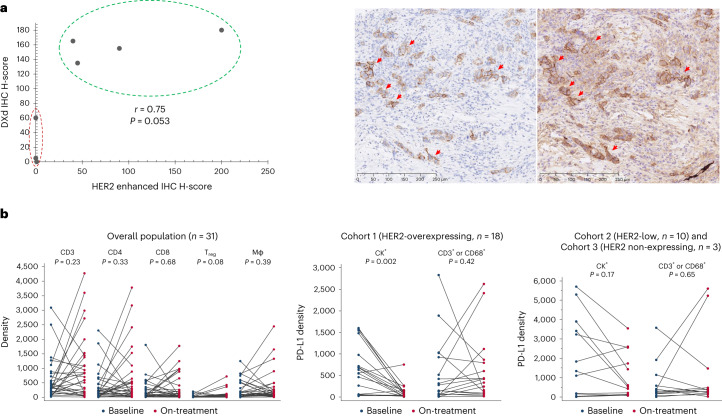
Mechanisms of action of T-DXd. **a**, Illustration of the correlation between T-DXd distribution and HER2 expression. T-DXd was determined by IHC using an Ac anti-DXd (H-score) and HER2 by an enhanced protocol of IHC (H-score) in seven paired samples at baseline and during treatment. The staining was performed in one sample per case. The correlation was calculated by Pearson correlation coefficient, which showed a moderate correlation (*r* = 0.75, *P* = 0.053). *P* value was calculated using a two-sided Pearson correlation test. On the bottom, a pathology slide that shows HER2 staining (red arrows) on the left and T-DXd staining (red arrows) on the right. **b**, Illustration of the immune microenvironment modulation by T-DXd. Tumor biopsies at baseline and days 22–43 after cycle 1 of T-DXd were assessed by multiplex immunofluorescence (*n* = 31). No quantitative modulation of the immune microenvironment by T-DXd in the overall population (*n* = 31) was observed. There was a significant decrease in PD-L1 expression presumably due to the cytotoxic effect of T-DXd on tumor cells (CK^+^/PD-L1^+^) in patients with HER2-overexpressing mBC (*n* = 18, *P* = 0.002). Immune cells, represented by CD3^+^/PD-L1^+^ or CD68^+^/PD-L1^+^, did not show a decrease during treatment in cohort 1 (*n* = 18, *P* = 0.42). No significant decrease of PD-L1^+^ tumor (*P* = 0.17) or immune cells (*P* = 0.65) was observed in patients with HER2-low and HER2-non-expressing mBC (*n* = 13) during treatment. Blue bullets and red bullets represent at-baseline and on-treatment samples, respectively. *P* values were calculated using the Wilcoxon matched-pairs signed-rank test. All statistical tests were two-sided.Mφ, macrophage; T_reg_, regulatory T cell.

**Extended Data Fig. 8:**
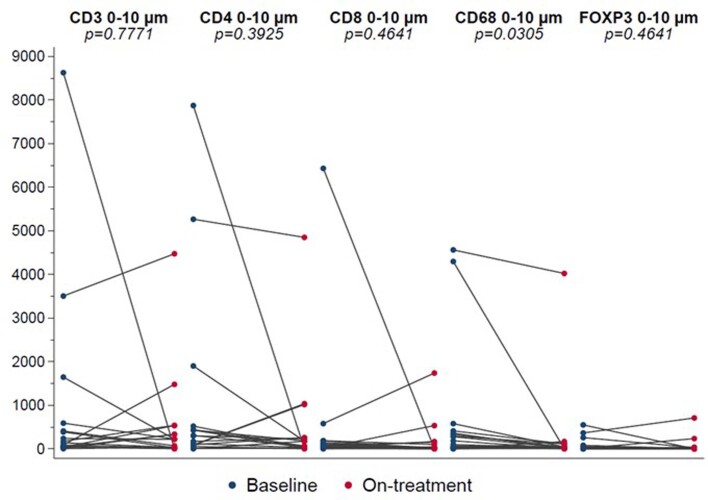
Immune cell density at 0 to 10 µm of tumor cells, in baseline and on treatment tumor biopsies in cohort 1 (HER2-overexpressing, *n* = 18). A significant decrease in tumor cell-proximate macrophage was observed during treatment (FDR-adjusted *p* = 0.0305). Blue bullets and red ones represents at baseline and on-treatment samples, respectively. *P*-values were calculated using the Wilcoxon matched-pairs signed-rank test and adjusted for multiple hypothesis testing using the Benjamini-Hochberg method. All statistical tests were two sided.

We further explored T-DXd distribution (exploratory objective) in seven paired biopsies obtained at baseline and during treatment (Extended Data Fig. 7). Tumor cells with a high level of HER2 expression presented strong T-DXd staining (Fig. 4a). Conversely, three samples classified HER2 IHC 0 by an enhanced protocol (Methods) presented no or very few T-DXd staining (Pearson correlation coefficient *r* = 0.75, *P* = 0.053). Two of these three patients with low level of T-DXd distribution presented a confirmed partial response with a PFS of 17.8 months (censored patient) and 12 months, respectively. We then investigated whether T-DXd modulates the immune microenvironment (exploratory objective) in 31 patients from the three cohorts (Extended Data Fig. 7). No quantitative modulation of immune cells by T-DXd was detected at week 3 or week 6 after drug administration (Fig. 4b). A significant decrease in PD-L1 expression was observed in patients in cohort 1 (*n* = 18, *P* = 0.002), presumably due to the cytotoxic effect of T-DXd on tumor cells (cytokeratin (CK)^+^/programmed death-ligand 1 (PD-L1)^+^) (Fig. 4b). A decrease in tumor-cell-proximate macrophages (0–10 µm) was also observed in cohort 1 (*n* = 18, FDR-adjusted *P* = 0.0305; Extended Data Fig. 8).

### Mechanisms of resistance to T-DXd

**Fig. 5:**
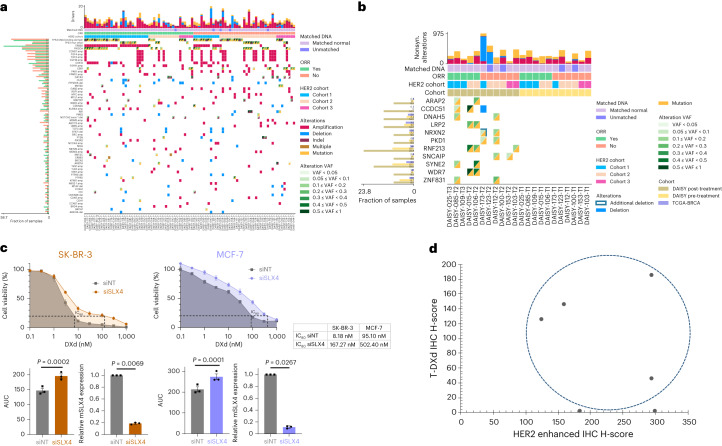
Mechanisms of resistance to T-DXd. **a**, Oncoplot of driver mutations and CNAs identified in at least 3% of tumor biopsies at baseline (*n* = 89). Blood samples were available for analyses in 84 patients. If a gene has at least one driver mutation or CNA in at least 3% of pretreatment biopsies, any other driver alteration of the same gene is shown, regardless of its frequency. **b**, Oncoplot of acquired genomic alterations identified at resistance (*n* = 11). Eleven biopsies at resistance (on the left) were matched with pretreatment biopsies (on the right) from the same patient. Only genes that were not altered in any of the 11 pretreatment samples and that acquired an alteration in at least two samples at resistance (three samples in case all events were CNAs) are shown. The left histogram depicts the frequency at which the gene was altered in the pretreatment (*n* = 89), resistance (*n* = 21) and TCGA-BRCA (*n* = 684) cohorts for comparison. **c**, Dose–response survival curves of SK-BR-3 and MCF-7 cell lines transfected with non-targeting or *SLX4*-targeted siRNAs (siNT or siSLX4, respectively) and exposed to DXd at the indicated doses for 5 days. Area under the curve (AUC) and IC_80_ values were determined for each condition. Data are mean surviving fractions ± s.e.m., *n* = 3 experiments for both cell lines. Statistical analysis was performed using Welch’s *t*-test (two-tailed). **d**, Illustration of T-DXd uptake and HER2 expression at resistance (*n* = 6). T-DXd was determined by IHC using an Ac anti-DXd (H-score) and HER2 by an enhanced protocol of IHC (H-score). T-DXd was observed in four of six patients whose biopsy at resistance was done ≤6 weeks after last T-DXd infusion.

To identify mechanisms of primary and secondary resistance (exploratory objective), we performed whole-exome sequencing (WES) of frozen tumor tissue obtained at baseline (*n* = 89: 38 from cohort 1; 37 from cohort 2; and 14 from cohort 3) and at resistance (*n* = 21: five from cohort 1, 11 from cohort 2 and five from cohort 3). Eleven biopsies at resistance were matched with baseline biopsies (Extended Data Fig. 7). Figure 5a reports the driver somatic mutations and copy number alterations (CNAs) at baseline according to sensitivity to T-DXd. With the exception of *ERBB2* amplifications, we did not observe a significant association between driver alterations and upfront resistance (FDR-adjusted *P* > 0.54). *ERBB2* hemizygous deletion was detected in six of 89 (7%) patients at baseline. Interestingly, four of these patients did not respond to T-DXd (three in cohort 2 and one in cohort 3). We next explored which genomic alterations were acquired at resistance by comparing the genomic landscape of 11 pairs of biopsies obtained at baseline and at resistance. We found that 11 genes presented an alteration acquired at resistance in at least 2 of 11 pairs while not being mutated in any of the 11 matched baseline samples (Fig. 5b). Three of twenty-one (14%) samples obtained at resistance to T-DXd presented a *SLX4* mutation: one of them was not observed in the matched baseline sample; the second one was present in the biopsy at baseline; and, for the third one, the biopsy at baseline was not available. Two of these mutations were ranked as deleterious according to CADD and SIFT; however, no evidence of loss of second allele was found. *SLX4* M1591L was observed in the post-treatment sample of DAISY-109 patient. Detailed investigations of the mutation filtering performed by the variant caller (Methods) revealed that the mutation is incorrectly flagged in the baseline sample due to the presence of three nearby artifactual mutations. Additionally, rs149126845 missense variant (*SLX4* K458E) was detected in the post-treatment sample DAISY-098-T2, but, due to the absence of a matched blood sample and relatively high frequency of this mutation in the Genome Aggregation Database (gnomAD), we decided not to include this variant. Conversely, *SLX4* mutations were present in three of 89 (3%) pretreatment biopsies and 1.5% in The Cancer Genome Atlas (TCGA) breast cancer (BRCA). We assessed cell viability in two breast cancer cell lines depleted for *SLX4* and treated with different doses of DXd for 5 days. DXd concentration needed to achieve 80% inhibition (IC_80_) was increased 20-fold (8.18 nM versus 167.27 nM) in *SLX4*-silenced SK-BR-3 cells and fivefold (95.1 nM versus 502.4 nM) in *SXL4*-silenced MCF-7 cells (Fig. 5c).

Finally, we investigated how HER2 expression and T-DXd distribution were modulated at resistance as compared to pretreatment (Extended Data Fig. 7). Thirteen of 20 (65%, 95% CI 40.8–84.6) patients presented a decrease of HER2 tumor expression after resistance to the drug, namely three IHC 3+ (two to IHC 2+ and one to IHC 1+), six IHC 2+ (three to IHC 1+ and three to IHC 0) and four from IHC 1+ to IHC 0. Intratumoral uptake of T-DXd was observed in four of six patients from cohort 1 at resistance (biopsy within 6 weeks after last infusion), suggesting that T-DXd can still be distributed to cancer cells in a subset of patients at resistance (Fig. 5d). All patients presenting tumoral uptake of T-DXd at resistance had HER2 IHC 3+ or 2+ on biopsy done at resistance.

## Discussion

We report converging evidence that HER2 expression is a determinant of T-DXd efficacy. Specifically, the PFS rates were significantly different across the three cohorts of patients; T-DXd uptake was different according to HER2 levels; and HER2 expression decreased at resistance. A previous study suggested that HER2 quantitative continuous score (QCS) could potentially predict outcome to T-DXd in patients with HER2-low mBC^15^. Previous clinical trials showed that tumor responses were similar in HER2 IHC 1+ and IHC 2+ subgroups^14,16^. This finding was confirmed in the DAISY trial, suggesting that IHC may not be the optimal test to define the boundary of HER2 expression to predict efficacy in patients with HER2-low mBC.

Although T-DXd anti-tumor activity increased when HER2 expression was high, modest anti-tumor activity was also observed in patients with HER2 IHC 0. This suggests that very low levels of HER2 could allow uptake of T-DXd and/or that drug efficacy could be partially mediated by HER2-independent mechanisms. A study that involved 18 pathologists showed a low level of concordance (26%) to score HER2 IHC 0 and 1+ (ref. ^17^). In our study, 48% (*n* = 15) of HER2 slides IHC 0 had detectable HER2 expression, either ‘ultra-low’ or IHC 1+, in an external pathology review. In another study^18^, 67% of 364 breast cancer cases classified HER2 IHC 0 had detectable HER2 expression by using quantitative immunofluorescence. Collectively, these studies suggest that HER2 IHC 0 includes a large number of cancers with some level of HER2 expression and that a subset of these patients is sensitive to T-DXd. This provides a strong rationale for ongoing trials such as DB-06, testing efficacy of T-DXd in patients with HER2 ‘ultra-low’ mBC^19^. This may expand the population deriving benefit from T-DXd and outlines the importance of the emergence of optimized HER2 assays. Notably, we did not detect any efficacy difference based on *ERBB2* gene expression within the group of patients with HER2 IHC 0 mBC, further suggesting potential drug activity in patients with very low, if any, expression of *ERBB2*. The presence of free payload, after cleavage of T-DXd linker, could be another possible explanation for T-DXd efficacy in patients with HER2 IHC 0 mBC^20^. The assessment of efficacy according to *ERBB2* gene expression in patients with HER2-low mBC (IHC 1+ or IHC 2+/ISH-negative) will be performed in the future. We decided not to conduct this analysis initially, as a previous report suggested that *ERBB2* mRNA levels showed little inter-patient variability in HER2-non overexpressing BC^21^. In addition, similar efficacy was observed in HER2 1+ versus HER2 2+/ISH-negative mBC.

Although numbers are small and should be interpreted cautiously, the confirmed objective response was slightly lower in patients with HER2-overexpressing mBC who became HER2-low after baseline biopsy. This finding could be relevant to interpret the DB-02 and DB-03 studies where patients received multiple lines of prior anti-HER2 therapies.

While efficacy of second-generation ADCs, such as T-DM1, was strongly associated with target expression^22^, this has not been shown with the latest generation of ADCs. For example, TROP2 expression was not predictive for the efficacy of the TROP2-targeting ADC sacituzumab govitecan in the ASCENT trial, although the number of patients with TROP2-low expression was small, and the analyses did not allow definitive conclusion^23^. Efficacy of patritumab deruxtecan was observed across patients with mBC with a broad spectrum of HER3 expression^24,25^, although most exhibited high levels of HER3 tumor expression.

We observed a lower median PFS in the DAISY trial than in previously reported studies testing the efficacy of T-DXd. PFS was longer in the DB-02 (ref. ^12^) trial than in the DAISY trial (17.8 months versus 11.1 months). This could be explained by a lower number of prior therapies (median of two previous lines of therapy in metastatic setting) and a better general condition as measured by the ECOG performance status at inclusion (ECOG performance status = 0: 56% versus 31%) in DB-02. The DB-01 trial also showed longer PFS as compared to our study (19.4 months)^11^. The reasons for this difference are unclear. In DB-01, 50% of patients were in good general condition (ECOG performance status = 0). However, patients were as heavily pretreated as in the DAISY trial (median of six previous lines of therapy in metastatic setting). Efficacy results from DB-03 are not directly comparable to our study because patients did not receive prior T-DM1 (ref. ^26^). In the population of patients with HER2-low mBC, the median PFS was slightly higher in the DB-04 trial^14^ as compared to our study (9.9 months versus 6.7 months). The proportion of patients with hormone receptor-positive breast cancer (89% versus 79%), the general condition (ECOG performance status = 0: 54% versus 45%) and the number of prior therapies (median of three previous lines of therapy in metastatic setting in DB-04) were different between the two studies and could explain the difference in outcome.

The safety profile of T-DXd in our study was similar to previous reports^11,14^. The most common adverse effects ≥grade 3 were neutropenia, fatigue and vomiting, consistent with that of TOP1 inhibitors. Most cases of interstitial lung disease or pneumonitis and decreased ejection fraction in this trial were mild or moderate, and the overall incidence was consistent with the ones reported in previous studies^14,27^.

Preclinical data showed that T-DXd increased tumor-infiltrating dendritic cells and CD8^+^ T cells in an immunocompetent mouse model inoculated with human HER2-expressing colon cancer cells^28^. Based on these data, several clinical trials assessing T-DXd in combination with immune checkpoint inhibitors are ongoing^29,30^. Our study did not validate these findings, but there was no decrease of CD8^+^ T cells, in contrast to previous observations with systemic chemotherapy^31^.

Although HER2 expression substantially decreased at the time of resistance to T-DXd, there is no robust evidence that a reduction of T-DXd uptake is the dominant mechanism of resistance in the present study. Indeed, T-DXd was still distributed in the cancer cells in four of six patients at the time of resistance. Unfortunately, no quantitative comparison of T-DXd uptake could be done during treatment and at resistance. In a case report, resistance to sacituzumab govitecan, an ADC targeting TROP2, was associated with *TROP2* mutation and defective plasma membrane localization^32^, suggesting that the ADC target could be involved in resistance. We identified mutations of *SLX4* at resistance in three of 21 (14%) patients. *SLX4* encodes a DNA repair protein that regulates structure-specific endonucleases and might have a role in resistance to TOP1 inhibition^33,34^. The TCGA reports *SLX4* mutations in 1.5% of primary breast cancer, the majority hormone receptor-positive/HER2-negative ductal carcinoma^35^. Another study reported *SLX4* mutations in 1.3% of HER2-negative mBC (50% hormone receptor-positive)^36^. Although corroborated with in vitro studies, the nature of *SLX4* mutations found in the DAISY trial remains to be determined. In contrast to previous data, we did not detect *TOP1* mutations at the time of resistance^32^.

Our trial has several limitations. We did not include negative controls for HER2 expression assessment at resistance; the number of samples analyzed was small; and there was no validation cohort for several of the translational objectives.

The present study suggests that HER2 is a determinant of sensitivity to T-DXd, although modest anti-tumor activity was also observed in a small subset of patients whose cancer did not express HER2, suggesting other mechanisms of action. Resistance to T-DXd may occur at different levels, potentially involving decrease of HER2 expression, alterations of the cytotoxic effect of DXd and the tumor microenvironment. These data indicate that precision medicine approaches based on molecular analyses will be necessary to optimize treatment after resistance to T-DXd.

## Methods

### Patients and study design

DAISY (NCT04132960) is a prospective, phase 2, open-label, clinical trial that assessed T-DXd efficacy in patients with mBC. The DAISY trial complies with all relevant ethical regulations, overseen by the board/committee and institution that approved the study protocol. DAISY trial was approved by the French ethics committee (CPP), Île-de-France, on 5 September 2019 and the French health authorities (ANSM) on 8 July 2019. The first patient was enrolled on 4 November 2019 and the last one on 3 March 2021 in 15 study centers in France. The study design is reported in Extended Data Fig. 1. The first and last versions of the protocol are provided in the Supplementary Information. Patients with mBC were eligible if they had received at least one line of chemotherapy in the metastatic setting, had at least one non-bone metastatic lesion accessible to biopsy and had signed the informed consent for biopsies throughout the study. Both sexes were eligible based on self-report. Mandatory and optional biopsies are described in the protocol (Annex 1 and Annex 2). The biopsy at baseline could be skipped if a biopsy collected within 3 months before inclusion was available. Patients were assigned to three cohorts according to HER2 level expression determined by standard IHC, as previously reported^37^, on samples obtained at baseline biopsy. Patients with HER2-overexpressing mBC had to be pretreated with taxane and to be resistant to trastuzumab and T-DM1. Patients with HER2-low or HER2 IHC 0 tumors had to be pretreated with anthracyclines and taxanes. Patients with tumor-expressing hormone receptors had to be resistant to endocrine therapy and CDK4/6 inhibitors. No safety monitoring board was involved in the study.

### Treatments and follow-up

After signature of the informed consent, patients were treated with T-DXd intravenously 5.4 mg kg^−1^ every 3 weeks until disease progression or unacceptable toxicity, as defined by the investigator. Recommendations for dose reductions are described in the V1 of the protocol (Annex 1). Treatment efficacy was monitored by a computed tomography (CT) scan every 6 weeks during the initial 12 months and every 12 weeks thereafter. The CT scan was repeated at least 4 weeks after assessment of a partial response or a complete response. Response Evaluation Criteria in Solid Tumors (RECIST) version 1.1 were used to determine response and progression^38^. Toxicity data were collected at each visit and classified according to the National Cancer Institute Common Terminology Criteria for Adverse Events (CTCAE) version 5.0.

### IHC

For cohort allocation, HER2 status was determined by a GEFPICS trained pathologist^39^ on the biopsy performed at baseline (Extended Data Fig. 2). Estrogen and progesterone receptor status were determined locally, with a cutoff for positivity set at 10% of tumor cells. HER2 status was defined according to the last version of the American Society of Clinical Oncology (ASCO)/College of American Pathologists (CAP) guidelines^37^. ‘Ultra-low’ HER2 category was defined as cases showing a faint to weak incomplete membrane staining in less than 10% of tumor cells (that is, less than 1+, classified in the IHC 0 category following the ASCO/CAP guidelines^37^). HER2 staining on biopsies at progression was performed centrally at Gustave Roussy using the 4B5 pre-diluted kit (VENTANA pathway HER2, clone: 4B5, Roche Diagnostics), according to the manufacturer’s protocol. Twenty-five pairs of tumor biopsies obtained at baseline and progression were assessed (Extended Data Fig. 7).

Regarding T-DXd distribution during treatment, 10 paired baseline and on-treatment tumor biopsies were initially selected. Three pairs were not analyzed owing to the lack of tumor cells. Seven pairs of tumor biopsies, four from cohort 2 and three from cohort 3 (days 2–4 cycle 1, *n* = 5; day 7 cycle 2, *n* = 1; day 1 cycle 5, *n* = 1), were stained. Staining was done for HER2 with VENTANA anti-HER2/neu (4B5) rabbit monoclonal primary antibody (VENTANA pathway HER2, clone: 4B5, Roche Diagnostics) according to the manufacturer’s instructions, and, when necessary, the enhanced HER2 protocol was employed to detect low level of HER2 expression. For the enhanced HER2 protocol, OptiView DAB IHC Detection Kit (Roche Diagnostics) was used instead of ultraView Universal DAB Detection Kit (Roche Diagnostics). HER2 was defined according to the last version of the ASCO/CAP guidelines^37^. Tissue sections were stained for DXd-IgG using primary antibody against DXd (antiXAFG5737-1A3-ocChimera, Daiichi Sankyo) with Leica BOND RX automated slide stainer (Leica Biosystems). Rabbit isotype control antibody (PA0777, Leica Biosystems) was used as negative reagent control. Anti-DXd was raised against a part of DXd and can recognize free DXd. However, in formalin-fixed, paraffin-embedded (FFPE) samples, the intracellular, cleaved, free DXd is expected to be washed out during sample preparation and IHC procedure because DXd does not contain formaldehyde-sensitive group (that is, -NH2). The IHC procedure washed away the free DXd, which resulted in the DXd-IgG primarily detecting T-DXd. The distribution and the percentage of DXd-IgG-positive cells in total tumor cells was evaluated. In addition to the seven pre-/on-treatment biopsies, DXd staining was also performed in six paired tissue biopsies obtained at baseline and resistance from cohort 1 (Extended Data Fig. 7).

Multiplex immunofluorescence was performed with the Ultivue kit containing the Immuno8 FixVUE panel composed of eight pre-diluted antibodies (twice four barcoded markers) + DAPI ready to use. The antibodies were directed against CD3 (clone BC33), CD4 (clone SP35), CD8 (clone C8/144B), CD68 (clone KP-1), FoxP3 (clone 236A/E7), PD-1 (clone CAL20), PD-L1 (clone 73-10) and PanCK/SOX10 (clone AE1/AE3/BC34). Thirty-one paired FFPE tumor biopsies obtained at baseline and on days 22–43 after cycle 1 were stained (18 cohort 1, 10 cohort 2, three cohort 3; Extended Data Fig. 7). After each detection cycle and hematoxylin, eosin and saffron (HES) staining, slides were imaged using the Akoya Biosciences PhenoImager HT. For each sample, the three whole slide images (WSIs) were stacked in one by Ultivue. The resulting stacked WSIs were analyzed in QuPath 0.3.2 (ref. ^40^) software. Regions of interest (ROIs) were manually delineated by a pathologist (M.L.T.). Inside these regions, tissue was automatically detected using a trained classifier. The number of stained cells per square millimeter of analyzed tissue was determined for each marker. In addition, the distance between each cell and the nearest CK^+^ cell was computed.

### HER2 spatial distribution analysis by machine learning

Slides stained for HER2 expression collected at baseline from cohort 1 were digitalized and analyzed through an unsupervised clustering algorithm (*n* = 61; seven slides were not exploitable or could not be digitalized with the appropriate scanner; Extended Data Fig. 5). Unsupervised clustering algorithms aimed to identify groups of patches sharing similar features across the slides (clusters). A pathologist (I.J.G.) annotated the ROIs in the slides to discard biopsies without tumor tissue and tissue regions outside viable tumor. We contoured each tissue within these annotated areas using morphological operations. We downsampled the slides by a factor of 8 in each dimension to reduce image size and applied a grayscale conversion. We then subtracted the local average over a window size of 30 × 30 from each pixel to retrieve an image near-zero mean and computed the law texture energy measures. Finally, we applied a binary threshold on the spot texture map (above 20) with a flood fill algorithm to discard eventual artifacts (blurry regions and ink stains) and extract final tissue contours. We isolated main tissues by enforcing a minimum area criterion (above 1,500) to remove noisy elements introduced by the pre-processing. We chose a patch size of 64 × 64, without overlap, to carry the analysis at the level of a few cells and removed black and white patches by removing patches with over 70% of RGB values below 2 and 80% of RGB values above 250. Following Lu et al.^41^, we extracted 1,024 visual appearance descriptors by applying a ResNet50 model truncated after the third residual block and pre-trained on ImageNet. We applied Mini-Batch K-means^42^ to extract clusters across all the slides from the patch descriptors, normalized by their mean and s.d. To determine the optimal number of clusters, we computed the Davies–Bouldin index within a range around nine clusters (from seven to 12)^43^. A segmentation into eight clusters minimized the score on this interval (Extended Data Fig. 6), and so we selected this value. For each slide *i* containing *N*_*i*_ patches, we computed the cluster assignment of each patch ${{\rm{L}}}_{{{\rm{n}}}_{{\rm{i}}}}\in \{0\ldots 7\}$ using the trained clustering algorithm. We retrieved a vector of eight features ${{\rm{V}}}_{{\rm{i}}}\in {\left[0,1\right]}^{8}$ for each slide by counting the proportion of patches for each label:

$${{\rm{V}}}_{{\rm{i}}}\left[{\rm{k}}\right]=\frac{1}{{N}_{i}}\mathop{\sum }\limits_{{{\rm{n}}}_{{\rm{i}}}=1}^{{{N}}_{{i}}}{1}_{\left\{{{\rm{L}}}_{{{\rm{n}}}_{{\rm{i}}}}={\rm{k}}\right\}}\,{\rm{for}}\; {\rm{k}}\in \{0\ldots 7\}$$

The clusters were further analyzed on the ground of nuclei statistics toward the design of interpretable markers using an unsupervised nuclei segmentation algorithm^44^. To adapt the heterogeneous staining condition of DAISY slides, we added several modifications to the original methodology. We added color jittering to the consistency loss as well as instance normalization instead of batch normalization while retraining on the same HER2 public data to help to improve the model generalization. Then, we implemented a new stain deconvolution method better tailored to IHC imaging^45^. Lastly, we selected the best model to predict nuclei masks for DAISY data under the guidance of an expert pathologist selecting the checkpoint to be used. The nuclei were then segmented on 256 × 256-pixel (px) overlapping patches, with 50% overlap obtained with the same pre-processing described for the clustering. To interpret clustering decision, we computed several features based on nuclei properties and density in the patches of size 64 × 64 used to compute the clustering (Supplementary Fig. 5). Nuclei with their centroids within 20 px (that is, 6.5 μm) of an already segmented nucleus were discarded. We also implemented a test time augmentation scheme^46^ to correct the segmentation on low-contrast patches by dividing the slides into two groups, depending on the correction needed, and took the minimum between the predictions from increased contrast versions from the original image. We finally exported the segmentation to QuPath 0.4.0 (ref. ^40^) to extract shape and intensity features (such as area, perimeter, circularity and DAB intensity within 30-μm diameter circular tile) and removed the remaining artifacts by enforcing a threshold on the perimeter and circularity of the predicted nuclei. We ultimately averaged the resulting values over the nuclei detected in the patches of size 64 × 64 used to compute the clustering—that is, whose centroid is located the patch—to get one value by marker for each patch and counted the number of nuclei detected in each patch to construct a cell density measure.

The association between the confirmed objective response and clusters was assessed through statistical analysis based on each cluster’s relative percentage in each slide. We used a Mann–Whitney *U*-test to assess if there was a statistically significant difference between the percentage of each cluster and the confirmed response to T-DXd. All *P* values were adjusted for multiple hypothesis testing using the Benjamini–Hochberg method. Finally, two pathologists (I.J.G. and M.L.T.) reviewed the cell phenotype in cluster 6. The same protocol followed for cohort 1 was used to train a new model in cohort 2 (*n* = 65; Extended Data Fig. 5 and Supplementary Fig. 6). ROIs were not annotated in HER2 pathology slides from cohort 2.

### RT–PCR

Tumor samples obtained at baseline from cohort 3 (HER2 IHC 0) were qualified for RT–PCR if the sample contained ≥30% tumor cells (*n* = 24). Then, 1 µg of RNA was reversed into cDNA with SuperScript Vilo cDNA Synthesis Kit (Thermo Fisher Scientific). Quantitative PCR was performed with TaqMan Fast Advanced Master Mix using TaqMan Gene Expression Assays, Hs01111580 (HER2), Hs00197427 (*ACTB*) and HS99999901 (*18S*), as recommended by the supplier (Thermo Fisher Scientific). *18S* and *ACTB* were used as internal references to normalize input cDNA. The comparative threshold (Δ*C*_t_) method was used to quantify *ERBB2* expression. The median of *ERBB2* relative expression was 30. The confirmed ORRs were assessed according to *ERBB2* expression levels (< or > median expression). Association with efficacy was done with the confirmed objective response.

### Genomic analyses

The tumor samples were qualified for WES if the sample contained ≥30% tumor cells. In total, 89 frozen tumor biopsies at baseline (38 cohort 1, 37 cohort 2, 14 cohort 3) and 21 (5 cohort 1, 11 cohort 2, 5 cohort 3) at resistance were analyzed. Eighty-four blood samples were used as germline control. Genomic DNA was isolated from biopsy and blood of patients using the QIAamp DNA Mini Kit and DNeasy Blood and Tissue Kit (Qiagen), respectively, according to the manufacturer’s guidelines. DNA concentration was measured using QubitTM dsDNA Broad Range Assay (Invitrogen). A quantity of 30–100 ng of DNA was used for preparing the WES libraries. For the WES, the DNA was sheared with the Covaris E220 system (LGC Genomics/KBioscience). SureSelect Low Input Target Enrichment was used. In brief, DNA fragments were end-repaired, extended with an ‘A’ base on the 3′ end, ligated with paired-end adaptors with the Bravo Platform (Agilent Technologies) and amplified to generate libraries (10 cycles). Hybridization-based exome enrichment was performed using the Agilent SureSelect Low Input Clinical Research Exome V2 target enrichment system (Agilent Technologies). The final libraries were indexed, pooled and sequenced using the onboard cluster method, as paired-end sequencing (2 × 100-bp reads) on an Illumina NovaSeq 6000 sequencer at Gustave Roussy.

Statistical analyses of association with efficacy were done with the confirmed objective response.

### Bioinformatic analyses

Point mutations, small indels and CNAs were detected using an end-to-end pipeline described previously^47^. In brief, paired-end reads were controlled (FastQC version 0.11.8), trimmed (fastp version 0.20) and aligned to the reference human genome GRCh37 (BWA-MEM version 0.7). Aligned reads were processed following the best practices of GATK bundle version 4.1.8.1. Processed reads were then used as input to mutation-calling and CNA-calling algorithms. As advised in GATK guidelines, we used a panel-of-normal to remove artifactual or false-positive mutations recurrently found in normal blood samples from patients with cancer treated at Gustave Roussy. Point mutations and small indels were called using Mutect2 (ref. ^48^) and the panel-of-normal. All putative variants identified by Mutect2 were first filtered to account for possible sample contamination and read orientation artifacts. Additional threshold-based and rule-based filtering was applied to the read coverage, genomic position and variant allele frequency (VAF). Specific rules were applied to tumor samples from patients with WES at baseline and progression (11 patients). More particularly, for each baseline (and progression, respectively) sample, SAMtools version 1.9 mpileup^49^ was run on the positions where Mutect2 identified and retained mutations in the corresponding progression (and baseline, respectively) sample to rule out incorrect claims of mutation acquisition or loss caused by conservative filtering or non-detection by Mutect2. If a mutation detected by Mutect2 in a sample at a given timepoint was also seen in the sample from the other timepoint with sufficiently many reads supporting the alternative allele (at least one read if coverage <100, two reads if 100 ≤ coverage < 500 and three reads if coverage >500), the mutation was also called in the latter sample. Additionally, in patients without a matched blood sample, any mutation identified as germline at any of the two timepoints was discarded from both samples. After all the filtering, 20,469 somatic point mutations and small indels were considered in the analysis of the 110 WES samples (89 at baseline, 21 at resistance; Supplementary Fig. 7).

CNAs, tumor purity and average tumor ploidy were identified with the FACETS R package version 0.5.14 (ref. ^50^) run with parameters cval_pre = 25 and cval_pro = 500. To mitigate the effect of segmentation errors, only gene CNAs arising from segments spanning fewer than 10 Mb were considered in downstream analyses. Additionally, in an effort to increase the sensitivity of CNA calling on driver genes, a second run of FACETS with parameters cval_pre = 25 and cval_pro = 150 was performed and used to replace the copy number estimations on driver genes only if the second-run segment was smaller than 3 Mb in size or three times smaller than the first-run segment. Each CNA was classified into one of six categories^47^, and only high-level focal amplifications (medium-level also considered for oncogenes) or homozygous focal deletions were considered. Oncogenic events were identified by intersecting point mutations, small indels, gene amplifications and gene deletions with the OncoKB database (December 2022 release, https://github.com/oncokb/oncokb-annotator)^51^.

Patient and sample attributes for the TCGA cohort were downloaded from the GDC data portal (gdc-tcga-phs000178-controlled) using the R package GenomicDataCommons version 1.18.0 and from the supplementary tables publicly available on the PanCanAtlas page (https://gdc.cancer.gov/about-data/publications/pancanatlas). Only patients included in the BRCA study of TCGA and for which we found no reason for exclusion were considered (Supplementary Fig. 8). Point mutations and small indels for TCGA samples were downloaded with permission from the file mc3.v.0.2.8.CONTROLLED.maf.gz (https://gdc.cancer.gov/about-data/publications/mc3-2017)^52^ and filtered as follows. All point mutations seen by at least two callers among the five used by MC3 (ref. ^52^) and all small indels seen by INDELOCATOR or VARSCANI were selected. These putative variants were then filtered using the same threshold-based and rule-based filtering as used on DAISY samples. This filtering procedure was carefully determined to maximize the alignment between our internal pipeline and the MC3 pipeline^47^. WES BAM files for TCGA BRCA patients were downloaded with permission and processed with FACETS using the same pipeline as used for DAISY samples.

### In vitro experiments

MCF-7 and SK-BR-3 cells were purchased from the German Collection of Microorganisms and Cell Cultures. MCF-7 cells were grown in DMEM (Gibco) supplemented with 1% GlutaMAX (Gibco) and SK-BR-3 in McCoy’s 5A medium (Gibco) in standard incubation conditions at 37 °C with 5% CO_2_. Both media were supplemented with 10% FBS, penicillin (100 U ml^−1^) and streptomycin (100 µg ml^−1^) and cells. All cell lines were kept as mycoplasma-free. Cells were seeded at 5 × 10^3^ cells per well in a 96-well plate. Twenty-four hours later, cells were transfected with the siRNAs targeting *SLX4* gene (ON-TARGETplus siRNA, SMARTpool, Dharmacon) or Non-targeting Control Pool (Dharmacon) according to the manufacturer’s instructions. To evaluate cell viability, DXd was added 48 h after siRNA transfection with eight-point dose–response titrations in triplicate (0.1−1,000 nM) for 5 days. Cell viability was examined using the CellTiter-Glo Luminescent Cell Viability Assay (Promega) using a VICTOR Nivo multimode plate reader (PerkinElmer). Survival at each drug concentration was calculated as a percentage relative to the corresponding untreated control. To assess *SLX4* expression, total RNAs were extracted using RNeasy Mini Kit (Qiagen) and reversed into cDNA with Maxima Reverse Transcriptase (Thermo Fisher Scientific). Quantitative PCR was performed with Master Mix PCR Power SYBR Green (Life Technologies) using CFX96 Real Time System (Bio-Rad). The specific primers for *SLX4* used in this study were 5′-GTGAAGGTCGGAGTCAACG-3′ and 5′-GGTGAAGACGCCAGTGGACTC-3′. GAPDH was used as an internal reference to normalize input cDNA. The ΔCt method was used. Data were expressed as mean ± s.e.m. for *n* = 3. For viability assay, significance was analyzed by Welch’s *t*-test (two-tailed). Statistical analysis was performed using GraphPad Prism 9 (GraphPad Software). *P* values less than 0.05 were considered statistically significant

### Statistical analyses

The primary endpoint was the confirmed ORR evaluated by investigator assessment using RECIST 1.1. The secondary endpoints included duration of response, PFS, OS and clinical benefit rate evaluated on the FAS and per cohort. Safety was evaluated on the safety population and per cohort. The required number of assessable patients for cohort 1 (*n* = 67) and cohort 2 (*n* = 40) was determined using the A’Hern design with the following hypothesis: cohort 1 (p0 = 30%; p1 = 45%; α = 5%; power = 80%) and cohort 2 (p0 = 20%; p1 = 40%; α = 5%; power = 85%). The regimen would be declared promising in cohort 1 if 27 patients present a confirmed objective response among 67 and in cohort 2 if 13 confirmed objective responses were observed among 40. The required number of assessable patients for cohort 3 (*n* = 40) was designed using an optimal two-stage design^53^ (α = 5%, power = 85%) with non-progression at 3 months as short-term endpoint (p20 = 30%, p21 = 50%) and confirmed objective response as primary endpoint (p10 = 20%, p11 = 40%). A stop for non-promising activity was planned to be declared if four patients or fewer among the first 16 present non-progressive disease at 3 months. At final analysis of cohort 3, the regimen would be defined as promising if 13 patients or more present a confirmed objective response among 40. In cohort 3, recruitment was stopped after 40 patients (37 assessable for activity) because of slow recruitment. For each cohort, it was assumed a rate of 10% non-evaluable patients, and sample size was increased: cohort 1: *n* = 74, cohort 2: *n* = 44, cohort 3: *n* = 44. Full details are provided in the Statistical Analysis Plan.

The primary endpoint was reported for each cohort, and comparisons were considered exploratory. Comparison between cohorts was performed using the chi-square test or Fisher’s exact test for qualitative variables and the Kruskal–Wallis test for continuous variables; and multivariable analysis was performed using logistic regression model adjusted for hormone receptor status of primary tumor (hormone receptor-positive versus hormone receptor-negative), time from initial diagnosis to metastatic disease (0–3 months versus >3 months), time from diagnosis of metastatic disease to inclusion (0–24 months versus 24–60 months versus >60 months), number of metastatic sites (<3 versus ≥3 sites), presence of liver metastases (yes versus no) and ECOG performance status (0 versus 1) at inclusion. ORs were estimated with corresponding 95% CI. The best tumor shrinkage of target lesions was plotted on a waterfall plot and compared between cohorts using the Kruskal–Wallis test. Time to event endpoints (PFS and duration of response) were estimated using the Kaplan–Meier method, and comparison between groups was performed using the log-rank test. Multivariable analysis was performed using Cox proportional hazards model adjusted for the same variables used for the confirmed objective response. HRs were estimated with corresponding 95% CI. In the exploratory objective of modulation of tumor microenvironment, comparisons of each biomarker at baseline and on-treatment were performed using the Wilcoxon matched-pairs signed-rank test. Cell distance analysis *P* values were adjusted for multiple hypothesis testing using the Benjamini–Hochberg method. All statistical tests were two-sided. Statistical analyses were performed using STATA software version 16 (StataCorp).

### Reporting summary

Further information on research design is available in the Nature Portfolio Reporting Summary linked to this article.

## Online content

Any methods, additional references, Nature Portfolio reporting summaries, source data, extended data, supplementary information, acknowledgements, peer review information; details of author contributions and competing interests; and statements of data and code availability are available at 10.1038/s41591-023-02478-2.

### Supplementary information

Supplementary Information Supplementary Table 1 and Supplementary Figs. 1–8

Reporting Summary

Supplementary Data 1

Supplementary Data 2

## Figures and Tables

**Extended Data Table 1:**
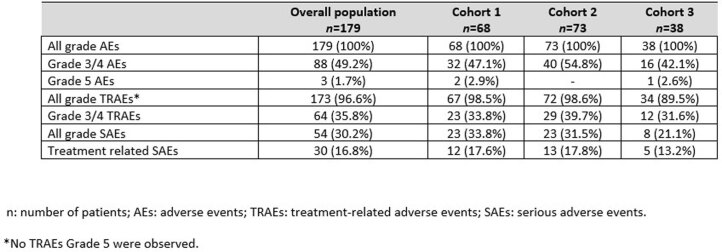
Number (%) of patients who developed adverse effects in the safety population

**Extended Data Table 2:**
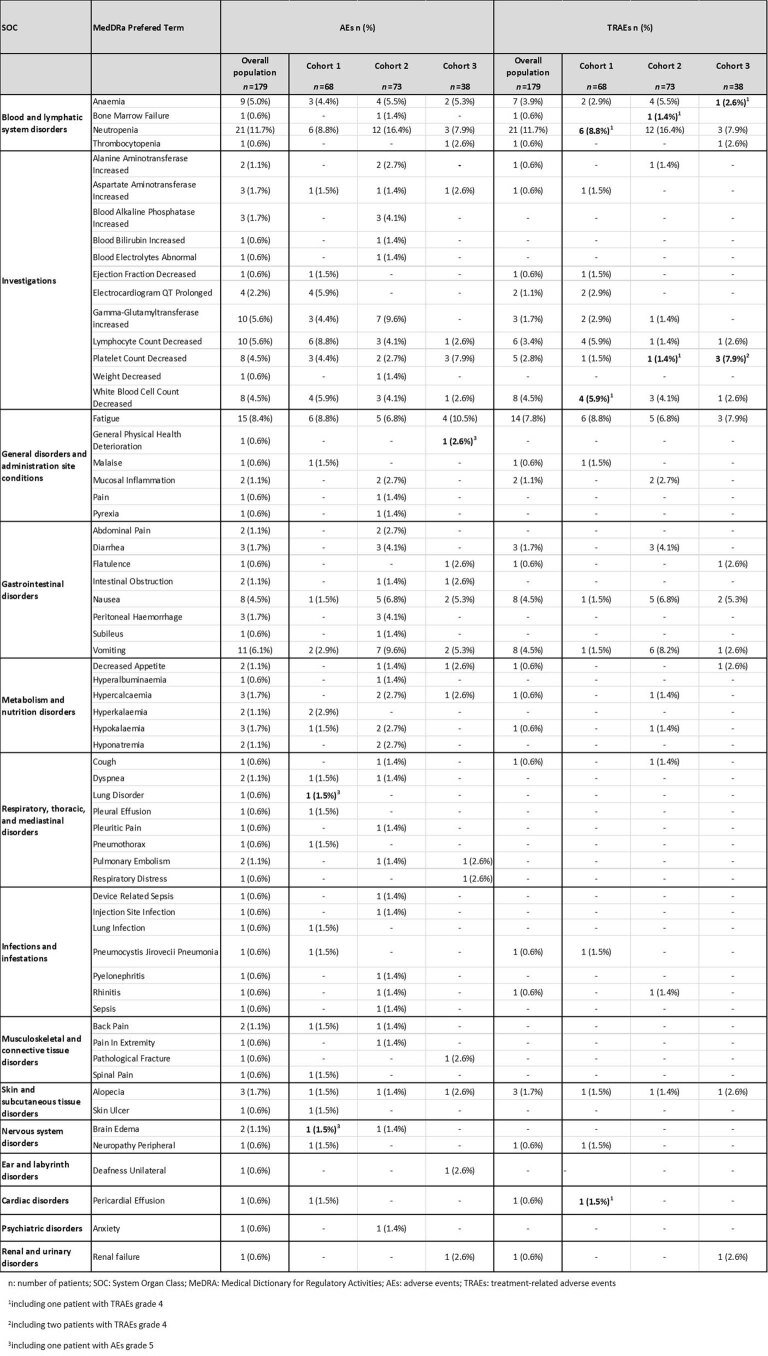
Number (%) of patients who experienced adverse effects and treatment-related adverse effects of grade > 2 in the safety population

## Data Availability

All data used in the present study are available within the manuscript and its Supplementary Information files. Clinical data are available for access upon external requests. Applicants should contact the following email address ‘mariafernanda.mosele@gustaveroussy.fr’ to request access to clinical data. The request will be discussed internally in the joint steering committee of the study. The decision will be communicated within 1 month from the request. Applicants must complete specific documents to be granted a user license. Whole-exome sequencing data generated in this study have been deposited to the European Genome-phenome Archive (EGA) under accession number EGAD00001011110. Refer to the forms and README file from https://github.com/gustaveroussy/DAISY_Public/tree/master/data for instructions on how to access the data. Other data that support the findings of this study are available from the corresponding author upon reasonable request. Databases used in the study include gnomAD (https://gnomad.broadinstitute.org), OncoKB Precision Oncology Knowledge Base (https://www.oncokb.org), Clinical Interpretation of Variants in Cancer (https://civicdb.org) and dbNSFP version 4.1.a (https://sites.google.com/site/jpopgen/dbNSFP).

## Code availability

The source code to reproduce the analyses presented in this paper is available at https://github.com/gustaveroussy/DAISY_Public.
