# Supplementary material for: Trastuzumab deruxtecan in metastatic breast cancer with variable HER2 expression: the phase 2 DAISY trial
Source: Nat Med. 2023 Jul 24;29(8):2110–20. doi: 10.1038/s41591-023-02478-2 (PMC10427426; doi:10.1038/s41591-023-02478-2)
Supplement: Supplementary file 1 — Supplementary Table 1 and Supplementary Figs. 1–8 [file 41591_2023_2478_MOESM1_ESM.pdf]

nature medicine

Article

https://doi.org/10.1038/s41591-023-02478-2

Trastuzumab deruxtecan in metastatic breast cancer with variable HER2 expression: the phase 2 DAISY trial

In the format provided by the authors and unedited

Supplementary Tables

Supplementary Table 1. Cell characterization of Cluster 6

**Cell characteristics (*n*=60)***	**Mean (%)**	**95% Confidence Interval (CI)**
Cellularity	30	25-34
Fibroblasts	56	50-62
Immune cells	27	22-32
Tumor cells	7	3-11
Other cells	9	6-13
**Tumor cells (*n*=28)**		
HER2 IHC 0	40	24-56
HER2 IHC 1+	48	33-63
HER2 IHC 2+	12	0-23

*60 out of 61 slides analyzed by machine learning were interpretable for Cluster 6

Supplementary Figures

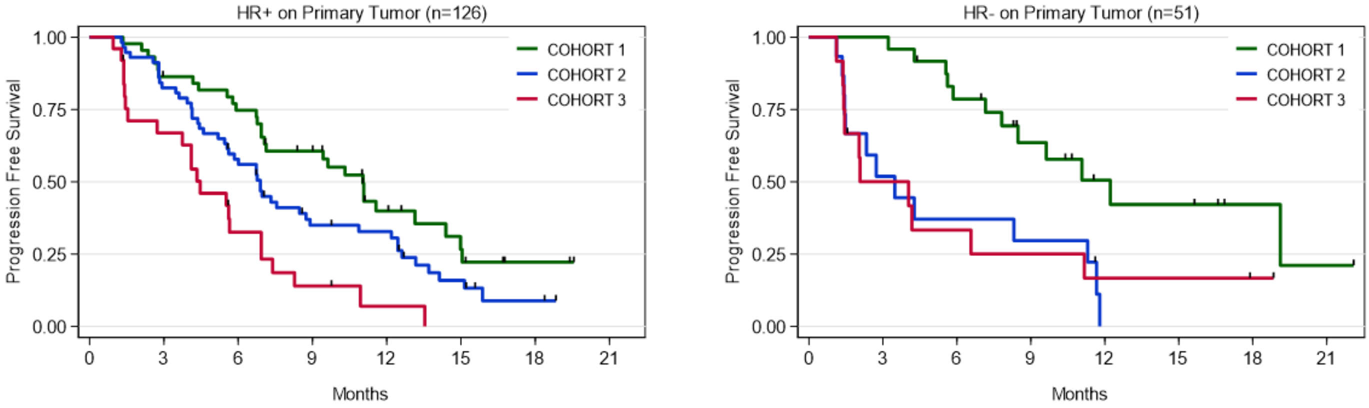

	0	3	6	9	12	15	18	21
COHORT 1	44	37	32	23	12	6	2	0
COHORT 2	57	47	32	17	15	6	2	0
COHORT 3	25	16	7	3	1	0	0	0

	0	3	6	9	12	15	18	21
COHORT 1	24	24	18	11	6	5	2	1
COHORT 2	15	7	5	4	0	0	0	0
COHORT 3	12	6	4	3	2	2	1	0

Supplementary Fig. 1. Kaplan-Meier plot of PFS per cohort according to HR-expression in the FAS population (*n*=177). The median PFS in the HR-positive BC subgroup was 11 months (95% CI 6.9-14.4) in cohort 1, 6.9 months (95% CI 5.5-8.7) in cohort 2, and 4.5 months (95% CI 1.5-6.9) in cohort 3; in the HR-negative subgroup the median PFS was 12.2 months (95% CI 7.8-NR) in cohort 1, 3.5 months (95% CI 1.4-11.3) in cohort 2, and 2.1 months (95% CI 1.4-11.2) in cohort 3.

PFS: progression-free survival; HR: hormone receptor; FAS: Full Analysis Set; BC: breast cancer CI: confidence interval; NR: not reached.

a

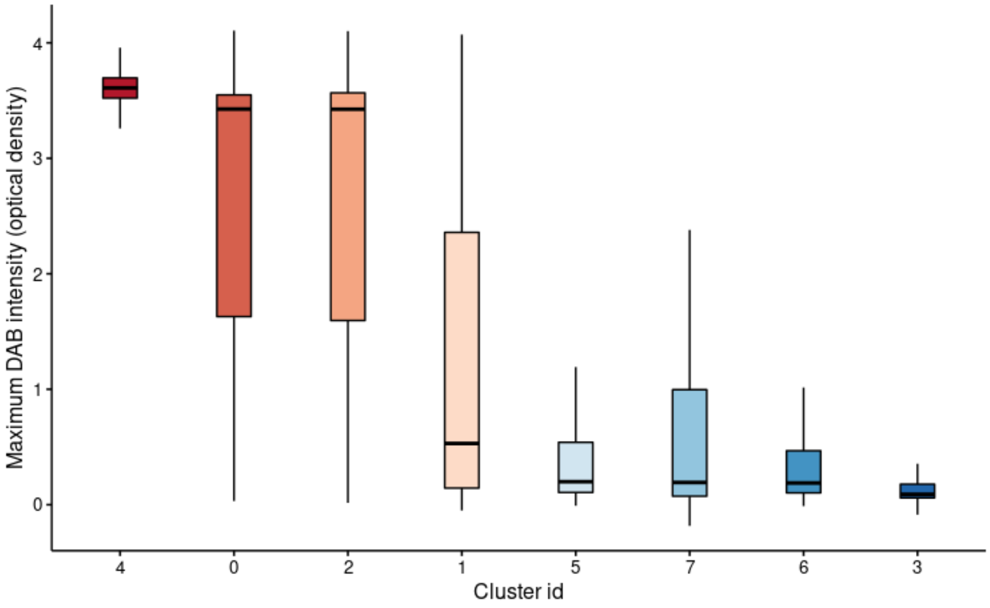

b

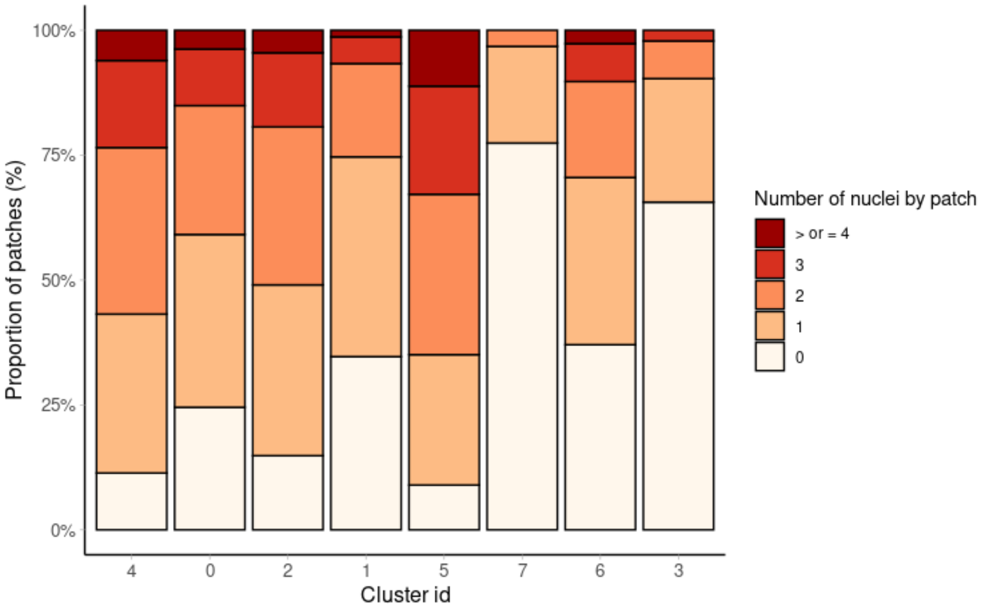

Supplementary Fig. 2. HER2 expression patterns and treatment response. a. Distribution of DAB intensity (HER2 expression) within each cluster in HER2 pathology slides from cohort 1 (HER2 overexpressing, *n*=61). The boxplot represents the distribution of maximum DAB intensity in the patches of 64x64 px used to compute the clustering. Box center lines, box ranges, and whiskers indicate medians, quartiles, and 1.5× the IQR, respectively. The values were computed by taking the average of the maximum DAB intensity measured in a circular area of 30 μm of diameter around each nuclei detected in each patch. The intensity was based on an optical density measurement using the software Qu Path and the detection relied on an unsupervised nuclei segmentation algorithm. Patches without nuclei were discarded. The clusters are arranged by DAB intensity median decreasing order. **b. Representation of cell density within each cluster in HER2 pathology slides from cohort 1 (HER2 overexpressing, *n*=61).** The bar plot represents the proportion of patches with different number of nuclei per patch within each cluster. The resulting values were computed by counting the number of nuclei detected within each patch of 64 X 64 px used to compute the clustering. The detection relied on an unsupervised nuclei segmentation algorithm. The clusters are arranged by DAB intensity median decreasing order.

HER2: HER2: Human Epidermal growth factor Receptor 2; DAB: diaminobenzidine; IQR: inter-quartile range.

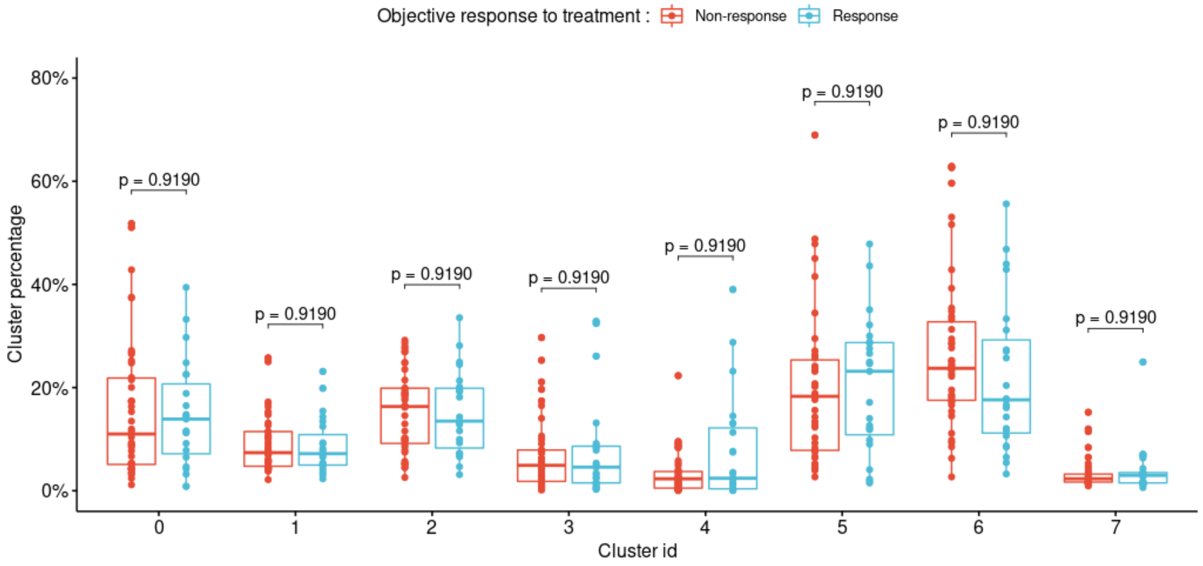

Supplementary Fig. 3. HER2 expression patterns and treatment response. Clusters' relative percentage according to sensitivity to T-DXd in cohort 2 obtained with the model pre trained in cohort 1. For each patient, the corresponding HER2 pathology slide (*n*=65) was divided into 64 × 64 px non-overlapping patches that were classified into 8 clusters using a Mini-Batch K-Means algorithm trained on cohort 1 data. The following box plot illustrates the relative percentage of each cluster in each slide and its association with the confirmed objective response and non-response to T-DXd. Box center lines, box ranges, whiskers and dots indicate medians, quartiles, 1.5× the IQR and outliers, respectively. No cluster presented a significant association with T-DXd response. P-values were calculated using Mann-Whitney U test and adjusted for multiple hypothesis testing using the Benjamini-Hochberg method. All statistical tests were two sided.

T-DXd: Trastuzumab Deruxtecan; HER2: HER2: Human Epidermal growth factor Receptor 2.

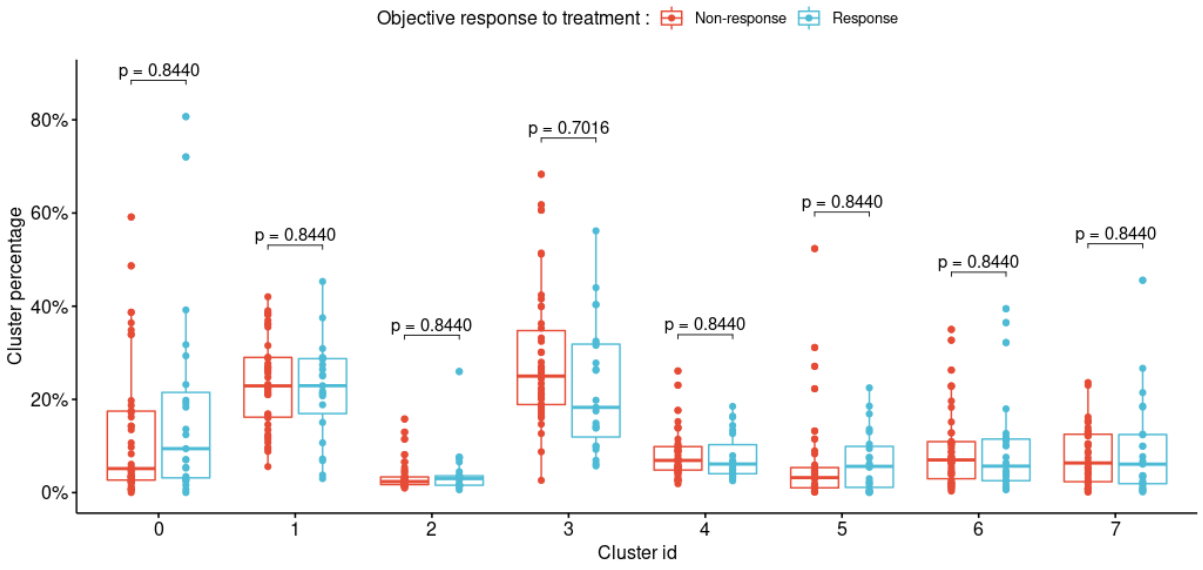

Supplementary Fig. 4. HER2 expression patterns and treatment response. Clusters' relative percentage according to sensitivity to T-DXd in cohort 2 obtained with the model trained in cohort 2. For each patient, the corresponding HER2 pathology slide (n=65) was divided into 64×64 px non-overlapping patches that were classified into 8 clusters using a Mini-Batch K-Means algorithm trained on cohort 2 data. The following box plot illustrates the relative percentage of each cluster in each slide and its association with the confirmed objective response and non-response to T-DXd. Box center lines, box ranges, whiskers and dots indicate medians, quartiles, 1.5× the IQR and outliers, respectively. No cluster presented a significant association with T-DXd response. P-values were calculated using Mann-Whitney U test and adjusted for multiple hypothesis testing using the Benjamini-Hochberg method. All statistical tests were two sided.

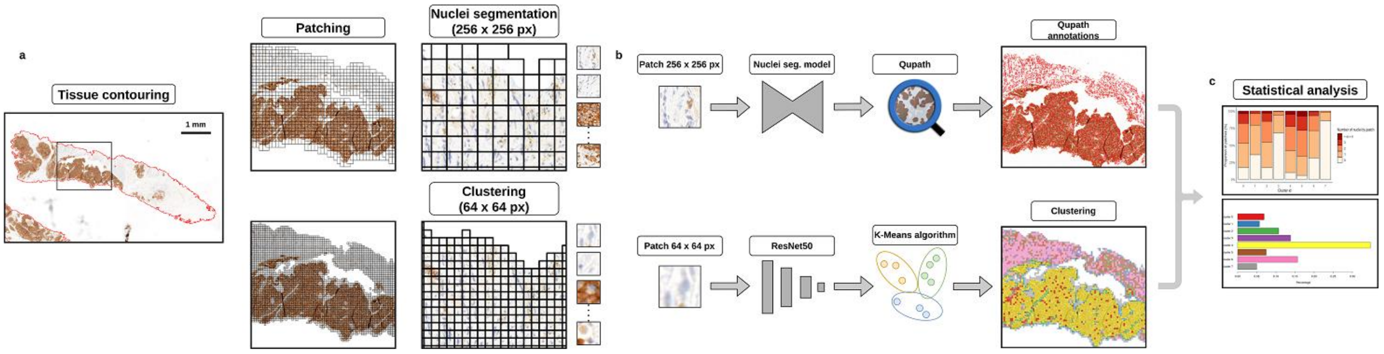

Supplementary Fig. 5. Pipeline overview for HER2 spatial analysis. a. Illustration of the pre-processing method on one sample. Contouring of ROIs was performed using morphological operations and simple annotations. Then, patches of size of 256×256 px with 50% overlap were extracted for nuclei segmentation and patches of size of 64×64 px with no overlap were extracted to perform the clustering. **b. Nuclei segmentation and clustering pipeline.** The patches of size 256×256 px were provided to the segmentation model and the resulting annotations were then exported to Qupath to compute final markers. Visual appearance descriptors were computed from the 64×64 px patches using a ResNet50 model truncated after the 3rd residual block pre-trained on ImageNet dividing each slide into 8 clusters using the Mini-Batch K-Means algorithm. **c. Results of the analysis on one sample.** The relative percentage of each cluster was computed by counting the number of patches belonging to the cluster over the total number of patches per slide. To interpret clustering decision, the markers from the nuclei segmentation were averaged across the 64×64 px patches used to compute the clustering to get one value by patch for every marker. The proportion of patches for different number of nuclei and relative percentage of clusters is represented.

ROI: regions of interested; HER2: Human Epidermal growth factor Receptor 2

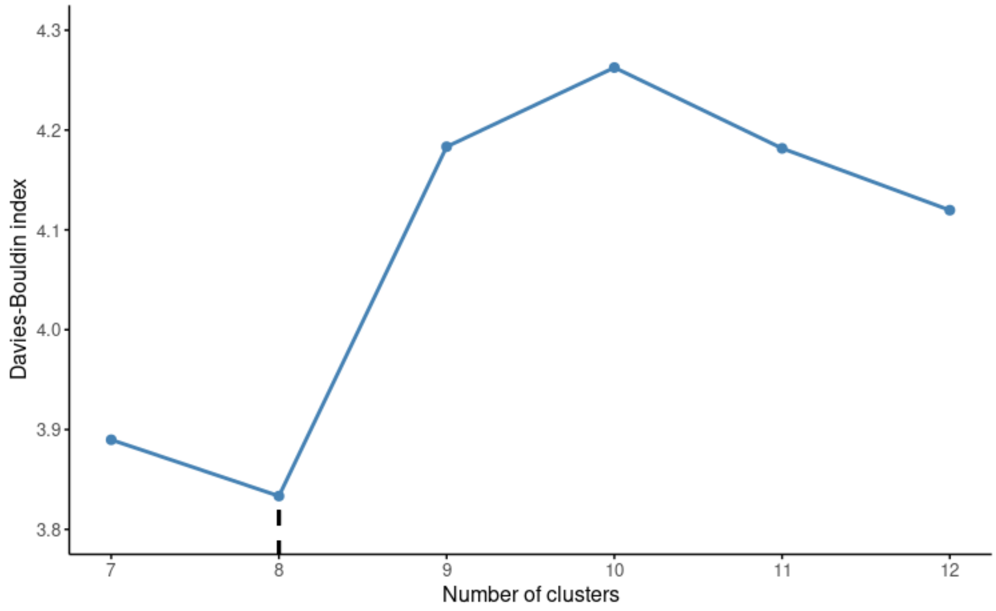

Supplementary Fig. 6. Identifying an optimal number of clusters in cohort 2. The Davies-Bouldin index was computed from Mini-Batch K-Means clustering using a number of clusters ranging from 7 to 12. This index represents how the clusters are similar to each other, with a lower value pointing toward a better segmentation. Minimum *is* highlighted on the graph at 8 clusters.

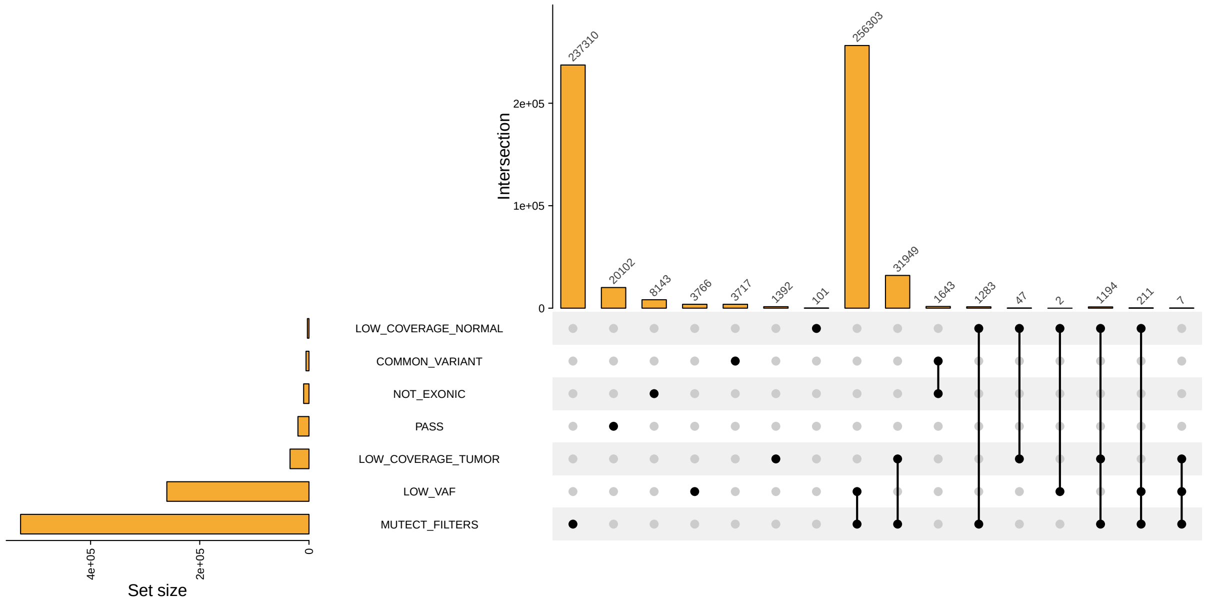

Supplementary Fig. 7. Mutations filtering on DAISY WES samples. Mutations were identified using Mutect2 and further filtered using different quality criteria. This upset plot summarizes the effect of each filtering criteria individually and in combination with other filters**.** Mutations that passed all filters are described in the “PASS” set.

WES: whole exome sequencing.

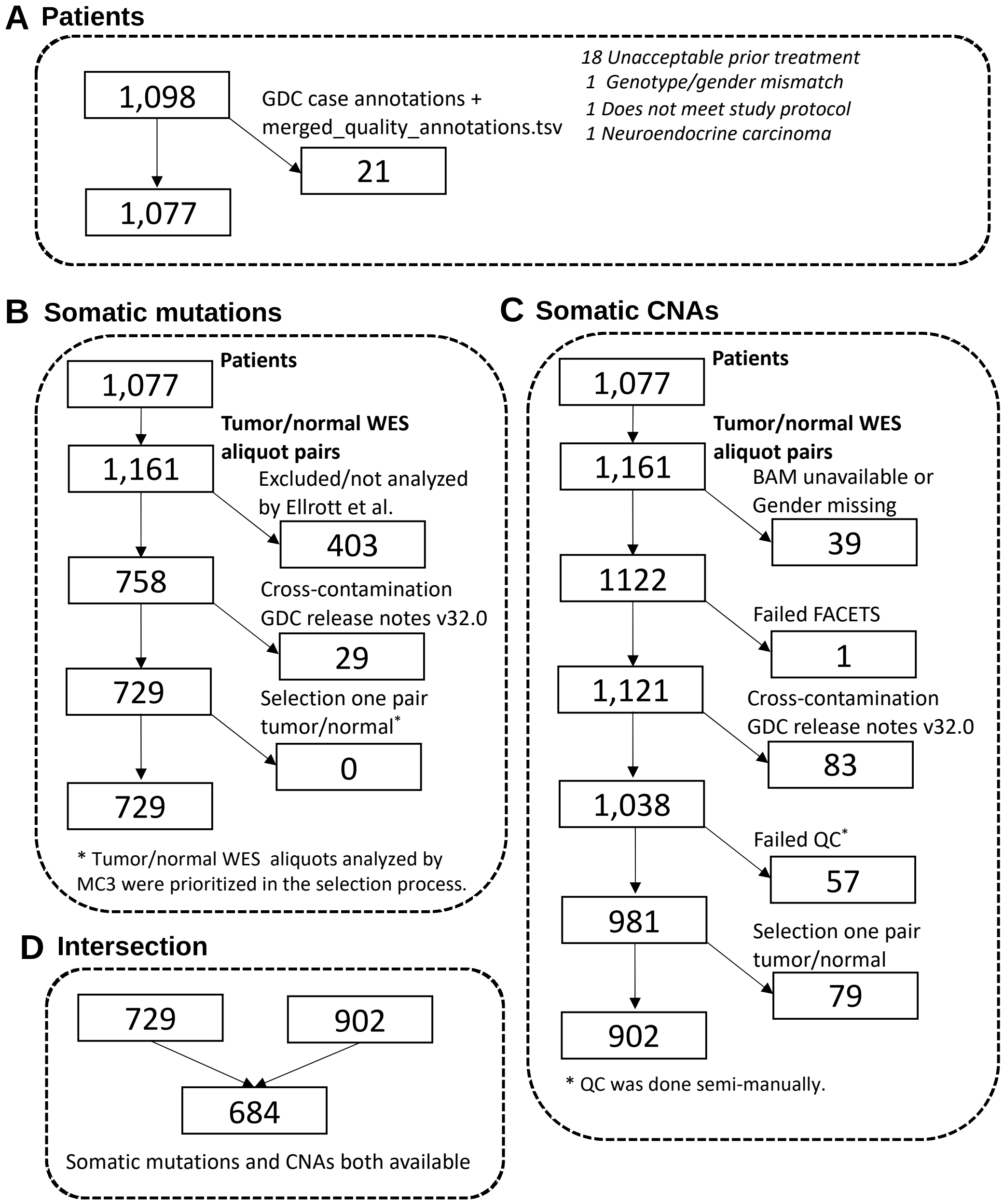

Supplementary Fig. 8. TCGA patients and samples. a. Hierarchical diagram showing how the TCGA breast cancer patients considered in our study were selected. **b. c.** Identical to a. but considering somatic mutations and somatic CNAs, respectively. **d.** Intersection of mutation and CNA data.

TGCA: The Cancer Genome Atlas Program; CNAs: copy number alterations.
