# Supplementary material for: Trastuzumab deruxtecan in metastatic breast cancer with variable HER2 expression: the phase 2 DAISY trial
Source: Nat Med. 2023 Jul 24;29(8):2110–20. doi: 10.1038/s41591-023-02478-2 (PMC10427426; doi:10.1038/s41591-023-02478-2)
Supplement: Supplementary file 2 — Reporting Summary [file 41591_2023_2478_MOESM2_ESM.pdf]

nature portfolio

Corresponding author(s): Fabrice André

Last updated by author(s): 27/06/2023

Reporting Summary

Nature Portfolio wishes to improve the reproducibility of the work that we publish. This form provides structure for consistency and transparency in reporting. For further information on Nature Portfolio policies, see our Editorial Policies and the Editorial Policy Checklist.

Statistics

For all statistical analyses, confirm that the following items are present in the figure legend, table legend, main text, or Methods section.

n/a	Confirmed	
☐	☒	The exact sample size (*n*) for each experimental group/condition, given as a discrete number and unit of measurement
☐	☒	A statement on whether measurements were taken from distinct samples or whether the same sample was measured repeatedly
☐	☒	The statistical test(s) used AND whether they are one- or two-sided<br>*Only common tests should be described solely by name; describe more complex techniques in the Methods section.*
☐	☒	A description of all covariates tested
☐	☒	A description of any assumptions or corrections, such as tests of normality and adjustment for multiple comparisons
☐	☐	A full description of the statistical parameters including central tendency (e.g. means) or other basic estimates (e.g. regression coefficient) AND variation (e.g. standard deviation) or associated estimates of uncertainty (e.g. confidence intervals)
☐	☒	For null hypothesis testing, the test statistic (e.g. *F*, *t*, *r*) with confidence intervals, effect sizes, degrees of freedom and *P* value noted<br>*Give P values as exact values whenever suitable.*
☒	☐	For Bayesian analysis, information on the choice of priors and Markov chain Monte Carlo settings
☒	☐	For hierarchical and complex designs, identification of the appropriate level for tests and full reporting of outcomes
☐	☒	Estimates of effect sizes (e.g. Cohen's *d*, Pearson's *r*), indicating how they were calculated

Our web collection on statistics for biologists contains articles on many of the points above.

Software and code

Policy information about availability of computer code

Data collection	Patients and samples attributes for the TCGA breast cancer study were downloaded from the GDC data portal using the R package GenomicDataCommons version 1.18.0.<br>Data collection was performed with CSOnline modul from Ennov Clinical.
Data analysis	WES alignment: Raw sequence data were aligned to the human genome (NCBI build 37) using BWA version 0.7.17. Base qualities were recalibrated using BaseRecalibrator and ApplyBQSR tools from GATK bundle v4.1.8.1.<br>WES quality-control: Raw sequence data quality was controlled using FastQC v0.11.8. Sequencing reads were processed by fastp v0.20 prior to alignment. Alignment quality was controlled using SAMtools v1.9, and GATK v4.1.8.1.<br>SNVs and indels were called using Mutect2 from GATK v4.1.8.1. CNAs were identified using FACETS R package v0.5.14.<br>SNVs and indels were filtered using GATK v4.1.8.1 filtering procedure from the best practices.<br>For MIF analysis, the WSI were analyzed with QuPath 0.3.2.<br>Analysis of the slides stained for HER2 expression were performed using Python version 3.9, Openslide version 3.4.1, Pytorch version 1.9.0, and Scikit-Learn version 0.24.2. Nuclei features were computed on QuPath version 0.4.0.<br>The source to reproduce the analyses supporting the analyses presented in the paper is available at https://github.com/gustaveroussy/DAISY_Public.<br>Statistical analyses were carried out using Stata software v16 (StataCorp, Texas, US).<br>For in vitro experiments, statistical analysis was performed using GraphPad Prism 9 (GraphPad Software).

For manuscripts utilizing custom algorithms or software that are central to the research but not yet described in published literature, software must be made available to editors and reviewers. We strongly encourage code deposition in a community repository (e.g. GitHub). See the Nature Portfolio guidelines for submitting code & software for further information.

Data

Policy information about availability of data

All manuscripts must include a data availability statement. This statement should provide the following information, where applicable:

- Accession codes, unique identifiers, or web links for publicly available datasets
- A description of any restrictions on data availability
- For clinical datasets or third party data, please ensure that the statement adheres to our policy

All data used in the present study are available within the manuscript and its supplementary information files.
Clinical data is available for access upon external requests. Applicants should contact the following email address "mariafernanda.mosele@gustaveroussy.fr" to request access to clinical data. The request will be discussed internally in the joint steering committee of the study. The decision will be communicate within one month from the request. Applicants must complete specific documents in order to be granted a user license.
WES data generated in this study have been deposited to the European Genome-Phenome Archive (EGA) under the accession number EGAD00001011110. Please refer to the forms and README file from
https://github.com/gustaveroussy/DAISY_Public/tree/master/data
for instructions on how to access the data. Other data that support the findings of this study are available from the corresponding author upon request.

SNVs and indels for TCGA breast cancer samples were downloaded with permission from the file mc3.v.0.2.8.CONTROLLED.maf.gz (https://gdc.cancer.gov/about-data/publications/mc3-2017). Raw WES data of TCGA breast cancer samples were downloaded with permission from the GDC-controlled Google Cloud bucket gs://gdc-tcga-phs000178-controlled.

Databases used in the study include gnomAD (https://gnomad.broadinstitute.org), OncoKB Precision Oncology Knowledge Base (https://www.oncokb.org), Clinical Interpretation of Variants in Cancer (https://civicdb.org), dbNSFP version 4.1.a (https://sites.google.com/site/jpopgen/dbNSFP).

Research involving human participants, their data, or biological material

Policy information about studies with human participants or human data. See also policy information about sex, gender (identity/presentation), and sexual orientation and race, ethnicity and racism.

Reporting on sex and gender	Findings from DAISY trial apply to both sexes. Sex was determined based on self-reporting. Gender was not considered in study design.
Reporting on race, ethnicity, or other socially relevant groupings	Race or ethniccity analyses was not performed in DAISY trial since it is forbidden in France.
Population characteristics	Patients presented metastatic breast cancer with any hormone receptor and HER2 status. For cancers, with expression of hormone receptors, the patients were included if they presented resistance to endocrine therapy and CDK4/6 inhibitors. For patients with HER2-overexpressing breast cancer they must have progressed on Trastuzumab and TDM1. Patients must have received at least one line of chemotherapy in the metastatic setting. Patients must have received at least one line of chemotherapy in the metastatic setting. In the DAISY trial, 186 patients were enrolled and 179 were included in the safety popuation. The median age of the population is 55 years. 99.4% of the patientes are women and 0.6% men. At baseline, 43% of patients presented a PS0 and 71.5% presented hormone receptor positive breast cancer. 64.8% of patients presented 3 or more metastatic sites at baseline and 57.6% of patients presented liver metastasis. 53% of patients received 5 or more lines of previous therapies in the metastatic setting. The median interval from initial diagnosis to metastatic disease was 25.8 months and the median interval from metastatic diagnosis to inclusion was 43.7 months.
Recruitment	186 patients meeting the clinical trial eligibility criteria were recruited during clinical consultation with oncologists. The first patient was enrolled on November 4th 2019 and the last one on March 3rd 2021 in 15 study centers in France. We did not identify self-selection bias or other bias that could impact the results.
Ethics oversight	All patients who entered in DAISY trial signed an informed consent. DAISY trial was approved by the French ethics committee, CPP – Ile de France on September 05th 2019 and the French health authorities, ANSM, on July 08th 2019.

Note that full information on the approval of the study protocol must also be provided in the manuscript.

Field-specific reporting

Please select the one below that is the best fit for your research. If you are not sure, read the appropriate sections before making your selection.

☒ Life sciences ☐ Behavioural & social sciences ☐ Ecological, evolutionary & environmental sciences

For a reference copy of the document with all sections, see nature.com/documents/nr-reporting-summary-flat.pdf

Life sciences study design

All studies must disclose on these points even when the disclosure is negative.

Sample size	The primary endpoint was the confirmed objective response rate evaluated in each cohort, defined as the presence of a confirmed partial or complete response assessed by investigators. The investigator evaluated the objective response using RECIST v1.1 criteria. The required number of assessable patients for cohort 1 (n=67) and 2 (n=40) was determined using the A'Hern design with the following hypothesis: Cohort 1 (p0 = 30%; p1 = 45%, alpha = 5%, 1-beta = 80%) and Cohort 2 (p0 = 20%; p1 = 40%, alpha = 5%, power= 85%). The regimen would be declared promising in cohort 1 if 27 patients present a confirmed objective response among 67 and in cohort 2 if 13 confirmed objective response were observed among 40. Cohort 3 was designed using an optimal two stage design (alpha = 5%, power= 85%) with non-progression at 3 months as short-term endpoint (p20=30% and p21=50%) and confirmed objective response as primary endpoint (p10=20%, p11=40%). A stop for non-promising activity was planned to be declared if 4 patients or less among the first 16 present non-progressive disease at 3 months. At final analysis of cohort 3, the regimen would be defined as promising if 13 patients or more present a confirmed objective response among 40. In cohort 3, recruitment was stopped after 40 patients (37 assessable for activity) because slow recruitment. For each cohort, it was assume a rate of 10% non-evaluable patients and sample size was increased: Cohort 1: n=74, Cohort 2: n=44, Cohort 3: n=44. Full details are provided in statistical analysis plan.
Data exclusions	Of the 186 patients included in DAISY trial, 7 patients who did not receive at least one dose of T-DXd were excluded from the safety population and 2 additional patients who did not have a valid first post-baseline assessment of disease status or who did not have progressive disease were excluded from the Full Analysis Set population as planned in the statistical analysis plan. Annex 3 includes all analyses that have been done by the statistician.<br>For HER2 spatial sitribution analyses, 7 patients (cohort 1) and 7 patients (cohort 2) were excluded because HER2 slides were not exploitable or could not be digitalized with the appropriate scanner.<br>For T-Dxd distribution on-treatment, 3 out of 10 pairs of biopsies were not analyzable because of absence of cancer cells.<br>For the RT-PCR tumor samples with <30% of tumor cells were excluded.<br>For WES analyses, tumor samples with <30% of tumor cells were excluded.
Replication	Since the paper reports a prospective clinical trial, there is no attemp to replicate the finding in the same paper. Nevertheless, the primary objective of the study was predefined and thus limits the risk of non replication.
Randomization	The trial was a phase II ,single arm, not randomized trial. Patients were allocated into the different cohorts according to HER2 level expression.
Blinding	Investigators were not blinded regarding cohort assignment. Blinding was not considered relevant since the oncologist knew the HER2 status of the patient.

Reporting for specific materials, systems and methods

We require information from authors about some types of materials, experimental systems and methods used in many studies. Here, indicate whether each material, system or method listed is relevant to your study. If you are not sure if a list item applies to your research, read the appropriate section before selecting a response.

Materials & experimental systems

n/a	Involved in the study	
☐	☒	Antibodies
☐	☒	Eukaryotic cell lines
☒	☐	Palaeontology and archaeology
☒	☐	Animals and other organisms
☐	☒	Clinical data
☒	☐	Dual use research of concern
☒	☐	Plants

Methods

n/a	Involved in the study	
☒	☐	ChIP-seq
☒	☐	Flow cytometry
☒	☐	MRI-based neuroimaging

Antibodies

Antibodies used	The antibody used for HER2 staining was a rabbit monoclonal primary antibody supplied by Roche, clone 4B5, reference 05999570001, pre-diluted ready to use.<br>For the enhanced HER2 protocol, OptiView DAB IHC Detection Kit (Roche Diagnostics) was used instead of ultraView Universal DAB Detection Kit (Roche Diagnostics).<br>Staining for DXd-IgG was performed using a primary antibody against DXd (antiXAFG5737-1A3-ocChimera, Daiichi Sankyo) with Leica BOND RX automated slide stainer (Leica Biosystems). Rabbit isotype control antibody (#PA0777, Leica Biosystems) was used as negative reagent control.<br>MIF was performed with the ULTIVUE kit, reference ULT30801, containing the Immuno8 FixVUE panel containing eight pre-diluted antibodies according to manufacturer instructions ready to use. The panel of antibodies included: anti CD3 (clone BC33), anti CD4 (clone SP35), anti CD8 (clone C8/144B), anti CD68 (clone KP-1), anti FoxP3 (clone 236A/E7), anti PD-1 (clone CAL20), anti PD-L1 (clone 73-10), anti PanCK/SOX10 (clone AE1/AE3/BC34).
Validation	Each primary antibody is tested and optimized for ISP and has as reference the corresponding DAB. Expert pathologist assess quality of ISP staining (eg sensitivity, specificity, staining pattern, signal/background ratio) in multiple indications including tissue positive

controls and tumor types as compared to serial sections stained with classical DAB IHC. Primary antibodies developed for ISP are already validated for FFPE applications.
Immuno8 FixVUE panel has also been validated as a panel by performing precision testing and reproducibility in several studies. You can refer to our website for the Immuno8 FixVUE panel validation.
The specificity of the anti-DXd antibody was confirmed by the fact that DXd staining disappeared in IHC when the antibody was preincubated with DXd, while it did not disappear when preincubated with SN-38. For more information refer to https://doi.org/10.1158/1078-0432.CCR-21-0397.

Eukaryotic cell lines

Policy information about cell lines and Sex and Gender in Research

Cell line source(s)	MCF-7 and SK-BR-3 cells were purchased from DSMZ (Germany). These cell lines are derived from two female patients respectively with breast cancer.
Authentication	None of these cell lines were authenticated.
Mycoplasma contamination	The cell lines were not tested for mycoplasma contamination.
Commonly misidentified lines (See ICLAC register)	The cell lines used in the study are cell lines commonly used.

Clinical data

Policy information about clinical studies

All manuscripts should comply with the ICMJE guidelines for publication of clinical research and a completed CONSORT checklist must be included with all submissions.

Clinical trial registration	NCT04132960
Study protocol	Annexes 1 and 2 were submitted with the first submission of the DAISY trial
Data collection	The trial included patients from November 2019 to March 2021. Data were captured by clinical research assistants in the centers that participated to the trial using a eCRF. Data was collected with CSOnline from Ennov Clinical. Trial monitoring was done on a regular basis by the sponsor UNICANCER.
Outcomes	The primary endpoint was the confirmed objective response rate evaluated in each cohort, defined as the presence of a confirmed partial or complete response assessed by investigators. The investigator evaluated the objective response using RECIST v1.1 criteria. For the cohort 3 (IHC0+), a short term primary endpoint is used for the interim analysis. The short term primary endpoint was the rate of patient without progression at 3 months. The investigator evaluated the progression using RECIST v1.1.<br>The secondary endpoints were predifined by the study steering committee. The secondary endpoints included progression-free survival (PFS), duration of response (DOR), overall survival (OS), and clinical benefit rate (CBR) and were evaluated ion the Full Analysis Set (FAS) and per cohort.<br>PFS was defined as the time from inclusion until progression or death from any cause. At the time of analysis, patients alive and without progression were censored at the date of the last tumor assessment. The investigator evaluated progression using RECIST v1.1.<br>DOR was applicable to subject with objective response, either complete response (CR) or partial response (PR), and was defined as the time from the first documented CR or PR until progression or death from any cause. The objective response and progression were evaluated by invetsigator using RECIST v1.1.<br>OS was defined as the time from inclusion to death from any cause. Patients still alive at the time of analysis were censored at the date of last follow up.<br>CBR was defined as the presence of at least a CR or PR or stable disease > 6 months assessed by investigators using RECIST v1.1.<br>More details are available in the statistical analysis plan.
