# Supplementary material for: Trastuzumab deruxtecan in metastatic breast cancer with variable HER2 expression: the phase 2 DAISY trial
Source: Nat Med. 2023 Jul 24;29(8):2110–20. doi: 10.1038/s41591-023-02478-2 (PMC10427426; doi:10.1038/s41591-023-02478-2)
Supplement: Supplementary file 4 — Supplementary Data 2 [file 41591_2023_2478_MOESM4_ESM.pdf]

Personalized Medicine / UCBG

Protocol n°: UC-0105/1815

EudraCT n°: 2018-004868-57

Phase 2, Open label Study of DS-8201a, an Anti-HER2-AntIbody Drug Conjugate (ADC) for advanced BreaSt Cancer patients, with biomarkers analysis to characterize response/resistance to therapY

DAISY

Version n°3.0 – January 25, 2021

PROTOCOL	VERSION N° - DATE	CPP approval	ANSM authorization
INITIAL	V1.1 – 12 JULY 2019	September 05, 2019	July 08, 2019
AMENDMENT N°4	V2.0 – 31 JANUARY 2020	May 26, 2020	April 01, 2020
AMENDMENT N°7	V3.0 – 25 JANUARY 2021		

**COORDINATING INVESTIGATOR**	Dr Véronique DIERAS<br>Centre Eugène Marquis<br>Rennes - France<br>Phone: +33 (0)2 99 25 32 80<br>Fax: +33 (0)2 99 25 32 33<br>Email: v.dieras@rennes.unicancer.fr

**SPONSOR**	**UNICANCER**<br>101, rue de Tolbiac<br>75654 Paris Cedex 13 (France)<br>Phone: + 33 (0)1 44 23 04 04 – Fax: + 33 (0)1 44 23 55 69

CONTACT DETAILS
PROTOCOL N°: UC-0105/1815

Trial Title:

Phase 2, Open label Study of DS-8201a, an Anti-HER2-Antibody Drug Conjugate (ADC) for advanced Breast Cancer patients, with biomarkers analysis to characterize response/resistance to therapy

**WRITING COMMITTEE**	Fabrice André, Véronique Diéras, Thomas Filleron, Magali Lacroix-Triki, Lilian Amrein, Marta Jimenez, Céline Mahier Ait-Oukhatar.

**CONTACT DETAILS AND USEFUL INFORMATION**	
**Dr Véronique DIERAS**<br>**Coordinating Investigator**	Centre Eugène Marquis<br>Rennes - France<br>Phone: +33 (0)2 99 25 32 80<br>Fax: +33 (0)2 99 25 32 33<br>Email: v.dieras@rennes.unicancer.fr
**Dr Elise DELUCHE**<br>**Co-coordinating Investigator**	Hôpital DUPUYTREN – CHU de Limoges<br>Limoges -France<br>Phone: +33 (0)5 55 05 61 00<br>Email: Elise.DELUCHE@chu-limoges.fr
**Prof Fabrice ANDRE**<br>**Ancillary/translational coordinator**	Gustave Roussy<br>Villejuif-France<br>Phone: +33 (0)1 42 11 43 71<br>Email: fabrice.andre@gustaveroussy.fr
**Mr Thomas FILLERON**<br>**Statistician**	Institut Claudius Regaud, IUCT-O<br>Toulouse -France<br>Phone: +33 (0)5 31 15 58 65<br>Email: Filleron.Thomas@iuct-oncopole.fr
**Mrs Céline MAHIER AIT-OUKHATAR**<br>**Project Manager**	R&D UNICANCER<br>101 rue de Tolbiac – 75654 PARIS Cedex 13<br>Phone: +33 (0)1 44 23 55 84<br>Email: c-mahier@unicancer.fr

**Pharmacovigilance Notification**	R&D UNICANCER Pharmacovigilance Unit<br>Phone: + 33 (0)1 44 23 04 16<br>Fax: + 33 (0)1 44 23 55 70<br>Email: pv-rd@unicancer.fr
**Magali Lacroix-Triki Pathologist, coordinator of the GEFPICS' group**	Gustave Roussy<br>Villejuif-France<br>Phone: +33 (0)1 42 11 44 55<br>Email: magali.lacroix-triki@gustaveroussy.fr

APPROVAL AND SIGNATURE
FOR PROTOCOL N°: UC-0105/1815

Trial Title: Phase 2, Open label Study of DS-8201a, an Anti-HER2-Antibody Drug Conjugate (ADC) for advanced Breast Cancer patients, with biomarkers analysis to characterize response/resistance to therapy

Title	Name	Address	Date (dd-Mmm-yyyy)	Signature
**Director of Clinical Operations**	**Beata Juzyna**	R&D UNICANCER<br>101 rue de Tolbiac – 75654 PARIS Cedex 13<br>Phone: +33 (0) 6 11 44 59 47<br>Email: b-juzyna@unicancer.fr	Fait le 04/02/2021	Beata JUZYNA<br>Signé par Beata JUZYNA<br>Signé et certifié par yousign
**Project Manager**	**Céline Mahier Aït-Oukhatar**	R&D UNICANCER<br>101 rue de Tolbiac – 75654 PARIS Cedex 13<br>Phone: +33 (0)1 44 23 55 84<br>Email: c-mahier@unicancer.fr	Fait le 04/02/2021	Céline Mahier Ait-Oukhatar<br>Signé par Céline Mahier Ait-Oukhatar<br>Signé et certifié par yousign
**Coordinating Investigator**	**Véronique Diéras**	Centre Eugène Marquis<br>Rennes - France<br>Phone: +33 (0)2 99 25 32 80<br>Email: v.dieras@rennes.unicancer.fr	Fait le 08/02/2021	Véronique DIERAS<br>Signé par Véronique DIERAS<br>Signé et certifié par yousign
**Biostatistician**	**Thomas Filleron**	Institut Claudius Regaud, IUCT-O<br>Toulouse -France<br>Phone: +33 (0)5 31 15 58 65<br>Email: Filleron.Thomas@iuct-oncopole.fr	Fait le 04/02/2021	Thomas FILLERON<br>Signé par Thomas FILLERON<br>Signé et certifié par yousign

TABLE OF CONTENT

TABLE OF CONTENT 5

LIST OF ABBREVIATIONS 9

STATEMENT OF COMPLIANCE 12

PROTOCOL SUMMARY 13

1. INTRODUCTION 30

1.1. Background information 30

1.1.1. Disease/pathology epidemiology/prognosis 30

1.1.2. Disease current treatment 30

1.1.3. Overview of DS-8201a, the investigational medicinal products (IMP) 31

1.2. Trial rationale 33

1.3. Justification for the therapeutic regimens and treatment durations 34

1.4. Potential risks and benefits 35

1.4.1. Known potential risks 35

1.4.2. Known potential benefits 35

1.5. Trial population 35

2. TRIAL OBJECTIVES 36

2.1. Primary objective 36

2.2. Secondary objective(s) 36

3. TRIAL DESIGN AND ENDPOINTS 36

3.1. Description of the trial Design 36

3.2. Trial Endpoints....37

3.2.1. Primary endpoint 37

3.2.2. Secondary endpoint(s) 37

3.3. Progression of the trial 38

3.4. Inclusion procedure 38

3.5. Premature Trial Terminations and Suspension....39

3.6. Patient's trial withdrawal and discontinuation....39

4. PATIENT SELECTION 39

4.1. Diagnosis and inclusion criteria....39

4.2. Non-inclusion criteria 40

5. TRIAL TREATMENTS/INTERVENTIONS 43

5.1. Description of trial treatments/interventions 43
5.2. Acquisition, reception, and storage 43
5.3. Preparation 43
5.4. Administration 43
5.5. Trial treatments accountability, return and destruction 44
5.6. Formulation, appearance, packaging, and labelling 44
5.7. Dose adaptation 44
5.8. Patients' discontinuations of treatment 45
5.9. Concomitant medications and therapies 46
5.9.1. Authorized concomitant treatments 46
5.9.2. Prohibited concomitant treatments 46
5.9.3. Rescue medications and therapies/Treatment at disease progression 47
5.9.4. Contraception during the trial 47

6. EVALUATION OF TREATMENT AND SAFETY 49

6.1. Efficacy evaluation 49
6.2. Safety evaluation 50
6.2.1. ECG guidelines 50
6.2.2. LVEF guidelines 50
6.3. Centralized review 50
6.3.1. Radiological review 50
6.3.2. HER2 status review 50

7. DESCRIPTION OF VISITS AND INVESTIGATIONS 51

7.1. Baseline visit 51
7.1.1. Within 28 days before treatment initiation 51
7.1.2. Within 14 days before treatment initiation 52
7.2. Visits and assessment during treatment period 53
7.3. End-of-treatment visit (6 weeks after last DS-8201a administration) 54
7.4. Follow-up (every 12 weeks and up to 3 years) 55
7.5. Provisions in case of treatment or trial interruption 56

8. REPORTING OF ADVERSE EVENTS 56

8.1. Adverse event: general definition 56

8.2. Serious adverse event: general definition 56
8.3. Adverse Event of Special Interest (AESI) 58
8.3.1. Combined Elevations of Aminotransferases and Bilirubin ***Erreur ! Signet non défini.***
8.3.2. Cardiac Related Events including Prolonged QTc Interval and LVEF *58*
8.3.3. Interstitial Lung Disease / Pneumonitis *58*
8.3.4. Infusion-related Reactions ***Erreur ! Signet non défini.***
8.4. Measures to be taken in case of a serious adverse event 59

9. TRANSLATIONAL STUDY 60

10. DESCRIPTION OF STATISTICAL METHODS 61
10.1. Statistical hypothesis and sample size determination 61
10.1.1. Cohort 1: HER2 IHC3+ or IHC2+/ISH+ *62*
10.1.2. Cohort 2 HER2 IHC1+ or IHC2+/ISH- *62*
10.1.3. Cohort 3: HER2 IHC0+ *63*
10.2. Trial populations to be analyzed 64
10.3. Planned statistical analysis 64
10.3.1. Demographic data *64*
10.3.2. Primary endpoint *64*
10.3.3. Secondary endpoint *64*
10.3.4. Modifications of SAP and initial statistical analysis strategy *65*

11. OVERSIGHT COMMITTEES 65
11.1. Independent Data Monitoring Committee (IDMC) 65
11.2. Steering Executive Committee 66

12. QUALITY ASSURANCE 67
12.1. Data collection 67
12.2. Access to data 68
12.3. Study monitoring 68
12.4. Audits and inspections 69

13. ETHICAL AND REGULATORY CONSIDERATIONS 69
13.1. General requirements 69
13.2. Patient identification 70
13.3. Patient information and consent 70
13.4. Insurance compensation 71
13.5. Investigator responsibilities 71

13.6. Federation of the Patient Committees for Clinical Research in Cancerology 72
13.7. Human biological samples collection 72
13.7.1. Storage and use of disease assessment samples (blood, biopsy, tumor specimen, etc.) 72
13.7.2. Collecting additional biological samples for research purpose 72

14. DATA PROCESSING AND CONSERVATION OF DOCUMENTS AND DATA OF THE RESEARCH 73

14.1. Data processing....73
14.1.1. Under the responsibility of the sponsor 73
14.1.2. In the investigational center, when computerized medical records are used 73
14.2. Retention of documents by investigator sites....74

15. DATA OWNERSHIP AND CONFIDENTIALITY 75

16. PUBLICATION RULES 75

17. REFERENCES 77

18. APPENDICES 79

Appendix 1. Performance status evaluation – WHO scale....80
Appendix 2. World Medical Association - Declaration of Helsinki 81
Appendix 3. ICH Harmonized Tripartite Guideline for Good Clinical Practice (ICH-GCP)....82
Appendix 4. A brief summary of tumor classification RECIST v1.1....83
Appendix 5. Toxicity criteria (NCI-CTCAE)....88
Appendix 6. Translational study....89
Appendix 7. Dose modification for DS-8201a....94
Appendix 8. Actions required in cases of increases in liver biochemistry and evaluation of Hy's Law....102

LIST OF ABBREVIATIONS

ADC	Antibody drug conjugate
AE	Adverse event
AESI	Adverse event of special interest
ANSM	Agence nationale de sécurité du médicament et des produits de santé
ALT (SGPT)	Alanine aminotransferase
ALP	Alkaline phosphatase
ANC	Absolute neutrophil count
APTT	Activated partial thromboplastin time
AST (SGOT)	Aspartate aminotransferase
BRC	Biological Resource Centre
CBR	Clinical benefit rate
CDK	Cyclin-dependent kinase
CNS	Central nervous system
CPP	Comité de protection des personnes
CR	Complete response
CRF	Case report form
CRA	Clinical research associate
CRO	Contract research organization
CT	Computed tomography
CTC	Circulating tumor cells
CTCAE	Common terminology criteria for adverse events
CtDNA	Circulating tumor deoxyribonucleic acid
CYP	Cytochrome P450
DLCO	Diffusing capacity of the lungs for carbon monoxide
DOR	Duration of response
EC	Ethic committee
ECG	Electrocardiogram
ECHO	Echocardiogram
ECOG	Eastern cooperative oncology group

eCRF	Electronic case report form
eg	example
EMA	European medicines agency
EOT	End of treatment
FAS	Full analysis set
FDA	Food and drug administration
FFPE	Formalin-fixed paraffin-embedded
g	Gram
GCP	Good clinical practice
GEFPICS	Groupe d'Etude des Facteurs Pronostiques par Immunohistochimie dans les cancers du sein
HER2	Human epidermal growth factor receptor 2
HIV	Human Immunodeficiency Virus
HR	Hazard ratio
Hb	Hemoglobin
IB	Investigator brochure
ICH	International conference on harmonization
ICF	Informed consent form
IDMC	Independent data monitoring committee
IF	Immunofluorescence
IHC	Immunohistochemistry
ILD	Interstitial lung disease
IMF	Investigator master file
IMF-P	Investigator master file-pharmacy
INN	International non-proprietary name
INR	International normalized ratio
IP	Investigational products
ISH	In situ hybridization
ITT	Intent-to-treat
IV	Intravenous
L	liter
LDH	Lactate dehydrogenase
LVEF	Left ventricular ejection fraction

mg	milligram(s)
min	minute(s)
mL	milliliter(s)
MRI	Magnetic resonance imaging scan
MUGA	multigated acquisition
NCI	National cancer institute
nmol	nanomole
NSAID	Nonsteroidal Anti-inflammatory Drug
OATP	Organic anion transporting polypeptide
OS	Overall survival
PD	Progressive disease
PIS	Patient information sheet
PR	Partial response
PFS	Progression-free survival
QTc	Corrected QT interval
RECIST	Response evaluation criteria in solid tumors
SAE	Serious adverse event
SAP	Statistical analysis plan
SD	Stable disease
SPC	Summary of product characteristics
SpO2	Organic anion transporting polypeptide
TMF	Trial Master File
uSAE	Unexpected serious adverse event
ULN	Upper limit of normal
WBC	White blood cell
WES	Whole exome sequencing
WHO	World health organization

STATEMENT OF COMPLIANCE

UNICANCER, the trial sponsor, certifies that the trial DAISY will be conducted in compliance with the protocol described in this document, and in accordance with the current Declaration of Helsinki, the current ICH Harmonised Tripartite Guideline for Good Clinical Practice (ICH-GCP) and for Good Manufacturing Practice (ICH-GMP), the European Directive 2001/20/CE on the conduct of clinical studies and subsequent texts (Eudralex Vol 10), Regulation (EU) 2016/679 (General Data Protection Regulation) and the national legal requirements.

PROTOCOL SUMMARY

SYNOPSIS

A) TRIAL IDENTIFICATION	
SPONSOR – PROTOCOL CODE NUMBER: UC -0105/1815	
VERSION (NUMBER & DATE): VERSION 3.0, JANUARY 25, 2021	
TRIAL TITLE: PHASE 2, OPEN LABEL STUDY OF DS-8201a, AN ANTI-HER2-ANTIBODY DRUG CONJUGATE (ADC) FOR ADVANCED BREAST CANCER PATIENTS, WITH BIOMARKERS ANALYSIS TO CHARACTERIZE RESPONSE/RESISTANCE TO THERAPY<br>PHASE (FOR TRIALS ON MEDICINAL PRODUCTS): 2	
TRIAL TITLE FOR LAY PEOPLE:<br>CLINICAL STUDY ASSESSING A NEW ANTIBODY FOR ALL PATIENTS WITH ADVANCED BREAST CANCER, WITH BIOLOGICAL ANALYSIS TO CHARACTERIZE MARKERS OF EFFICACY OR NON-EFFICACY TO THERAPY	
ABBREVIATED TITLE: DAISY	
COORDINATING INVESTIGATOR: VERONIQUE DIERAS<br>CO-COORDINATING INVESTIGATOR : ELISE DELUCHE<br>TRANSLATIONAL,COORDINATING INVESTIGATOR: FABRICE ANDRE / MAGALI LACROIX-TRIKI	
NUMBER OF CENTERS: 20	NUMBER OF PATIENTS: 192

B) SPONSOR IDENTIFICATION	
NAME:	**UNICANCER**<br>101, rue de Tolbiac<br>75654 Paris Cedex 13<br>(France)
CONTACT PERSON:	**CELINE MAHIER AIT OUKHATAR**<br>Project Manager<br>**R&D UNICANCER**<br>Phone: +33 (0)1 44 23 55 84<br>Email: c-mahier@unicancer.fr

C) TRIAL GENERAL INFORMATION

INDICATION:

Adult patients with advanced breast cancer after at least a first line of chemotherapy in metastatic setting:

-if HER2 IHC3+ or IHC2+/ISH+ resistant to trastuzumab and TDM-1

-if HER2 IHC0+ or IHC1+ or IHC2+/ISH- HR+ resistant to endocrine therapy.

TRIAL DESCRIPTION/DESIGN:

Multicenter, open-label phase II trial assessing the efficacy of DS-8201a monotherapy in patients with metastatic breast cancer.

PRIMARY OBJECTIVE:

The main objective is to evaluate the anti-tumor activity of DS-8201a in three cohorts of advanced breast cancer patients:

- a cohort of HER2 over-expressing (HER2 IHC3+ or HER2 IHC2+/ISH+)
- a cohort HER2 low-expressing (IHC1+ or IHC2+/ISH-)
- a cohort HER2 non-expressing (IHC0+)

SECONDARY OBJECTIVES:

- To evaluate the efficacy in each cohort, in term of:
  - ➢ Best objective response as assessed by central review
  - ➢ Progression Free Survival (PFS)
  - ➢ Duration of response (DOR)
  - ➢ Clinical benefit rate (CBR)
  - ➢ Overall survival (OS)
- To evaluate the safety of DS-8201a overall and per cohort by NCI-CTCAE v5.0

TRANSLATIONAL OBJECTIVE:

- Exploration of bystander effect of DS-8201a in human samples
- Description of immune effects of DS-8201a
- Develop predictors of primary resistance or outcome
- Identify mechanisms of secondary resistance

DIAGNOSIS AND INCLUSION CRITERIA:

Patients must meet all of the following criteria to be included in the study:

1. Patient must have signed a written informed consent form prior to any study specific procedures. When the patient is physically unable to give his/her written consent, a trusted person of his/her choice, independent from the investigator or the sponsor, can confirm in writing the patient's consent.
2. Female or male subjects aged ≥18 years.
3. Patient with histologically-confirmed diagnosis of invasive breast cancer. Tumors can be either HER2 IHC3+/ISH positive or IHC2+/ISH positive or IHC2+/ISH negative or IHC1+ or IHC0+, on most recent tumor tissue sample available.
4. Patient with a documented radiologic metastatic progression.
5. Patient considered by the investigator as not amenable to any other validated therapeutic option, after at least 1 line of chemotherapy in metastatic setting:
    - All patients with HER2 over-expressing (IHC3+ and IHC2+/ISH+) and hormone receptor positive or negative tumor must have been treated with taxanes and have progressed on trastuzumab and on TDM-1. Prior treatment with pertuzumab is not required.
    - Patient with HER2 negative (IHC0, 1+, and 2+/ISH-) tumor must have been treated with anthracyclines and taxanes. Patients with hormone receptor positive (ER+ and/or PR+) tumor must be resistant to endocrine therapy and CDK4/6 inhibitors.
6. Non-bone metastatic site easily accessible to biopsy.
7. Presence of at least one radiologically measurable lesion according to RECIST v1.1.
8. Patient with WHO performance status ≤1.
9. Adequate bone marrow function: absolute neutrophil count (ANC) ≥1.5 × 10^9/L, platelet count ≥100 × 10^9/L, and hemoglobin ≥9 g/dL (transfusion is not allowed within 1 week prior to baseline assessment).
10. Adequate liver function: total bilirubin level ≤1.5 × the upper limit of normal (ULN) range if no liver metastases or <3 x ULN in the presence of documented Gilbert's Syndrome or liver metastases. AST and ALT levels ≤3 × ULN (AST and ALT≤ 5 ULN when documented liver metastasis).
11. Adequate blood clotting function: International Normalized Ratio (INR)/Prothrombin Time (PT) and either partial thromboplastin Time (PTT) or activated Partial Thromboplastin Time (aPTT) ≤1.5 x ULN.
12. Adequate renal function: estimated creatinine clearance ≥30 mL/min according to the Cockcroft-Gault formula or serum creatinine ≤1.5 x ULN.

13. Adequate cardiac function: left ventricular ejection fraction (LVEF) ≥ 50% at baseline as determined by either echocardiogram (ECHO) or multigated acquisition (MUGA) scan within 28 days before inclusion.
14. Male and female subjects of reproductive/childbearing potential must agree to use of a highly effective contraception for subjects throughout the study and for at least 7 months for females and 4 months for males after last study treatment administration if the risk of conception exists.
15. Women of childbearing potential must have a negative serum pregnancy test within 14 days of enrolment or urine pregnancy test 72 hours prior to enrolment.
16. Patient is willing to comply with 2 sequential tumor biopsies (baseline and at first progression), and with a series of blood samples throughout the study.
17. Patients must be affiliated to a Social Security System.

NON-INCLUSION CRITERIA:

1. Patient with a breast cancer amenable for resection or radiation therapy with curative intent.
2. Patient has spinal cord compression or clinically active central nervous system metastases, defined as untreated and symptomatic, or requiring therapy with corticosteroids or anticonvulsants to control associated symptoms. Subjects with clinically inactive brain metastases may be included in the study. Subjects with treated brain metastases that are no longer symptomatic and who require no treatment with corticosteroids or anticonvulsants may be included in the study if they have recovered from the acute toxic effect of radiotherapy.
3. Patient with bone metastatic disease only.
4. Patient with multiple primary malignancies within 3 years, except adequately resected non-melanoma skin cancer, curatively treated in-situ disease, other solid tumors curatively treated, or contralateral breast cancer.
5. Persistent unresolved toxicities with grade ≥2 (except alopecia and renal function). Subject with chronic Grade 2 toxicities may be eligible per the discretion of the Investigator after consultation with the Sponsor (eg, Grade 2 chemotherapy-induced neuropathy).
6. Patient receiving treatments such as anti-cancer chemotherapy (non-antibody based immunotherapy, retinoid therapy, hormonal therapy) within 3 weeks before inclusion (within 2 weeks or 5 half-lives, whichever is longer, for small-molecule targeted agents, within 6 weeks for nitrosureas or mitomycin C), anti-body-based anti-cancer therapy within 4 weeks before inclusion, radiotherapy including palliative stereotactic radiation to chest within 4 weeks (palliative stereotactic radiotherapy to others areas: within 2 weeks), chloroquine/hydroxychloroquine within 14 days or major surgery within 4 weeks. Participation in other studies involving investigational drug(s) within 4 weeks prior to study entry and/or during study participation.

7. *Deleted Criterion (Amendment N°4)*
8. Patients with a concomitant use of chronic systemic (IV or oral) corticosteroids or other immunosuppressive medications except for managing adverse events (inhaled steroids or intra articular steroid injections are permitted in this study.) Subjects with bronchopulmonary disorders who require intermittent use of bronchodilators (such as albuterol) will not be excluded from this study.
9. Patient with history of (noninfectious) interstitial lung disease (ILD)/pneumonitis that required steroids, has current ILD/pneumonitis, or suspected ILD/pneumonitis that cannot be ruled out by imaging at screening.
10. *Deleted criterion (amendment N°7)*
11. Known prior severe hypersensitivity to investigational product or any component in its formulation, including known severe hypersensitivity reactions to study drug (NCI CTCAE v5.0 Grade ≥3).
12. Patient has a history of severe hypersensitivity reactions to other monoclonal antibodies.
13. Patients previously treated with topoisomerase 1 inhibitor.
14. Patient has substance abuse or any other medical conditions that would increase the safety risk to the subject or interfere with participation of the subject or evaluation of the clinical study in the opinion of the Investigator.
15. Known history of testing positive for HIV or known acquired immunodeficiency syndrome.
16. Known active hepatitis B virus or hepatitis C virus infection at screening. Patients positive for hepatitis C (HCV) antibody are eligible only if polymerase chain reaction is negative for HCV RNA.
17. Active infection requiring systemic therapy (as IV antibiotics, antivirals, antifungals,…).
18. Other severe acute or chronic medical conditions or psychiatric conditions including recent (within the past year) or active suicidal ideation or behavior; or laboratory abnormalities that may increase the risk associated with study participation or study treatment administration or may interfere with the interpretation of study results and, in the judgment of the investigator, would make the patient inappropriate for entry into this study.
19. Patient with a history of symptomatic congestive heart failure (New York Heart Association Class II to IV), serious cardiac arrhythmia requiring treatment, history of myocardial infarction or troponin levels consistent with myocardial infarction 28 days prior to inclusion, or unstable angina within 6 months prior to inclusion, or current dyspnea at rest due to advanced malignancy. Patients with troponin levels above ULN at screening, and without any myocardial Infarction related symptoms should have a cardiologic consultation before enrollment to rule out myocardial infarction.

20. Patient with a corrected QT interval (QTc) prolongation to >470 ms (females) or >450 ms (males) based on average of the screening triplicate12-lead ECG.
21. Pregnant women, women who are likely to become pregnant or are breastfeeding or women who want donate, or retrieve for their own use, ova from the time of screening and throughout the study and for at least 7 months after last study treatment administration.
22. Patient with any psychological, familial, sociological or geographical condition potentially hampering compliance with the study protocol and follow-up schedule; those conditions should be discussed with the patient before registration in the trial.
23. Patient unwilling to participate to the biological investigations.
24. Individual deprived of liberty or placed under legal protection.
25. Patient with:
    - Lung-specific intercurrent clinically significant illnesses including, but not limited to, any underlying pulmonary disorder (i.e. e.g. pulmonary emboli within three months of the study randomization, severe asthma, severe COPD, restrictive lung disease, pleural effusion etc.),
    - or any autoimmune, connective tissue or inflammatory disorders (e.g., Rheumatoid arthritis, Sjogren's, sarcoidosis etc.) where there is documented, or a suspicion of pulmonary involvement at the time of screening.
    - or prior pneumonectomy.

PRIMARY ENDPOINT:

The primary endpoint is anti-tumor activity of DS-8201a carried out by the determination of the confirmed best objective response (BOR) rate in each cohort. The BOR is defined as the presence of a confirmed partial or complete response observed on treatment and assessed by investigators. The investigator will evaluate the objective response using RECIST v1.1 every 6 weeks.

For the cohort 3 (IHC0+), a short term primary endpoint is used for the interim analysis. The short term primary endpoint is the rate of patient without progression at 3 months. The investigator will evaluate the progression using RECIST v1.1 every 6 weeks.

SECONDARY ENDPOINT(S):

- The efficacy endpoint will be evaluated using RECIST v1.1 with the following parameters:
    - ➢ The BOR assessed by central review is defined as the presence of a confirmed partial or complete response observed on treatment and assessed by central review. This central review will evaluate the objective response using RECIST v1.1 every 6 weeks.
    - ➢ PFS is defined as the time from inclusion until progression or death from any cause, whichever occurs first. At the time of analysis, the patient alive and without progression will be censored at the date of the last tumor assessment

- ➢ DOR is applicable to subject with BOR, either complete response (CR) or partial response (PR), and is defined as the time from the first documented CR or PR until the date of disease progression, or until the date of death
- ➢ CBR is defined as the presence of at least a PR or CR, or a stable disease (SD) >6 months under treatment
- ➢ OS is defined as the time from inclusion until death. Patients alive at last follow-up will be censored at this date

- Safety will be evaluated continuously using NCI-CTCAE v5.0

TRANSLATIONAL ENDPOINT(S):

- Concentration of trastuzumab and deruxtecan in HER2 expressing and HER2 non expressing tumor cells at 24h after treatment initiation
- Percentage of stained cells and intensity of staining for immune response markers detected by IHC on tumor samples at baseline and 42 days after treatment initiation
- Percentage of stained cells and intensity of staining for biomarkers detected by IF and IHC on tumor samples at baseline
- Identification of genomic alterations of interest through whole exome sequencing and RNA sequencing on tumor and ctDNA samples collected after resistance to DS-8201a
- Describe the variation of CTC levels and evaluate its predictive value on the objective response, the clinical benefit, and survival
- Explore changes over time in tumor CTC phenotype induced by ADC

D) INVESTIGATIONAL MEDICINAL PRODUCTS

PRODUCT NAMES AND ADMINISTRATION:

Drug name (INN)	Registered name	Pharmaceutical form	Administration route	Posology
**DS-8201a** (Trastuzumab deruxtecan)	NA	Lyophilized powder	Intra-venous	5.4 mg/kg every 21 days

THERAPEUTIC REGIMENS:

DS-8201a will be administrated as an intra-venous injection of 5.4 mg/kg every three weeks (1 cycle = 21-day treatment).

TREATMENT DURATION:

DS8201a will be given until disease progression and/or unacceptable toxicity.

E) STATISTICAL ANALYSIS PLAN

REQUIRED NUMBER OF PATIENTS TO BE SCREENED/INCLUDED:

162 patients have to be included in three cohorts to answer the main question of this study:

- Cohort 1: 74 patients HER2 IHC3+ or IHC2+/ISH+
- Cohort 2: 44 patients HER2 IHC1+ or IHC2+/ISH-
- Cohort 3: 44 patients HER2 IHC0+

However, since the HER2 status is confirmed after inclusion, some switches may occur after inclusion from the initially allocated cohort to the confirmed HER2 status cohort.

Considering that cohort 2 will be the first to be terminated due to the higher frequency of HER2 IHC1+ or 2+/ISH- scores, we thus estimate that there will be around 25% of switches from cohort 1 and 3 to cohort 2, until they will be completed.

In order to reach the expected cohorts sample size, we estimate that around 30 additional patients may be necessary at the most to achieve the expected recruitment in cohort 1 and 3.

<u>COHORT 1 : HER2 IHC3+ or IHC2+/ISH+</u>

The primary endpoint of the trial is the rate of patients presenting an objective response. The following hypotheses are used:

- p0 = 30%, maximal unacceptable rate of patient presenting an objective response for whom the experimental treatment will be considered as insufficiently active.
- p1 = 45%, minimal acceptable rate of patients presenting an objective response for whom the experimental treatment will be considered as sufficiently active.

Using an A'Hern design (alpha = 5%, 1-beta = 80%) and (p0 = 30%; p1 = 45%), 67 patients need to be included in the study. The decision rules are summarized in the table below.

<u>Table: Number of patients required in the HER2 IHC3+ or IHC2+/ISH+ cohort</u>

Nb of patients	Insufficiently active	Sufficiently active
67 evaluable / 74 included	<27 successes	≥27 successes

Assuming a rate of around 10% of non-evaluable patients for the first assessment, to reach 67 evaluable patients, 67/90% = **74** patients HER2 IHC3+ or IHC2+/ISH+ have to be included in the cohort 1.

<u>COHORT 2 : HER2 IHC1+ or IHC2+/ISH-</u>

The primary endpoint of the trial is the rate of patients presenting an objective response. The following hypotheses are used:

- p0 = 20%, maximal unacceptable rate of patients presenting an objective response for whom the experimental treatment will be considered as insufficiently active.
- p1 = 40%, minimal acceptable rate of patients presenting an objective response for whom the experimental treatment will be considered as sufficiently active.

Using an A'Hern design (alpha = 5%, 1-beta = 85%) and (p0 = 20%; p1 = 40%), 40 patients need to be included in the study. The decision rules are summarized in the table below.

Table: Number of patients required in the HER2 IHC1+ or IHC2+/ISH- cohort

Nb of patients	Insufficiently active	Sufficiently active
40 evaluable / 44 included	<13 successes	≥13 successes

Assuming a rate of around 10% of non-evaluable patients for the first assessment, to reach 40 evaluable patients, 40/90% = **44** patients HER2 IHC1+ or IHC2+/ISH- have to be included in the cohort 2.

COHORT 3 : HER2 IHC0+

The primary endpoint is the rate of patients presenting a success. A success is defined as a patient presenting an objective response.

The short-term endpoint is the rate of patient without progression at 3 months. It will be analysed the FAS population. It will be assessed and described using frequency, percentage and 95% confidence intervals (Binomial exact).

The following hypotheses are used:

- Primary endpoint:

- p10=20%, maximal unacceptable rate of patients presenting a success for whom the experimental treatment will be considered as insufficiently active.
- p11=40%, minimal acceptable rate of patients presenting a success for whom the experimental treatment will be considered as sufficiently active.
-

- Short-term endpoint:

- p20=30%, maximal unacceptable rate of patients without progression at 3 months for whom the experimental treatment will be considered as insufficiently active.
- p21=50%, minimal acceptable rate of patients without progression at 3 months for whom the experimental treatment will be considered as sufficiently active.

Using a Kunz design with (alpha=5%, 1-beta=85%) and (p10=20%, p11=40%, p20=30% and p21=50%), 40 patients need to be included in the study and an interim analysis will be performed after inclusion of 16 patients. The decision rules are summarized in the table below:

Table: Number of patients required in the HER2 IHC0+ cohort

Step	Nb of patients	Insufficiently active	Sufficiently active
1	16 evaluable	≤ 4 non-progression	
2	40 evaluable/44 included	≤ 12 successes	≥ 13 successes

The recruitment will be interrupted after the inclusion of 16 evaluable patients to perform the pre-planned interim analysis. The recruitment in the cohort will be resumed or definitely interrupted according to the pre-defined stopping rules and IDMC decision.

Assuming a rate of around 10% of non-evaluable patients for the first assessment, to reach 40 evaluable patients, 40/0.9 = **44** patients HER2 IHC0+ have to be included in the cohort 3.

POPULATION TO BE ANALYZED:

- ✓ Full Analysis Set: All patients who received at least one dose of study drug and who had a valid first post-baseline assessment of disease status (or who had progressive disease)
- ✓ Safety population: The safety population will include all patients who received the study drug at least once

STATISTICAL ANALYSIS PLAN:

- ✓ Demographic data: Continuous variables will be summarized by cohort using median, minimum, maximum and number of available observations. Qualitative variables will be summarized by cohort using: counts, percentages, number of missing data.
- ✓ Primary endpoint will be analyzed on the FAS population. The primary endpoint of the phase II trial is the rate of patients presenting an objective response, defined as the best response observed while on treatment (local evaluation). It will be assessed and described by cohort using frequency, percentage and 95% confidence intervals (Binomial exact).

  Short term endpoint for cohort 3 (IHC0+) population will be analyzed on the FAS population. The short term endpoint of the cohort IHC0+ is the rate of patients presenting a non-progressive disease at 3 months according to RECIST v1.1 (local evaluation). It will be assessed and described using frequency, percentage and 95% confidence intervals (Binomial exact).

✓ Secondary endpoints

- Secondary efficacy endpoints will be analyzed on the FAS population:
  - For each cohort, survival rates (PFS, OS, and duration of response) will be estimated at different time points using the Kaplan-Meier method (with their respective confidence interval). Median survival times will be estimated by cohort with corresponding 95% confidence interval
  - Clinical benefit rates will be assessed and described by cohort using frequency, percentage, and 95% confidence intervals (Binomial exact)
- Safety analysis will be performed overall and per cohort on the safety population. Each of the following will be assessed by cycle and by patient:
  - AEs by toxicity grade (including fatal outcome)
  - SAEs
  - Treatment-related AE
  - AE leading to DS8201a dose reduction or interruption
  - Occurrence of AE with toxicity grade >2

  Frequency, percentage with 95% confidence interval will be computed for each event.

F) SAMPLES COLLECTED FOR TRANSLATIONAL RESEARCH

1/ Mandatory tumor samples to be obtained for all the patients

- Tumor biopsy at baseline (1 frozen + 2 formalin-fixed paraffin-embedded [FFPE] blocks)
- Tumor biopsy at disease progression (primary resistance PFS <6 months; secondary resistance PFS ≥6 months) (1 frozen + 2 FFPE)

2/ Mandatory tumor samples to be obtained for HER2 3+ or HER2 2+/ISH+ patients

Tumor biopsy at 6 weeks (D43) after treatment initiation (30 patients) (1 frozen + 1 FFPE block)

3/ Optional tumor samples to be obtained for HER2 2+/ISH- or HER2 1+ or HER2 0+ patients

Tumor biopsy at 6 weeks (at D43) after treatment initiation (1 frozen + 1 FFPE block)

4 / Mandatory blood samples for all the patients

- Whole blood sample at baseline
- Blood for ctDNA and CTC at baseline, post-C1-C2-C4-C6, and at the end of treatment (whatever the reason)

5/ Research conducted in selected centers

Optional tumor biopsy at 24h (D2) after treatment initiation (10 patients) (1 frozen + 1 FFPE blocks)<br>Note that patients who accept to undergo a biopsy at 24h post-C1 may not be the same than those who accept a biopsy at D43 post-C1.

**G) TRIAL DURATIONS**
INCLUSION PERIOD: 18 months
MEDIAN TRIAL TREATMENT PERIOD: 12 months
POST-TREATMENT FOLLOW-UP: 36 months
DURATION UNTIL PRIMARY ENDPOINT EVALUATION: 24 months
OVERALL TRIAL DURATION (INCLUDING FOLLOW-UP): 66 months

TRIAL SCHEME

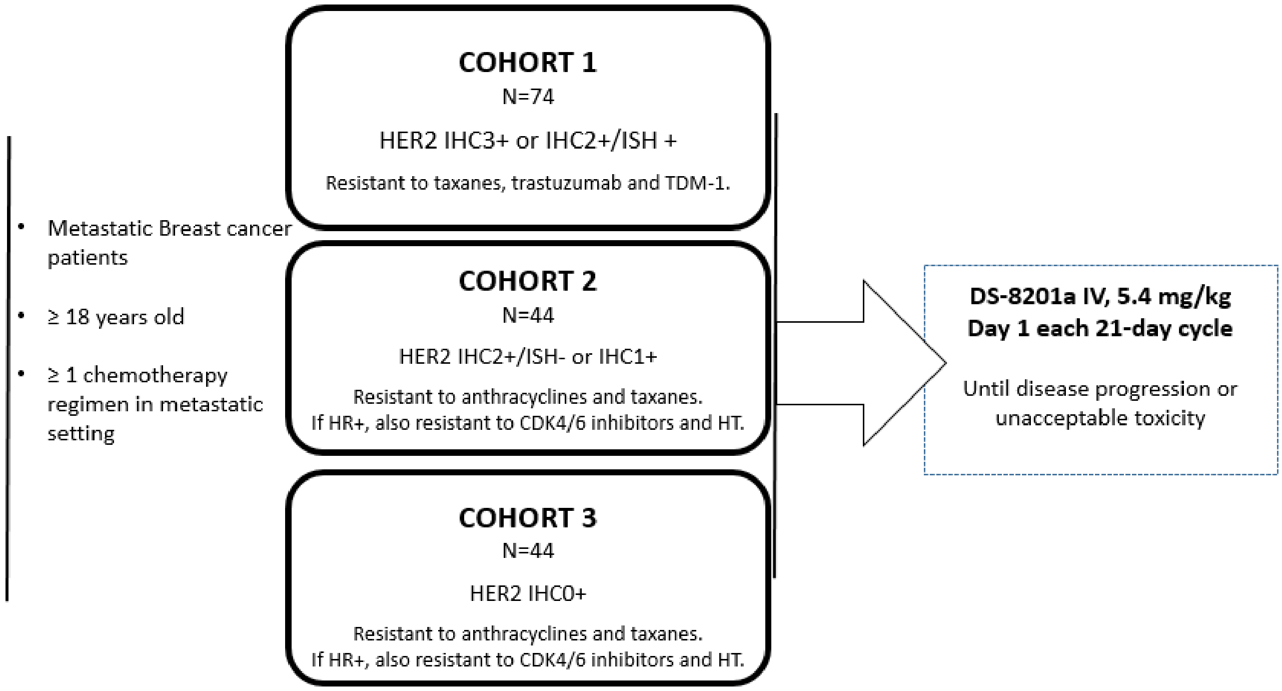

SCHEDULE OF VISITS AND ACTIVITIES (SOA)

A general +/-3 days window is allowed for assessments or visits performed every 3 weeks. 1 Cycle=21 days
A +/-7 days window is allowed for tumor evaluations.

	Baseline		Treatment phase						Follow-up
**CYCLES Every 3 weeks**	**≤28 days**	**≤14 days**	**C1**	**C2**	**C3**	**C4**	**Cn**	**End of Treatment**	**Every 12 weeks and up to 3 years**
**Visits**			D1	D1	D1	D1	D1	6 weeks after last administration	
Signed informed consent form	X								
E-CRF registration (1)	X								
**PATIENT HISTORY**									
Inclusion / non-inclusion criteria	X								
Demographic data	X								
Cancer history and characteristics / prior therapies	X								
Other relevant medical history	X								
Collection of concomitant treatments (2)		X	D1	D1	D1	D1	D1	X	
Collection of first post-study new antineoplastic treatments									X
Disease and survival status									X
**SAFETY ASSESSMENTS**									
CLINICAL EXAMINATION									
Physical examination		X	D1	D1	D1	D1	D1	X	
SpO_2		X	D1 (21)	D1 (21)	D1 (21)	D1 (21)	D1 (21)	X	
Height		X							
Weight, PS (ECOG)		X	D1	D1	D1	D1	D1	X	
Vital signs (blood pressure, pulse, body temperature, respiratory rate, oxygen saturation of the blood)		X	D1	D1	D1	D1	D1	X	
Adverse Event (3)	X		ongoing	ongoing	ongoing	ongoing	ongoing	X	X
PARACLINICAL EXAMINATION									
ECG 12 leads (4)	X					X		X	
LVEF (5)	X						X (5)	X	
Ophthalmologic examination (6)	X							X	

	Baseline		Treatment phase						Follow-up
**CYCLES Every 3 weeks**	**≤28 days**	**≤14 days**	**C1**	**C2**	**C3**	**C4**	**Cn**	**End of Treatment**	**Every 12 weeks and up to 3 years**
**Visits**			D1	D1	D1	D1	D1	6 weeks after last administration	
BIOLOGICAL TESTS (7)									
Haematological		X	D1, D8, D15	D1	D1	D1	D1	X	
Coagulation parameters		X							
Biochemistry		X	D1, D8, D15	D1	D1	D1	D1	X	
Troponin	X							X	
Pregnancy test (8)		X						X	X(22)
**TREATMENT ADMINISTRATION**									
DS-8201a (9)			D1	D1	D1	D1	D1		
**ACTIVITY ASSESSMENT**									
TUMOR EVALUATION									
Clinical disease assessment	X				D1		D1	X	X
Radiologic assessment (by RECIST v1.1) – Bone Scan	X (20)		*As clinically indicated* (10)						*As clinically indicated* (10)
Radiologic assessment (by RECIST v1.1) – Brain MRI or tomography	X		*As clinically indicated* (10)						*As clinically indicated* (10)
Radiologic assessment (by RECIST v1.1) – TAP-CT scan or MRI	X				D1 (12)		D1 (12)	X (13)(14)	X (13)
**ANCILLARY STUDIES FOR ALL THE CENTERS**									
BIOLOGICAL SAMPLES : MANDATORY FOR ALL PATIENTS									
Biopsy of a metastatic site	X (15)							X (15)	
Blood sample (30 mL for ctDNA and CTC)	X			D1 (17)	D1 (17)		D1-C5 and D1-C7 ((17)	X (18)	
Whole blood sample (5 mL) for constitutional DNA analysis	X								
ADDITIONNAL TUMOR SAMPLES : MANDATORY FOR HER2 OVEREXPRESSED PATIENTS (cohort 1)									
Biopsy of a metastatic site					D1 (16)				
ADDITIONNAL TUMOR SAMPLES : OPTIONNAL FOR HER2 LOW and NULL PATIENTS (cohort 2&3)									
Biopsy of a metastatic site					D1 (16)				
**BYSTANDER EFFECT STUDY : SELECTED CENTERS**									

CYCLES Every 3 weeks	Baseline ≤28 days	Baseline ≤14 days	Treatment phase C1	C2	C3	C4	Cn	End of Treatment	Follow-up Every 12 weeks and up to 3 years
Visits			D1	D1	D1	D1	D1	6 weeks after last administration	
Biopsy of a metastatic site (optional for the patients)			D2 (19)						

(1) Patients will begin study treatments within 5 days of registration.

(2) Data concerning all treatments received within 2 weeks of enrolment. Check the current administration of prohibited medications and start considering substituting them.

(3) Ongoing toxicities or adverse event must be monitored until resolution or returned to baseline level.
Information must be collected concerning adverse events occurring during the 47 days after the last administration of DS-8201a. Any late Serious Adverse Drug Reaction (SAE related to an investigational product), occurring at any time after this period must be reported to the pharmacovigilance department of R&D UNICANCER.

(4) ECG will be taken in triplicate at screening. Subsequent ECGs will be performed every 4 cycles, as single measure and in triplicate if an abnormality is noted. ECGs will be taken in close succession, while in a supine/semi-recumbent position. Following parameters will be systematically measured ; RR, PR, QT intervals, and QRS duration measures.

(5) LVEF will be performed at baseline, on D1 of cycle 5 and then every 4 cycles. The same method should be used throughout the study (Echo or MUGA).

(6) Ophthalmologic assessments including visual acuity testing, slit lamp examination and fundoscopy. Additional ophthalmologic assessments will be performed if clinically indicated.

(7) Any result outside the normal range or inclusion range will be repeated, at the discretion of the investigator.
Test to be performed are described in the table below:

**Test Category**	**Test Name**
Hematology	Red blood cell count, hemoglobin, hematocrit, platelet count, white blood cell count, differential white blood cell count (neutrophils, lymphocytes, monocytes, eosinophils, basophils)
Coagulation profile	Activated partial thromboplastin time (APTT) or partial thromboplastin time (PTT), international normalised ratio (INR)
Biochemistry	Albumin, alkaline phosphatase[a], ALT (SGPT)[a], AST (SGOT)[a], calcium, chloride, serum creatinine, lactate dehydrogenase (LDH), magnesium, potassium, sodium, total bilirubin[a], total protein, urea or blood urea nitrogen, depending on local practice
Troponin	Preferably high-sensitivity troponin-T, at screening (-28 days), and if at any time a subject reports signs or symptoms suggesting congestive heart failure, myocardial infarction, or other causes of myocyte necrosis Patients with troponin levels above ULN at screening, and without any myocardial Infarction related symptoms should have a cardiologic consultation before enrollment to rule out myocardial infarction.

All laboratory analysis will be performed locally.

[a] Tests for ALT, AST, alkaline phosphatase, and total bilirubin must be conducted and assessed concurrently. If total bilirubin is ≥2 × upper limit of normal (and no evidence of Gilbert's syndrome) then fractionate into direct and indirect bilirubin.

(8) Pregnancy test (pre-menopausal patients only): Urine test within 72 h prior to enrolment, or serum pregnancy test within 14 days prior to enrolment.

(9) Study treatment administration: DS-8201a will be administered IV, 5.4 mg/kg Q3W, at D1 of a 3-week cycle, until disease progression or unacceptable toxicity.

(10) To be performed in case of known bone metastases or if clinically indicated. Can be replaced by PET-CT if the histological type is not lobular and if a PET-CT has been achieved at baseline.

(11) To be performed in case of known CNS metastases or if clinically indicated.

(12) CT scan or MRI of the chest, abdomen and pelvis should be performed for RECIST v1.1 assessment. Imaging disease assessments should be obtained every 6 weeks (2 cycles +/-7 days), or sooner if clinically indicated, during the first 12 months of treatment phase, and every 12 weeks (+/- 7 days) thereafter. They should be repeated at least 4 weeks after assessment of a PR or CR as per RECIST v1.1 guidelines.

(13) Tumor evaluation has to be continued during the post-treatment period if withdrawal was not related to disease progression and should be continued and documented every 6 weeks (or every 12 weeks after the first 12 months of treatment phase) until disease progression or initiation of an antineoplastic treatment.

(14) Radiological assessments should not be repeated if they were obtained less than 6 weeks from withdrawal of therapy.

(15) When possible up to 3 cores (1 frozen, 2 FFPE) will be collected from a metastatic site (or primary when stage 4 at diagnosis) at baseline and at disease progression. When patients already have an archived biopsy from a secondary or a primary site (if stage 4) of their current disease, this material can be used for the study, provided that: it was collected within 3 months prior enrolment, and a frozen and a FFPE sample are both available for research (see section in protocol). Some specific conditions apply to the centers who participate to the "bystander effect study", refer to note 19.

(16) When possible, up to 2 cores (1 frozen, 1 FFPE) will be collected 6 weeks (+/- 7days) after treatment initiation. Biopsy must be done on the same site as baseline sample. This biopsy is mandatory for patients with a HER2 3+ or HER2 2+/ISH+ status and is optional for other patients (HER2 2+/ISH- or HER2 1+ or HER2 0+).

(17) At D1 before DS-8201a administration.

(18) Whatever the reason of the treatment termination.

(19) When possible, up to 2 cores (1 frozen, 1 FFPE) will be collected at 24h after treatment initiation (D2C1) on the same organ as baseline. When H24 biopsy is very close to the previous one (less than 3 weeks), it must be done on the same organ but not on the same lesion.
To facilitate the acceptance of the H24 biopsy, patients can skip the baseline biopsy if they have an archived biopsy from a secondary or a primary site (if stage 4) of their current disease, provided that : a) sample was collected less than 1 year from the inclusion date; b) at least 1 FFPE is available; c) the patient can undergo a H24 biopsy in the same organ as the archived samples.

(20) Can be replaced by PET-CT if the histological type is not lobular.

(21) Before Infusion AND at the End of Infusion.

(22) During the 2nd follow-up visit at 6 months.

1. INTRODUCTION

1.1. Background information

1.1.1. *Disease/pathology epidemiology/prognosis*

Breast cancer remains the most common cancer and the second leading cause of cancer mortality in women in the world. Despite advances in our knowledge of the underlying genetic alterations in breast cancer and improvements in treatment, 20% to 30% of patients with early breast cancers still relapse with distant metastatic disease (1).

These patients either present with metastases at diagnosis (3 to 5% of breast cancer cases) or are resistant to the conventional treatments administered prior to or after the surgery. Even though recent progress has led to a decrease in the incidence of metastases, this decrease will probably hit a plateau of 10% for the breast cancer cases corresponding to stage IV patients and a plateau of 5-7% for the residual risks of metastases after adjuvant medical therapy. This 10% of breast cancer cases represents a population of around 5000 new cases a year in France.

Once it reaches the metastatic stage, breast cancer is considered incurable in most cases. Many publications suggest that the 5-year survival rate is improving (2). Nevertheless, most patients will die from their cancer.

This introduction thus underlines the need for new strategies for treating metastatic breast cancer.

1.1.2. *Disease current treatment*

From a therapeutically point of view, three distinct groups of patients can be differentiated based on their tumor biological characteristics: 1) the estrogen receptor (ER) and/or progesterone receptor (PR) positive subtype (essentially comprising Luminal A and B tumors) that forms the most common group (60-70%) with patients eligible to endocrine therapy; 2) the HER2 amplified subtype eligible to effective HER2-targeted therapies (3); and 3) basal-like carcinoma that represents 10-15% of all invasive breast cancer with therapeutic choices limited to chemotherapy (4). The latter subtype is referred to as triple-negative (TNBC) because of the lack of expression of estrogen and progesterone receptors and the absence of amplification of ERBB2 oncogene (5).

General disease management in HER2 negative advanced disease patients.

Standard of care in metastatic setting for HER2 negative patients mainly relies on anthracyclines and/or taxanes-based chemotherapy as first line treatment. When patients have been previously exposed to this drugs in the (neo)-adjuvant, or present some medical contraindications, capecitabine, vinorelbine and eribuline are the preferred choices (6). However, capecitabine often is considered as the most appropriate treatment for patients with HER2 negative advanced disease because of the oral administration and the favorable safety profile that provide quality of life advantages over the intravenous chemotherapy alternatives (7). Moreover, many phase 3 pivotal studies consider the capecitabine-pretreated patients as the population of reference for the evaluation of a new agent.

However, management of progressive metastatic disease beyond the first line is still challenging since no clear and optimal strategy has been defined yet.

For HER2 negative hormone receptor positive (HR+) patients, estrogen and progesterone hormone receptor (HR) expression status is one of the most important prognostic factors in invasive breast cancer. Estrogen deprivation therapy is the core treatment modality in patients with HR+ advanced breast cancer. Recently, palbociclib, a Cyclin-Dependent Kinases 4 and 6 inhibitor (CDK4/6i), has been approved in Europe in combination with aromatase inhibitors or with fulvestrant, as first or later line hormone therapy for pre and postmenopausal women. However, unless chemotherapy appears as the best option because of poor general status, visceral crisis or short PFS under previous hormone therapy, the optimal sequence of endocrine-based therapy is still uncertain and depends on which agents were previously used, the disease presentation, and the patient's preference (8, 9).

General disease management in HER2 positive patients

HER2 gene amplification or HER2 receptor overexpression are present in around 20% of all breast cancers and has been associated with biologically aggressive disease and shortened overall survival (OS) (10). Trastuzumab plus chemotherapy, as well as the combined use of trastuzumab plus pertuzumab and chemotherapy, have improved median progression-free survival (PFS) from 12.4 to 18.7 months (hazard ratio [HR]=0·69; 95% confident interval [CI], 0·58 to 0·81) and median OS from 40.8 months (95% CI, 35.8 to 48.3) to 56.5 months (95% CI, 49.3 to not reached) ((11-14). These results lead to a new standard of care for patients with metastatic breast cancer who have not received prior anti-HER2 therapy or chemotherapy for metastatic disease.

Trastuzumab emtansine (T-DM1) is an antibody drug conjugated composed of the humanized monoclonal antibody trastuzumab stably linked to the cytotoxic microtubule inhibitor DM1 (15). Regulatory agencies from many countries worldwide approved T-DM1 for the treatment of HER2-positive metastatic breast cancer in patients previously treated with trastuzumab and a taxane (separately or in combination), and who have received previous therapy for metastatic breast cancer. T-DM1 demonstrated to improve median PFS from 6.4 to 9.6 months (HR=0.65; 95% CI, 0.55 to 0.77; P<0.001) and median OS from 25.1 to 30.9 months (HR=0.68; 95% CI, 0.55 to 0.85; P<0.001) and is now considered the standard of care for this indication (6, 16-18).

1.1.3. *Overview of DS-8201a, the investigational medicinal products (IMP)*

(*Refer to the current version of the DS-8201a investigator brochure (IB) for a complete summary of non-clinical and clinical data including safety, efficacy and pharmacokinetics data.)*

Pre-clinical activity

DS-8201a is an antibody-drug conjugate (ADC) comprised of a recombinant humanized anti-HER2 immunoglobulin G1 monoclonal antibody produced in-house with reference to the same amino acid sequence of trastuzumab, covalently conjugated to a drug-linker. The released drug (a derivative of exatecan) inhibits topoisomerase I and leads to apoptosis of the target cells.

In studies on the mechanism of action of DS-8201a, DS-8201a was confirmed to have an HER2-mediated Akt phosphorylation inhibition effect and an antibody dependent cellular cytotoxic (ADCC) activity, and has also been confirmed to cause deoxyribonucleic acid (DNA) damage and induce

apoptosis, effects that are assumed to be the result of the exatecan derivate, which has topoisomerase I inhibitory activity.

Therefore, DS-8201a is considered to exhibit HER2-specific cell growth inhibition and antitumor activity via a novel mechanism of action that combines the pharmacological activities of the HER2 antibody component, with those of the drug component.

In nonclinical models, DS-8201a showed a much broader antitumor spectrum than T-DM1, including efficacy against T-DM1 resistant and HER2 low-expressing tumors. In vivo studies using a tumor-bearing mouse model suggest that administration of DS-8201a results in the regression of HER2-positive tumors. DS-8201a has a cytotoxic drug component with a different mechanism of action than that of T-DM1 and is expected to show activity in tubulin inhibitor insensitive tumors, so it is anticipated to be of benefit in the T-DM1-refractory patient population (19).

Moreover, the linker-drug combination of DS-8201a allows for a higher drug-to-antibody ratio (DAR ≈ 8) compared to T-DM1 (DAR ≈ 3.5). The higher DAR of DS-8201a may help target low expressing HER2 tumors by supplying more payload per antibody to a tumor.

Additionally, in vitro and in vivo experiments using a mixture of HER2 positive and negative cells revealed the killing effect of DS8201a on both type of cells, only when negative cells were neighboring positive cells. In the same conditions, T-DM1 failed to show a comparable activity on negative cells (20). These results indicated that DS-8201a has a potent bystander effect due to a highly membrane-permeable payload and affects cells in close proximity, regardless of their level of HER2 expression. It may be therefore beneficial in treating tumors with HER2 heterogeneity that are unresponsive to T-DM1.

Clinical activity in HER2 over-expressing patients

In the Phase 1 clinical study DS8201-A-J101, results from HER2-positive breast cancer subjects pretreated with T-DM1 showed that almost all subjects experienced tumor shrinkage with durable disease control. Subjects evaluable for confirmed responses (at least 2 post-baseline scans, total n=111 showed overall response rate (ORR) of 59.5% (66/111) (95% CI 49.7, 68.7) and a median duration of response of 20.7 months (range 0.0+, 21.8+).

The current Kaplan-Meier estimate for median PFS reached 22.1 months (95% CI not estimable, range 0.8-27.9) and the median OS was not reached. In the population of HER2-positive breast cancer subjects who received pertuzumab pretreatment (a subset of the T-DM1 treated population), an ORR of 62.5% (total of 60 responders out of 96 subjects) and a disease control rate (DCR) of 93.8% was observed (21). These results compare favorably with the historical results reported for HER2-positive breast cancer treatments. This, despite the use of DS-8201a in a later line of therapy.

DS-8201a exhibited a potent antitumor activity in a broad selection of HER2-positive models and favorable pharmacokinetics and safety profiles. The results demonstrate that DS-8201a will be a valuable therapy with a great potential to respond to T-DM1-insensitive HER2-positive cancers and low HER2-expressing cancers. This suggest that DS-8201a may be efficacious in a broader population of HER2-positive cancer patients and confirm the importance of this new class of novel topoisomerase I inhibitor-based ADC (19).

Clinical activity in HER2 negative patients (HER2 low or non expressing)

In the Phase 1 clinical study DS8201-A-J101, the activity of DS-8201a was investigated on HER2 low expressing tumor (IHC2+/ISH negative and IHC1+). Subjects evaluable for confirmed responses showed an overall response rate (ORR) of 44.2% (19/43) and a duration of response of 9.4 months (range 1.5+, 23.6+). Median PFS was 7.6 months (95% CI 4.9, 13.7). Interestingly, Ds-8201s exhibited an activity even in the IHC1+ subpopulation and in multi-resistant populations after exposure to CDK4/6 inhibitors, with an ORR of 33.3% (22).

Clinical activity in HER2 nul-expressing patients

In the Phase 1 clinical study DS8201-A-J101, the HER2 status was locally assessed and only patients with HER2-overexpressing tumors were eligible. However, a retrospective central re-analysis of these tissue samples showed that 11 of the breast cancer patients enrolled in the DS8201-A-J101 study (1 in part 1, 10 in part 2) had, in fact, HER2-negative (IHC 0) tumors. Interestingly, and against all odds, DS8201 had antitumor activity in these 11 patients presenting with HER2-negative breast cancer (unpublished Daiichi's data). The best tumor response was a partial response for 5 HER2-negative breast cancer patients and stable disease for 6 HER2-negative breast cancer patients.

1.2. Trial rationale

This trial protocol intends to evaluate the efficacy of DS-8201a in all comer patients after at least one line of chemotherapy for treatment of their metastatic disease, regardless of their HER2 status, after all standard options have been exhausted.

Despite these important therapeutic advances, most patients inevitably progress on therapy due to primary or acquired resistance, or because of an incorrect HER2 positivity assessment. Hence, it is crucial to correctly categorize HER2 tumors and define mechanisms of resistance of new treatments. In addition, identification of biomarkers for drug response or resistance allows tailoring the therapeutic options for each patient sparing them from unnecessary toxicity as well as improving their outcomes. The aim of this trial is to assess the activity of DS8201a in HER2-positive and HER2 low and non-expressing metastatic breast cancer referring to the mechanisms of action.

Rationale in HER2 over-expressing patients

Although anti-HER2 targeted therapies have improved patients outcomes, they are not curative in the metastatic setting. Treatment options for patients who have progressed after 2 lines of anti-HER2 therapy remain unclear and limited. Current options include lapatinib plus capecitabine, trastuzumab plus capecitabine, trastuzumab plus lapatinib, or trastuzumab plus other agents. Reported response rates for these regimens when given as second lines of anti-HER2 therapy range from 10% to 22% (6). Therefore, treatment options for HER2-positive breast cancer remain limited, with no targeted therapy specifically approved following trastuzumab, pertuzumab, and T-DM1 failure proving of high unmet medical need.

In consequence, new treatment options need to be developed to improve outcomes for patients with disease progression following failure of trastuzumab, pertuzumab, and T-DM1 regimens.

DS-8201a is a HER2-targeting ADC with a high drug-to-antibody ratio (7 to 8), and a novel topoisomerase I inhibitor as payload (see Section 1.1.3). Nonclinical and clinical evidence demonstrates that the HER2 targeting of DS-8201a is highly specific. DS-8201a mechanisms' of action predict a sensitivity of tumors previously refractory to TDM-1 treatment.

Rationale in HER2 negative patients

To date, the standard of care for HER2 negative and ER positive tumors is the combination of endocrine therapy and a CDK4/6 inhibitor (6). However, the disease will eventually progress and patients receive cytotoxic chemotherapy. The main cytotoxic drugs for breast cancers are anthracyclines, taxanes and capecitabine. After progression, others option are eribuline, gemcitabine or vinorelbine. However, many of these drugs are targeting microtubules and resistance occurs. Considering the mechanism of action of DS-8201, an antibody drug conjugate with a topoisomerase 1 inhibitor, it is relevant to address its activity in HER2 negative tumors. The objective response rate of 50% observed in phase I/II trial compared very favorably with the activity of cytotoxic chemotherapy in that subtype of breast cancer (23).

HER2 negative population comprises the subset of IHC0 patients for which very scare but encouraging data are available to date. However, considering the DS-8201a mechanism of action, the very low level of HER2 expression ranging from 10% to 0% in this category may be sufficient to observe a clinical activity through its bystander effect on proximal HER2 negative cells.

The potential activity of DS8201 in HER2 low/nul-expressing tumors is a major improvement compared to all HER2-targeted monoclonal antibodies already approved. Thus, the activity of DS-8201 in HER2 low expressing tumors is of interest and needed to be addressed.

As a general conclusion, despite these important therapeutic advances, most patients inevitably progress on therapy due to primary or acquired resistance, or because of an incorrect HER2 positivity assessment. Hence, it is crucial to correctly categorize HER2 tumors and define mechanisms of resistance of new treatments. In addition, identification of biomarkers for drug response or resistance allows tailoring the therapeutic options for each patient sparing them from unnecessary toxicity as well as improving their outcomes. The aim of this trial is to assess the activity of DS8201a in HER2-positive and HER2 low and non-expressing metastatic breast cancer referring to the mechanisms of action.

1.3. Justification for the therapeutic regimens and treatment durations

A dose of 5.4 mg/kg is the recommended dose for HER2-positive breast cancer in the DS8201-A-U201 continuation phase II study (NCT03248492) and the planned dose for further clinical studies in all breast cancer populations.

1.4. Potential risks and benefits

1.4.1. *Known potential risks*

ILD and neutropenia including febrile neutropenia are considered to be important identified risks based on available nonclinical, clinical, epidemiologic information, and literature review of products of similar class as trastuzumab deruxtecan. For the clinical data, evaluation of important identified risks is primarily based on the safety data from the primary analysis of Studies DS8201-A-J101, and DS8201-A-U201 because these studies have the longest durations of exposure and largest sample sizes. Please refer to the DS-8201 IB section 6 for more information.Left ventricular dysfunction is classified as an important potential risk. Embryo-fetal toxicity is also
classified as an important potential risk.

1.4.2. *Known potential benefits*

In the DESTINY-Breast01 trial (24), 184 patients were included with a median of 6 previous lines of treatment to receive trastuzumab deruxtecan at the dose of 5.4 mg/kg. The treatment induced a confirmed objective response rate (ORR) of 60.9% (95% CI, 53.4-68). Among these patients, 6.0% had a complete response and 54.9% had a partial response, with no consistent differences in response rates when considering specific subgroups.

Trastuzumab deruxtecan showed durable antitumor activity in the HER-2 positive metastatic breast cancer patients: the median response duration was 14.8 months (95% CI, 13.8 to 16.9) and the median duration of progression-free survival was 16.4 months (95% CI, 12.7 to not reached) among all patients. Estimated overall survival was 93.9% (95% CI, 89.3 to 96.6) at 6 months and 86.2% (95% CI, 79.8 to 90.7) at 12 months; the median overall survival was not reached at the time of the time of the analysis.,.

Based on the phase II DESTINY-Breast01 trial, FDA granted an accelerated approval for the treatment of adult patients with unresectable or metastatic HER2-positive breast cancer who have received at least 2 prior lines of anti–HER2-based regimens in the metastatic setting.

In the same study, DS-8201a showed antitumor activity in HER2-low expressing breast cancer patients: the objective response rate of 46% is comparable with the activity of cytotoxic chemotherapy in that subtype of breast cancer.

Finally, potential benefits may be expected in HER2 non-expressing patients, but proof of concept should be confirmed in patients with no valid therapeutic medical options.

1.5. Trial population

Each patient will sign an informed consent form provided by the site. A patient will be considered enrolled in the study upon the investigator will obtained written informed consent. The trial population is composed of individuals, aged at least 18 years, with metastatic breast cancer. Patients should have received at least 1 line of therapy in the metastatic setting, and must not be amenable to any other validated therapeutic option. All patients should have been treated by prior anthracyclines and taxanes, either in (neo)-adjuvant or in metastatic setting, and by capecitabine for HER2 negative patients. Additionally, HR+ tumor must be resistant to endocrine therapy and CD4/6 inhibitors.

Patients with HER2-over expressing (IHC3+ or IHC 2+/ISH+) tumors must be resistant to trastuzumab and TDM-1.

2. TRIAL OBJECTIVES

2.1. Primary objective

The main objective is to evaluate the anti-tumor activity of DS-8201a in three cohorts of advanced breast cancer patients:

- a cohort of HER2 over-expressing (HER2 IHC3+ or HER2 IHC2+/ISH+)
- a cohort HER2 low-expressing (IHC1+ or IHC2+/ISH-)
- a cohort HER2 non-expressing (IHC0+)

2.2. Secondary objective(s)

- To evaluate the efficacy in each cohort, in term of:
  - Best objective response as assessed by central review
  - Progression Free Survival (PFS)
  - Duration of response (DOR)
  - Clinical benefit rate (CBR)
  - Overall survival (OS)
- To evaluate the safety of DS-8201a overall and per cohort by NCI-CTCAE v5.0

3. TRIAL DESIGN AND ENDPOINTS

3.1. Description of the trial Design

This is a multicenter, international open-label phase II trial assessing the efficacy of DS-8201a monotherapy on patients with metastatic breast cancer (Figure 1).

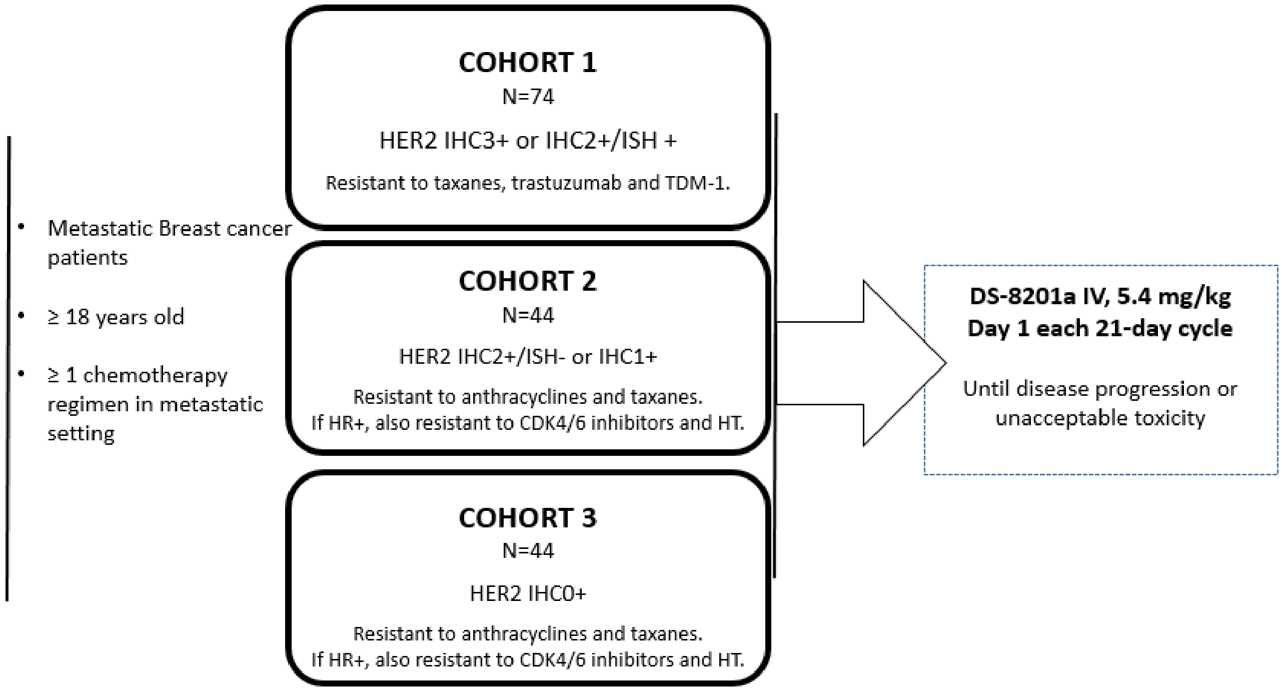

Figure 1. *Study Scheme*

3.2. Trial Endpoints

3.2.1. *Primary endpoint*

The primary endpoint is anti-tumor activity of DS-8201a, carried out by the determination of the confirmed best objective response (BOR) rate in each cohort. The BOR is defined as the presence of a confirmed partial or complete response observed on treatment and assessed by investigators. The investigator will evaluate the objective response using RECIST v1.1 every 6 weeks.

For the cohort 3 (IHC0+), a short term primary endpoint is used for the interim analysis. The short term primary endpoint is the rate of patient without progression at 3 months. The investigator will evaluate the progression using RECIST v1.1 every 6 weeks.

3.2.2. *Secondary endpoint(s)*

- The efficacy endpoint will be evaluated using RECIST v1.1 with the following parameters:
  - ➢ The BOR assessed by central review is defined as the presence of a confirmed partial or complete response observed on treatment and assessed by central review. This central review will evaluate the objective response using RECIST v1.1 every 6 weeks
  - ➢ PFS is defined as the time from inclusion until progression or death from any cause, whichever occurs first. At the time of analysis, a patient alive and without progression will

be censored at the date of the last tumor assessment. Progression will be defined by investigator using RECIST v1.1

- DOR is applicable to subject with BOR, either CR or PR, and is defined as the time from the first documented CR or PR until the date of disease progression, or until the date of death
- CBR is defined as the presence of at least a PR or CR, or a SD >6 months
- OS is defined as the time from inclusion until death. Patients alive at last follow-up will be censored at this date

- Safety will be evaluated continuously using NCI-CTCAE v5.0
- Tumor tissue and blood samples collection will be used to characterize biomarkers for response and/or resistance to DS-8201a (refer to Appendix 6 for description of translational objectives)

3.3. Progression of the trial

Patients participating in the trial will comply with the protocol for a total number of 4 years after inclusion in the study.

The end of the study corresponds to the last visit of the last patient.

The investigation/examination schedule is defined by the trial flow-chart provided in section "Schedule of visits and activities" of the protocol summary.

3.4. Inclusion procedure

After the eligibility criteria have been fulfilled and the patient consent has been obtained, the patient will be registered in the study using the registration form on R&D UNICANCER's online electronic case report form (eCRF) website (Ennov Clinical® software):

https://ecrf.icm.unicancer.fr/CSOnline/

Once the patient has been registered, automatic e-mails confirming registration will be sent to the:

- sponsor
- investigator
- data manager
- statistician

The eCRF registration procedure will be explained to the center staff during the study initiation visit. The corresponding documents, with explanation of the procedure, will be given to the principal investigator of each center and filed in the Site Master File (SMF).

3.5. Premature Trial Terminations and Suspension

The trial can be suspended or stopped by the sponsor after meeting with the coordinating investigator or following a request by the respective regulatory authority and/or the responsible Ethics Committee for the following reasons:

- High frequency and/or unexpected severity of toxicity
- Insufficient patient enrolment
- Insufficient quality of data collection

3.6. Patient's trial withdrawal and discontinuation

Patient withdrawal concerns patients who stop treatment and all other protocol-defined procedures. Please note that treatment discontinuation without consent withdrawal from a patient is not considered as a trial withdrawal. For treatment discontinuation only, please refer to section 5.8. This can occur under the following circumstances:

- Patient withdraws consent
- The principle investigator may terminate a patient's participants from the trial, if this is in the interest of the patient

Trial patients may withdraw their consent at any time without justification, irrespective of the reason(s). In the case of trial withdrawal the investigator should attempt to obtain as much information as possible. This information should be noted in the patient's medical file. The patient's withdrawal of consent does not impact the patient's right to receive medical treatment.

4. PATIENT SELECTION

4.1. Diagnosis and inclusion criteria

Patients must meet all of the following criteria to be included in the study:

1. Patient must have signed a written informed consent form prior to any study specific procedures. When the patient is physically unable to give its written consent, a trusted person of their choice, independent from the investigator or the sponsor, can confirm in writing the patient's consent.
2. Female or male subjects aged ≥18 years.
3. Patient with histologically-confirmed diagnosis of invasive breast cancer. Tumors can be either HER2 IHC3+ or IHC2+/ISH positive or IHC2+/ISH negative or IHC1+ or IHC0+, on most recent tumor tissue sample available.
4. Patient with a documented radiologic metastatic progression.
5. Patient considered by the investigator as not amenable to any other validated therapeutic option, after at least 1 line of chemotherapy in metastatic setting:

- All patients with HER2 over-expressing (IHC3+ and IHC2+/ISH+) (and hormone receptor positive or negative) tumor must have been treated with taxanes and have progressed on trastuzumab and on TDM-1. Prior treatment with pertuzumab is not required.
- patient with HER2 negative (IHC0, 1+, and 2+/ISH-) tumor must have been treated with anthracyclines and taxanes. Patients with hormone receptor positive (ER+ and/or PR+) tumor must be resistant to endocrine therapy and CDK4/6 inhibitors.

6. Non-bone metastatic site easily accessible to biopsy.
7. Presence of at least one radiologically measurable lesion according to RECIST v1.1.
8. Patient with WHO performance status ≤1.
9. Adequate bone marrow function: absolute neutrophil count (ANC) ≥1.5 × 10^9/L, platelet count ≥100 × 10^9/L, and hemoglobin ≥9 g/dL (transfusion is not allowed within 1 week prior to baseline assessment).
10. Adequate liver function: total bilirubin level ≤1.5 × the upper limit of normal (ULN) range if no liver metastases or <3 x ULN in the presence of documented Gilbert's Syndrome or liver metastases. AST and ALT levels ≤3 × ULN (AST and ALT≤ 5 ULN when documented liver metastasis).
11. Adequate blood clotting function: International Normalized Ratio (INR)/Prothrombin Time (PT) and either partial thromboplastin Time (PTT) or activated Partial Thromboplastin Time (aPTT) ≤1.5 x ULN.
12. Adequate renal function: estimated creatinine clearance ≥30 mL/min according to the Cockcroft-Gault formula or serum creatinine ≤1.5 x ULN.
13. Adequate cardiac function: left ventricular ejection fraction (LVEF) ≥50% at baseline as determined by either echocardiogram (ECHO) or multigated acquisition (MUGA) scan within 28 days before inclusion.
14. Male and female subjects of reproductive/childbearing potential must agree to use of a highly effective contraception for subjects throughout the study and for at least 7 months for females and 4 months for males after last study treatment administration if the risk of conception exists.
15. Women of childbearing potential must have a negative serum pregnancy test within 14 days of enrolment or urine pregnancy test 72 hours prior to enrolment.
16. Patient is willing to comply with 2 sequential tumor biopsies (baseline and at first progression), and with a series of blood samples throughout the study.
17. Patients must be affiliated to a Social Security System.

4.2. Non-inclusion criteria

1. Patient with a breast cancer amenable for resection or radiation therapy with curative intent.

2. Patient has spinal cord compression or clinically active central nervous system metastases, defined as untreated and symptomatic, or requiring therapy with corticosteroids or anticonvulsants to control associated symptoms. Subjects with clinically inactive brain metastases may be included in the study. Subjects with treated brain metastases that are no longer symptomatic and who require no treatment with corticosteroids or anticonvulsants may be included in the study if they have recovered from the acute toxic effect of radiotherapy.
3. Patient with bone metastatic disease only.
4. Patient with multiple primary malignancies within 3 years, except adequately resected non-melanoma skin cancer, curatively treated in-situ disease, other solid tumors curatively treated, or contralateral breast cancer.
5. Persistent unresolved toxicities with grade ≥2 (except alopecia and renal function). Subject with chronic Grade 2 toxicities may be eligible per the discretion of the Investigator after consultation with the Sponsor (eg, Grade 2 chemotherapy-induced neuropathy).
6. Patients receiving treatments such as anti-cancer chemotherapy (non-antibody based immunotherapy), retinoid therapy, hormonal therapy) within 3 weeks before inclusion (within 2 weeks or 5 half-lives, whichever is longer, for small-molecule targeted agents, within 6 weeks for nitrosureas or mitomycin C), anti-body-based anti-cancer therapy within 4 weeks before inclusion, radiotherapy including palliative stereotactic radiation to chest within 4 weeks (palliative stereotactic radiotherapy to other areas: within 2 weeks), chloroquine/hydroxychloroquine within 14 days or major surgery within 4 weeks. Participation in other studies involving investigational drug(s) within 4 weeks prior to study entry and/or during study participation.
7. *Deleted Criterion (Amendment n°4)*.
8. Patients with a concomitant use of chronic systemic (IV or oral) corticosteroids or other immunosuppressive medications except for managing adverse events (inhaled steroids or intra articular steroid injections are permitted in this study.) Subjects with bronchopulmonary disorders who require intermittent use of bronchodilators (such as albuterol) will not be excluded from this study.
9. Patient with history of (noninfectious) interstitial lung disease (ILD)/pneumonitis that required steroids, has current ILD/pneumonitis, or suspected ILD/pneumonitis that cannot be ruled out by imaging at screening.
10. *Deleted criterion (amendment N°7)*
11. Known prior severe hypersensitivity to investigational product or any component in its formulation, including known severe hypersensitivity reactions to study drug (NCI CTCAE v5.0 Grade ≥3).
12. Patient has a history of severe hypersensitivity reactions to other monoclonal antibodies.

13. Patient previously treated with topoisomerase 1 inhibitor.
14. Patient has substance abuse history or any other medical conditions that would increase the safety risk to the subject or interfere with participation of the subject or evaluation of the clinical study in the opinion of the Investigator.
15. Known history of testing positive for HIV or known acquired immunodeficiency syndrome.
16. Known active hepatitis B virus or hepatitis C virus infection at screening. Patients positive for hepatitis C (HCV) antibody are eligible only if polymerase chain reaction is negative for HCV RNA.
17. Active infection requiring systemic therapy (as IV antibiotics, antivirals, antifungals,…).
18. Other severe acute or chronic medical conditions or psychiatric conditions including recent (within the past year) or active suicidal ideation or behavior; or laboratory abnormalities that may increase the risk associated with study participation or study treatment administration or may interfere with the interpretation of study results and, in the judgment of the investigator, would make the patient inappropriate for entry into this study.
19. Patient with a history of symptomatic congestive heart failure (New York Heart association Class II to IV), serious cardiac arrhythmia requiring treatment, history of myocardial infarction or troponin levels consistent with myocardial infarction as defined 28 days prior to randomization., or unstable angina within 6 months prior to inclusion, or current dyspnoea at rest due to advanced malignancy. Patients with troponin levels above ULN at screening, and without any myocardial Infarction related symptoms should have a cardiologic consultation before enrollment to rule out myocardial infarction.
20. Patient with a corrected QT interval (QTc) prolongation to >470 ms (females) or >450 ms (males) based on average of the screening triplicate12-lead ECG.
21. Pregnant women, women who are likely to become pregnant or are breastfeeding or women who want donate, or retrieve for their own use, ova from the time of screening and throughout the study and for at least 7 months after last study treatment administration.
22. Patient with any psychological, familial, sociological or geographical condition potentially hampering compliance with the study protocol and follow-up schedule; those conditions should be discussed with the patient before registration in the trial.
23. Patient unwilling to participate to the biological investigations.
24. Individual deprived of liberty or placed under legal protection.
25. Patient with:

    - Lung-specific intercurrent clinically significant illnesses including, but not limited to, any underlying pulmonary disorder (i.e. e.g. pulmonary emboli within three months of the study randomization, severe asthma, severe COPD, restrictive lung disease, pleural effusion etc.),
    - or any autoimmune, connective tissue or inflammatory disorders (e.g., Rheumatoid arthritis, Sjogren's, sarcoidosis etc.) where there is documented, or a suspicion of pulmonary involvement at the time of screening.

- or prior pneumonectomy.

5. TRIAL TREATMENTS/INTERVENTIONS

5.1. Description of trial treatments/interventions

Patients having signed the informed consent form will receive the first dose of DS-8201a within 5 days of registration. Patients will be administrated intravenous injection of 5.4 mg/kg on day1 of each 21-day cycles. Patients are eligible for repeated treatment cycles in the absence of disease progression and undue adverse events.

5.2. Acquisition, reception, and storage

The investigational products DS-8201a, provided by Daiichi-Sankyo, will be distributed to the pharmacy at the investigational center by the sponsor in accordance with the current Good Distribution Practices guidelines. The pharmacist of the trial site will receive numbered treatment and will acknowledge receipt of all the shipments by sending to the distributor a form duly completed.

The pharmacist is responsible for a safe and proper handling and storage of the investigational medicinal products at the investigational center. The investigational products must be stored in a locked facility with restricted access to the pharmacist and authorized personnel, and under environmental conditions consistent with the drug manufacturer recommendations (refer to the last version of the DS-8201a IB):

- DS-8201a lyophilized powder must be stored between 2ºC and 8 ºC, protected from light.

Up-to-date temperature logs must be maintained by the pharmacist/investigator to document adequate storage during the trial. These logs must be available at the site during monitoring visits, and at the event of an audit or inspection.

If the storage conditions as indicated above are exceeded (e.g. temperature excursion) the pharmacist/investigator must place the corresponding treatments in quarantine and immediate notify the sponsor who will indicate the procedure to follow. Under no circumstances should these treatments be delivered to trial patients without prior authorization by the sponsor.

5.3. Preparation

Instructions for preparation of DS-8201a lyophilized powder will be provided to the pharmacists.

The drug for IV infusion is prepared by dilution of the required volume of the drug product calculated based on the subject’s body weight in a volume of at least 100 mL. Prepared DS-8201a solution should be used immediately. The preparation will be conducted in accordance with the pharmacy manual provided by the Sponsor. Procedures for proper handling and disposal of anticancer drugs should be followed in compliance with the standard operating procedures of the study site.

5.4. Administration

The investigational drug will be administered as a 5.4 mg/kg IV over 30-90 minutes every 21 days. The initial dose of DS-8201a will be infused for approximately 90 minutes. If there is no infusion related reaction, after the initial dose, the next dose of DS-8201a will be infused for approximately

30 minutes. The patient's weight at baseline will be used to calculate the initial dose. If during the course of treatment the patient's weight changes by ±10% of the baseline weight, the patient's dose will be recalculated based on the patient's updated weight.

5.5. Trial treatments accountability, return and destruction

Treatment observance will be recorded in the eCRF.

The investigator/pharmacist must ensure that the investigational product is administrated only to patients enrolled in this trial. The investigational product must not to be used outside the context of the trial protocol.

The pharmacist or authorized staff must document the receipt, dispensation, and return or destruction of all investigational products received during this trial. Records on investigational products delivery to the center, the inventory at the center, the use by each patient, and the return to the sponsor or destruction by the site must be implemented and maintained by the pharmacist or another appropriately trained individual at the investigational center. The following minimum information must be present: all relevant dates (delivery dates, dispensation, returns, and destruction), quantities, and investigational product batch numbers. Accountability form will be provided by the sponsor to ensure trial treatment accountability.

The pharmacist will implement an accounting of medicinal products dispensed, used, unused, returned by the patients. The accountability of the products returned by patients will be systematically done by the pharmacist of the site.

This process will be monitored by the UNICANCER CRA during the trial. The CRA will check that the accountability documentation has been filled in and signed by the pharmacist before the investigational products, used and unused, are destroyed.

All remaining investigational products, used and unused, shall be collected and returned for destruction. The destruction will take place at the investigator centers under the responsibility of their pharmacist in accordance with national regulatory requirements, and with prior formal agreement from the sponsor. A certificate of destruction, identifying concerned products, will be given to the sponsor.

5.6. Formulation, appearance, packaging, and labelling

DS-8201a is supplied as a sterile lyophilized powder dosage form in a single-use vial (Lyo-DP) to be reconstituted with 5 mL of water for injection to 20 mg/mL. Each amber glass vial contains 100 mg of DS-8201a.

Prior to use, Lyo-DP is reconstituted following the preparation procedures provided to the pharmacists.

5.7. Dose adaptation

The investigator will evaluate which toxicities are attributed to DS-8201a and adjust the dose as recommended below. Two dose reductions will be permitted as shown in Table 1. All dose modifications should be based on the worst preceding toxicity (NCI-CTCAE v5.0; Appendix 5).

Specific criteria for interruption, re-initiation, dose reduction and/or discontinuation of DS-8201a are listed in Appendix 7. All interruptions or modifications must be recorded on the AE and drug administration eCRF.

Table 1. *Dose reduction levels of DS-8201a*

Starting Dose	Dose Level -1	Dose Level -2
5.4 mg/kg	4.4 mg/kg	3.2 mg/kg

Dose modification schedule has been specified in Appendix 7. For Grade 3 or Grade 4 events, monitoring (including local lab tests when appropriate) should be performed at intervals no greater than 7 days until AE is determined to be resolving or subject is discontinued at end of treatment. Prophylactic or supportive treatment for expected toxicities, including management of DS8201a-induced adverse events will be as per investigator discretion and institutional guidelines.

Once the dose of DS-8201a has been reduced because of toxicity, all subsequent cycles should be administered at that lower dose level unless further dose reduction is required. If toxicity continues after 2 dose reductions, then the subject will be withdrawn from study treatment. DS-8201a dose increases are not allowed in the study.

Dose can be interrupted for up to 28 days from the planned date of administration (up to a maximum of 49 days from the last cycle). If dose delay longer than 28 days is required, the patient will be withdrawn from the study.

Treatment cycles for a subject for whom DS-8201a dosing was temporarily withheld for any reason may have future cycles scheduled based on the date of the last DS-8201a dose.

All confirmed or suspected COVID-19 infection events must be recorded in the e-CRF. Please refer to Appendix 9 for additional information on dose modification.

5.8. Patients' discontinuations of treatment

Patients can discontinue the trial treatment for the following reasons:

- Limiting toxicity
- Progressive disease
- Death
- Pregnancy
- Any delay in treatment administration of more than 28 days from the planned date of administration (Cycle Day 50)
- Patients decline further treatment but accept to continue with protocol
- Investigator's decision
- Loss of follow-up

After discontinuing all trial treatment, further treatment is left to the physician's discretion.

Patients who discontinue trial treatment will continue with the follow-up and the protocol-defined procedures, unless they specifically withdraw their consent and indicate that they do not want to perform any further trial-related visits or assessments (for patient withdrawals see Section 3.6).

5.9. Concomitant medications and therapies

All medications (including herbal preparations) and therapies taken by the patients or administered to the patients from the onset of trial and given in addition to the investigational products during the trial are considered as concomitant medications. Any concomitant medication(s) trial will be recorded in the eCRF.

5.9.1. *Authorized concomitant treatments*

Prophylactic or supportive treatment of study-drug induced adverse events will be otherwise as per investigator's discretion and institutional guidelines, such as:

- Antiemetics. Based on the currently available clinical safety data, it is recommended that subjects receive prophylactic antiemetics agents prior to infusion of DS-8201a and on subsequent days. Antiemetics such as 5-hydroxytryptamine receptor (5-HT3) antagonists or neurokinin-1 (NK1) receptor antagonists and/or steroids (e.g. dexamethasone) should be considered and administered in accordance with the prescribing information or institutional guidelines.
- Anticoagulants
- Antidiarrheals
- Antibiotics
- Analgesics
- Hematopoietic growth factors : may be used for prophylaxis or treatment based on the clinical judgment of the investigator.
- Concomitant use of dietary supplements, medications not prescribed by the Investigator, and alternative/complementary treatments is discouraged, but not prohibited

  The posology and duration of administration are at the investigator's discretion, as per standard of care. The concomitant treatment administered, and particularly those related to toxicities, must be documented in the patient's medical file at each study visit.

5.9.2. *Prohibited concomitant treatments*

The following treatments/therapies are prohibited during the trial:

- Other antitumor treatments, including immunotherapy, chemotherapies, hormonal therapies, biological response modifiers, and targeted therapies. Concurrent use for

hormones for non-cancer-related conditions (e.g. insulin for diabetes and hormone replacement therapy) is acceptable.

- Other investigational therapeutic agents.
- Radiotherapy (except for palliative radiation to known metastatic sites as long as it does not affect assessment of response or interrupt treatment for more than the maximum time specified in dose modification section).
- Radiotherapy of the thorax.
- Prolonged use of non-steroidal inflammatory drugs.
- Concomitant use of chronic systemic corticosteroids and other immunosuppressive drugs, except for managing adverse events (inhaled steroids or intra articular steroid injections are permitted in this study.) Subjects with bronchopulmonary disorders who require intermittent use of bronchodilators (such as albuterol) will not be excluded from this study. Corticosteroids use will require the highest precaution in patients with diabetes.
- Drug that may cause QTc prolongations or cardiac arrhythmia. Pimozide (Orap®) and cisapride (Prepulsid®) are strictly contraindicated: they are associated with a major risk of ventricular rhythm disorder.
- Concomitant treatment with chloroquine or hydroxychloroquine. Refer to appendix 9 for further details.

5.9.3. *Restricted products*

Use of tobacco products, e-cigarettes and vaping is strongly discouraged but not prohibited.

5.9.4. *Rescue medications and therapies/Treatment at disease progression*

At disease progression, the treatment will be at the investigator's discretion.

5.9.5. *Contraception and gamete donation during the trial*

Definitions

Females of childbearing potential are those who are not surgically sterile (i.e., bilateral tubal ligation, bilateral oophorectomy, or complete hysterectomy) or post-menopausal (for the definition of post-menopausal see below).

Women will be considered post-menopausal if they have been spontaneous amenorrhea for 12 months without an alternative medical cause *(in questionable cases, a blood sample with simultaneous follicle-stimulating hormone [FSH] > 40 IU/mL and estradiol < 40 pg/mL [< 147 pmol/L] is confirmatory)*. The following age-specific requirements apply:

- Women <50 years of age would be considered post-menopausal if they have been amenorrhea for 12 months or more following cessation of exogenous hormonal treatments and if they have luteinizing hormone and follicle-stimulating hormone levels in the post-menopausal range for the institution or underwent surgical sterilization (bilateral oophorectomy or hysterectomy).
- Women ≥50 years of age would be considered post-menopausal if they have been amenorrhea for 12 months or more following cessation of all exogenous hormonal treatments, had radiation-induced menopause with last menses >1 year ago, had chemotherapy-induced menopause with last menses >1 year ago, or underwent surgical sterilization (bilateral oophorectomy, bilateral salpingectomy or hysterectomy).

Females on hormone replacement therapy (HRT) and whose menopausal status is in doubt will be required to use one of the contraception methods outlined for women of child-bearing potential if they wish to continue their HRT during the study. Otherwise, they must discontinue HRT to allow confirmation of post-menopausal status prior to study enrollment. For most forms of HRT, at least 2-4 weeks will elapse between the cessation of therapy and the blood draw; this interval depends on the type and dosage of HRT. Following confirmation of their post-menopausal status, they can resume use of HRT during the study without use of a contraceptive method.

Acceptable contraception during the trial

- **Female patients of child-bearing potential**

Females of childbearing potential who are sexually active with a non-sterilized male partner must use one or more highly effective method of contraception (See below) during the trial and for 7 months after the last trial treatment/therapy administration. Non-sterilized male partners of a female patient must use male condom plus spermicide throughout this period. Cessation of birth control after this point should be discussed with a responsible physician. Female patients should also refrain from breastfeeding throughout this period.

Highly effective methods of contraception:

- Combined (estrogen and progestogen containing) hormonal contraception associated with inhibition of ovulation:
    - Oral
    - Intravaginal
    - Transdermal
- Progestogen-only hormonal contraception associated with inhibition of ovulation:
    - Oral
    - Injectable
    - Implantable
- Intrauterine device

- Intrauterine hormone-releasing system
- Bilateral tubal occlusion
- Vasectomized partner
- Complete sexual abstinence defined as refraining from heterosexual intercourse during and upon completion of the study and for at least 7 months after the last dose of study drug. Periodic abstinence (calendar, symptothermal, post-ovulation methods) is not an acceptable method of contraception.

➢ **Male patients with a female partner of childbearing potential**

Non-sterilized males who are sexually active with a female partner of childbearing potential must use a male condom plus spermicide during the trial and for 4 months after the last trial treatment administration. Male patients should refrain from sperm donation throughout this period.

Gamete donation

Male subjects must not freeze or donate sperm starting at screening and throughout the study period, and at least 4 months after the final study drug administration. Preservation of sperm should be considered prior to enrolment in this study.

Female subjects must not donate, or retrieve for their own use, ova from the time of screening and throughout the study treatment period, and for at least 7 months after the final study drug.

6. EVALUATION OF TREATMENT AND SAFETY

6.1. Efficacy evaluation

Treatment efficacy will be evaluate by measuring changes in tumor size using contrast-enhanced CT-scan or MRI of the chest, abdomen, and pelvis. Imaging disease assessments should be obtained at least every 6 weeks (+/-7 days) during the first 12 months of treatment phase, and every 12 weeks (+/- 7 days) thereafter. **Imaging disease assessments should be repeated at least 4 weeks after assessment of a PR or CR as per RECIST v1.1 guidelines.**

Radiological assessments have to be repeated in the same technical conditions (same machine, parameters …) and reviewed by the same radiologist.

For patients dropped-out for other reasons than disease progression, tumor evaluation has to be continued during the post-treatment period and documented every 6 weeks (or every 12 weeks after the first 12 months of treatment phase) until disease progression or initiation of an antineoplastic treatment.

6.2. Safety evaluation

Treatment safety evaluation will be based on adverse event (AE) occurrence, the use of concomitant treatments, and changes occurring during treatment including those observed during physical examinations, in vital signs (blood pressure, pulse, body temperature, respiratory rate, oxygen saturation of the blood), in electrocardiogram, and with biological and clinical examinations (biochemistry, hematology). Safety criteria will be evaluated using the NCI-CTCAE v5.0 (Appendix 5).

In case of emergency, the patient, a patient's relative or the patient's general practitioner will have to inform by phone the investigator about the occurrence of an AE. The possible treatment interruption or dose adaptation (decrease) for the investigational product will be considered as well as adequate concomitant treatment if necessary.

6.2.1. *ECG guidelines*

Triplicate ECGs at baseline, and single ECG subsequently (in triplicate if an abnormality is noted) will be performed every 4 cycles. Standard ECG parameters will be measured, including RR, PR, QT intervals, and QRS duration. All ECGs must be evaluated by investigator or delegated physician for the presence of abnormalities. Whether or not measurement is performed, date performed, results, and findings for each parameter will be recorded in the eCRF.

6.2.2. *LVEF guidelines*

LVEF will be measured by either Echo or MUGA scan. All Echos/MUGAs will be evaluated by the investigator or delegated physician for monitoring cardiac function.

6.3. Centralized review

6.3.1. *Radiological review*

Independent radiological reviews will be organized at the interim analysis of the cohort 3 and at the end of the trial for the validation of responses (PR or CR) occurring during treatment in each cohort.

Therefore, each baseline and subsequent radiological tumoral assessment (CT scan or MRI or other method) for every patient in the study must be download into the Aquilab Share Place website in accordance with the technical manual that will be provided by the Sponsor.

6.3.2. *HER2 status review*

HER2 status determination on the baseline biopsy will be centrally performed within the GEFPICS pathologist network (national group of French pathologists experienced in breast pathology). HER2 expression will be determined according to the last American Society of Clinical Oncology - College of American Pathologists guidelines (as of 2018).

The pathologist will provide a report to the physician within 1 month after the patient inclusion. In the situation where HER2-expression is different from the status at inclusion, a reclassification will be operated, and the patient will be switched to the corresponding cohort.

The slides used for the HER2 status review will be centralised at the same time as the frozen or FFPE biopsies.

7. DESCRIPTION OF VISITS AND INVESTIGATIONS

Patients will be monitored from the date of their screening visit until the date of death, withdrawal of consent, loss to follow-up, or end of study, whichever occurs first. A table summarizing the follow-up examination/visit schedule is provided in the section "Schedule of visits and activities" of the protocol summary.

7.1. Baseline visit

Eligible patients with signed informed consent form will have baseline assessments within 28 days before starting study treatment. Patients will begin study treatment within 5 days of registration.

7.1.1. *Within 28 days before treatment initiation*

- **Patient history**
  - Demographic data
  - Cancer history and characteristics/prior therapy
  - Other relevant medical history
- **Safety assessment**
  - Ongoing toxicities or adverse event must be monitored until resolution or returned to baseline level.
  - Troponin (preferably high-sensitivity troponin-T) Patients with troponin levels above ULN at screening, and without any myocardial Infarction related symptoms should have a cardiologic consultation before enrollment to rule out myocardial infarction
  - Cardiologic examination: triplicate ECG 12 leads, LVEF (by echocardiogram or MUGA).
  - Ophthalmologic examination: including visual acuity testing, slit lamp examination and fundoscopy.
- **Tumor evaluation**
  - Clinical disease assessment
  - Radiologic assessment (RECIST v1.1): Bone scan, brain MRI or tomography, TAP-CT scan or MRI. Bone scan can be replaced by PET-CT if the histological type is not lobular.
- **Ancillary studies for all the centers (mandatory for all the patients)**
  - Biopsy of a metastatic site: when possible up to 3 cores (1 frozen, 2 FFPE) will be collected from a metastatic site (or primary when stage IV at diagnosis). When patients already have an archived biopsy from a secondary or a primary site (if stage IV) of their current disease,

this material can be used for the study, provided that: it was collected within 3 months prior enrolment and a frozen and a FFPE sample are both available for research (see Appendix 6).

- Blood sample for circulating tumor DNA (ctDNA) and circulating tumor cells (CTC): 30 mL.
- Whole blood sample for constitutional DNA analysis: 5 mL.

- **Bystander effect study in selected centers**

  The centers that were selected for this research will have to propose to the patient a biopsy 24 hours after treatment initiation (H24 biopsy).

  To facilitate the acceptance of the H24 biopsy, patients can skip the baseline biopsy if they have an archived biopsy from a secondary or a primary site (if stage 4) of their current disease, provided that: a) the sample was collected less than 1 year from the inclusion date; b) at least 1 FFPE is available; c) the patient can undergo a H24 biopsy in the same organ as the archived samples.

7.1.2. *Within 14 days before treatment initiation*

- **Collection of concomitant treatments received within 2 weeks of enrolment. Check the current administration of prohibited medications and start considering substituting them**
- **Safety assessment**
  - Clinical examination: physical examination, height, weight, ECOG PS, vital signs.
  - Biological tests (Any result outside the normal range or inclusion range will be repeated, at the discretion of the investigator).
    - ✓ Hematological: Red blood cell count, hemoglobin, hematocrit, platelet count, white blood cell count, differential white blood cell count (neutrophils, lymphocytes, monocytes, eosinophils, basophils).
    - ✓ Coagulation parameters: PTT/Activated partial thromboplastin time (APTT) or partial thromboplastin Time (PTT) and international normalized ratio (INR).
    - ✓ Biochemistry
      - ❖ Alkaline phosphatase, ALT (SGPT), AST (SGOT), and total bilirubin: These tests must be conducted and assessed concurrently. If total bilirubin is ≥2 × ULN (and no evidence of Gilbert's syndrome) then fractionate into direct and indirect bilirubin.
      - ❖ Albumin, calcium, chloride, serum creatinine, lactate dehydrogenase (LDH), magnesium, potassium, sodium, total protein, and urea or blood urea nitrogen depending on local practice.
  - Pregnancy test (for female pre-menopausal patients only): Serum pregnancy test within 14 days before enrolment or urine test within 72 hours of enrolment.

7.2. Visits and assessment during treatment period

- **Collection of concomitant treatments: day (D) 1 of every treatment cycle (C).**
- **Safety assessment**
  - Ongoing toxicities or adverse event must be monitored until resolution or returned to baseline level.
  - Clinical examination (D1 of every treatment cycle): Physical examination, weight, ECOG PS, vital signs.
  - Cardiologic examination:
    - ✓ Single ECG 12 leads every 4 cycles (triplicate if an abnormality is noted).
    - ✓ LVEF: On D1C5 and then every 4 cycles (e.g. Cycle 5, 9, 13,…). The same method (echocardiogram or MUGA) should be used throughout the study.
  - At any time, if clinically indicated: ophthalmologic examination including visual acuity testing, slit lamp examination and fundoscopy.
  - Biological tests (Any result outside the normal range or inclusion range will be repeated, at the discretion of the investigator): D1, D8, D15 of C1, then D1 of subsequent cycles.
    - ✓ Hematological: Red blood cell count, hemoglobin, hematocrit, platelet count, white blood cell count, differential white blood cell count (neutrophils, lymphocytes, monocytes, eosinophils, basophils).
    - ✓ Biochemistry
      - ❖ Alkaline phosphatase, ALT (SGPT), AST (SGOT), total bilirubin: These tests must be conducted and assessed concurrently. If total bilirubin is ≥2 × ULN (and no evidence of Gilbert's syndrome) then fractionate into direct and indirect bilirubin.
      - ❖ Albumin, calcium, chloride, serum creatinine, LDH, magnesium, potassium, sodium, total protein, and urea or blood urea nitrogen depending on local practice.

- **Tumor evaluation every 6 weeks (±7 days) starting at D1C3 during the first 12 months of treatment, then every 12 weeks (±7 days)**
  - Clinical disease assessment
  - Radiologic assessment (RECIST v1.1)
    - ✓ TAP-CT scan or MRI. According to RECIST v1.1, radiologic assessment must be repeated at least 4 weeks after observation of a CR or PR.
    - ✓ Bone scan, brain MRI or tomography: In case of known bone metastases, known CNS metastases, or if clinically indicated. Bone scan can be replaced by PET-CT if the histological type is not lobular and if a PET-CT has been achieved at baseline.

- **Ancillary studies for all the centers :**
  - mandatory for all the patients
    - ✓ Blood sample for ctDNA and CTC: 30 mL will be collected on D1C2, D1C3, D1C5, and D1C7.
  - mandatory for HER2 overexpressed patients (cohort 1)
    - ✓ For patients with a HER2 3+ or HER2 2+/ISH+ status: a biopsy of a metastatic site will be performed at 6 weeks (+/- 1 week) from treatment initiation. Up to 2 cores (1 frozen, 1 FFPE) will be collected on the same lesion as baseline sample.
  - optional for patients with HER2 low or null status (cohort 2 and 3)
    - ✓ For patients with a HER2 2+/ISH- or HER2 1+ or HER2 0+ status: a biopsy of a metastatic site will be performed at 6 weeks (+/- 1 week) from treatment initiation. Up to 2 cores (1 frozen, 1 FFPE) will be collected on the same lesion as baseline sample.
- **Bystander effect study for selected centers**
- Up to 2 cores (1 frozen, 1 FFPE) will be collected at 24h after treatment initiation (D2C1) on the same organ as baseline. When H24 biopsy is very close to the previous one (less than 3 weeks), it must be done on the same organ but not on the same lesion.
- This biopsy is optional for the patient. However, if the baseline biopsy was skipped to the favor of an archived biopsy, then the H24 biopsy should be considered as the first biopsy done for the study. In this situation, the investigator shall do all his/her effort to explain the patient that a biopsy is required at study entry, should it be at baseline or at H24.
- The patient who undergo a H24 biopsy will not be proposed the 6 weeks biopsy, regardless of the HER2 status.

7.3. End-of-treatment visit (6 weeks after last DS-8201a administration)

- **Collection of concomitant treatments.**
- **Safety assessment**
  - Ongoing toxicities or adverse event must be monitored until resolution or returned to baseline level.
  - Clinical examination: Physical examination, weight, ECOG PS, vital signs.
  - Cardiologic examination: LVEF. The same method (echocardiogram or MUGA) should be used throughout the study. Single ECG 12 leads. Triplicate ECG 12 leads if an abnormality is noted.
  - Ophthalmologic examination: including visual acuity testing, slit lamp examination and fundoscopy.

- Biological tests (Any result outside the normal range or inclusion range will be repeated, at the discretion of the investigator.
  - ✓ Hematological: Red blood cell count, hemoglobin, hematocrit, platelet count, white blood cell count, differential white blood cell count (neutrophils, lymphocytes, monocytes , eosinophils, basophils).
  - ✓ Biochemistry
    - ❖ Alkaline phosphatase, ALT (SGPT), AST (SGOT), total bilirubin: These tests must be conducted and assessed concurrently. If total bilirubin is ≥2 × ULN (and no evidence of Gilbert's syndrome) then fractionate into direct and indirect bilirubin.
    - ❖ Albumin, calcium, chloride, serum creatinine, LDH, magnesium, potassium, sodium, total protein, and urea or blood urea nitrogen depending on local practice.
  - ✓ Urine or serum pregnancy test (for female pre-menopausal patients only). If urine test is positive, confirm with a serum test.

- **Tumor evaluation**
  - Clinical disease assessment.
  - Radiologic assessment (RECIST v1.1)
    - ✓ Bone scan, brain MRI or tomography: In case of known bone metastases, known CNS metastases, or if clinically indicated.
    - ✓ TAP-CT scan or MRI. Assessments should not be repeated if they were obtained less than 6 weeks from withdrawal of therapy

- **Ancillary studies for all the centers (mandatory for the patients)**
  - Biopsy of a metastatic site: when possible up to 3 cores (1 frozen, 2 FFPE) will be collected from a metastatic site (or primary when stage IV at diagnosis) at disease progression.
  - Blood sample for ctDNA and CTC: 30 mL.

7.4. Follow-up (every 12 weeks and up to 3 years)

- **Collection of first post-study new anti-neoplastic treatments.**
- **Safety assessment:**
  - Ongoing toxicities or adverse event must be monitored until resolution or returned to baseline level.
- Urine or serum pregnancy test (for female pre-menopausal patients only) during the 2nd follow-up visit at 6 months. If urine test is positive, confirm with a serum test.
- **Disease and survival status**

- Disease status should be collected once and corresponds to the date of the first progression observed after study treatment discontinuation, when occurred for other reasons than progression.

- **Tumor evaluation** : **has to be continued during the post-treatment period if withdrawal was not related to disease progression and should be continued and documented every 6 weeks (or every 12 weeks after the first 12 months of treatment phase) until disease progression or initiation of an antineoplastic treatment**
  - Clinical disease assessment.
  - Radiologic assessment (RECIST v1.1)
    - ✓ Bone scan, brain MRI or tomography: In case of known bone metastases, known NCS metastases, or if clinically indicated.
    - ✓ TAP-CT scan or MRI

7.5. Provisions in case of treatment or trial interruption

In case of treatment or study discontinuation, the patient will have to be followed as indicated in sections 3.5 and 3.6. However, further treatment will be at the investigator's discretion as per standard of care.

8. REPORTING OF ADVERSE EVENTS

8.1. Adverse event: general definition

An adverse event (AE) is defined as any untoward medical occurrence, in a patient or clinical trial subject treated by a medicinal product and which does not necessarily have a causal relationship with this treatment.

8.2. Serious adverse event: general definition

A serious adverse event (SAE) is defined as any untoward medical occurrence that at any dose:

- Results in death
- Is life-threatening
- Requires hospitalization or prolongation of existing hospitalization
- Results in persistent or significant disability/incapacity
- Is a congenital anomaly/birth defect
- Is medically relevant in the context of the pathology and the clinical trial

These characteristics/consequences are to be considered at the time of the event. For example, regarding a life-threatening event, this refers to an event in which the subject was at risk of death at the time of the event; it does not refer to an event which hypothetically might have caused death if it was more severe.

The terms disability and incapacity correspond to any clinically relevant physical or psychological handicap, transient or permanent, which impact the patient's physical condition/activity and/or the quality of life.

Medical and scientific judgment should be exercised in deciding whether other situations should be considered serious, such as important medical events that might not be immediately life-threatening or result in death or hospitalization, but might jeopardize the patient or might require intervention to prevent one of the other outcomes listed in the definition above (for example: overdose, second cancer, etc.).

Any SAEs directly related to tissue screening procedure (ie, tumor biopsy) must be reported.

Second cancer, whether or not related to the research, must be considered medically relevant and reported to the R&D UNICANCER pharmacovigilance unit without any limitation in terms of deadline.

The investigator will assess whether a reasonable causal relationship exists between the event and the treatment/therapy. If the sponsor disagrees with the investigator's causality assessment, the opinion of both the investigator and the sponsor will be reported.

The events related to disease progression are considered as SAEs if they are associated to a seriousness criterion but should not be managed according to the section 8.3. These events do not require immediate reporting and should be reported only in the case report form.

The following events leading to a hospitalization or a prolongation of hospitalization **are not considered as Serious Adverse Events**:

- Hospitalization already scheduled before the start of the trial,
- Hospitalization required as part of the protocol (biopsy, chemotherapy, etc)

A Suspected Unexpected Serious Adverse Reaction (SUSAR) is defined as any serious adverse reaction, the nature, severity or outcome is not consistent with the applicable drug information (e.g. IB for an unapproved investigational product or package insert/SmPC for an approved product).

The reference document for the assessment of expected/unexpected serious adverse drug reaction is the current version of Investigator's Brochure of the study treatment «DS-8201a ».

The assessment of expected/unexpected character of the event is the responsibility of the sponsor.

New event: Defined as any new event relating to the conduct of the trial or the development of the investigational medicinal product where that new event is likely to affect the safety of the subjects.
Severity criterion: The severity criterion must not be confused with the seriousness criterion which is the guide for defining the reporting requirements.

The intensity (severity) of events will be estimated using the extract of NCI-CTCAE v5.0 classification (Appendix 5). The intensity of adverse events not listed in this classification will be assessed according to the following qualifiers:

- **Grade 1 (mild):** Asymptomatic or mild symptoms; clinical or diagnostic observations only; intervention not indicated.
- **Grade 2 (moderate):** Minimal local or noninvasive intervention indicated; limiting age-appropriate instrumental ADL.
- **Grade 3 (Severe):** or medically significant but not immediately life-threatening; hospitalization or prolongation of hospitalization indicated; disabling; limiting self-care ADL.
- **Grade 4 (Life-threatening):** consequences; urgent intervention indicated.
- **Grade 5 (Death):** related to the event.

8.3. Adverse Event of Special Interest (AESI)

8.3.1. *Cardiac Related Events including LV dysfunction*

LV dysfunction in association with DS-8201a is considered to be important potential risks based on the available pre-clinical data, literature and available safety information for drugs of similar class. Refer to the current IB for a summary of preliminary clinical trial data.

Either Echo or MUGA scan will be used to measure LVEF. All Echo/MUGAs, will be evaluated by the investigator or delegated physician for monitoring cardiac function. Troponin will be measured at baseline, end of treatment and as needed based on subject reported cardiac symptoms suggesting congestive heart failure, myocardial infarction or others causes of cardiac myocyte necrosis.

Triplicate ECG will be performed at screening and then single ECG every 4 cycles as defined per schedule of assessments (in triplicate if an abnormality is noted). Standard ECG parameters will be measured, including R-R, P-R, QTc intervals, and QRS duration. All ECGs must be evaluated by investigator or delegated physician for the presence of abnormalities. Whether or not measurement is performed, date performed, results, and findings for each parameter will be recorded in the eCRF.

8.3.2. *Interstitial Lung Disease/Pneumonitis*

Interstitial lung disease/pneumonitis is considered an important identified risk based on a comprehensive cumulative review of the available safety data from the clinical development program. Refer to the current IB for a summary of preliminary clinical study data.

Management Guidance:
ILD/pneumonitis should be ruled out if a subject develops radiographic changes potentially consistent with ILD or develops an acute onset of new or worsening pulmonary or other related signs/symptoms such as dyspnea, cough or fever. If the AE is confirmed to have an etiology other than ILD/pneumonitis, follow the management guidance outlined in the designated “Other Non-Laboratory Adverse Events” dose modification section of the Appendix 7.
If the AE is suspected to be ILD/pneumonitis, treatment with study drug should be interrupted pending further diagnostic evaluations. Evaluations should include high resolution CT, pulmonologist consultation (infectious disease consultation as clinically indicated), blood culture and CBC (other blood tests could be considered as needed), bronchoscopy and bronchoalveolar lavage if clinically indicated and feasible should be considered, pulmonary function tests and pulse oximetry (SpO2), and arterial blood gases if clinically indicated.

If the AE is confirmed to be ILD/pneumonitis, follow the management guidance outlined in the designated “Pulmonary Toxicity” dose modification section of the appendix 7.

All events of ILD regardless of severity or seriousness will be followed until resolution including after drug discontinuation.

8.4. Measures to be taken in case of a serious adverse event

The investigator ensures that adequate medical care is provided to the patient. Treatment of the event may require decoding of the investigational medicinal product.

The investigator must immediately following knowledge of the event, notifies the R&D UNICANCER pharmacovigilance unit of any SAE or any new event defined here above, whether or not related to the research, which occurs during the ‘trial reporting period’. This reporting period:

- Starts at the date of the signature of the informed consent form.
- Covers the entire period during which the patient is receiving the investigational treatment or is subject to specific procedures related to the trial.
- Covers a period of 47 days after the last administration of the investigational product.

Any later SAE, i.e. occurring after a period of 47 days, which is considered to be related to the experimental treatment(s) or to the research (other treatment used, diagnostic procedures and examinations carried out during the research) must be reported without any limitation in terms of deadline.

Notification must be carried out immediately by fax to the R&D UNICANCER pharmacovigilance unit by sending the form “notification of a SAE”, located in the Investigator Master File, completed as precisely as possible, dated and signed by the physician-investigator:

R&D UNICANCER

Pharmacovigilance unit, France

Phone: +33 (0)1 44 23 04 16 – Fax: +33 (0)1 44 23 55 70

Email: pv-rd@unicancer.fr

Abnormal laboratory results should be reported as SAE if they possibly put at risk the patient or they require medical intervention to prevent an outcome corresponding to one of severity criteria.

The investigator **shall send additional information to the R&D UNICANCER pharmacovigilance unit** using a SAE declaration form (by ticking the Follow-up X box to specify that it is a follow-up and not an initial report) as soon as he is aware of the event. The investigator must also submit the last follow-up at the resolution or stabilization of the SAE.

The investigator is responsible for appropriate medical follow-up of patients until the resolution or stabilization of the event or until the death of the patient. This can sometimes mean that the follow-up continues after the patient has left the trial.

The investigator must keep the documents concerning the suspected SAE in order to supplement the information previously submitted if necessary.

Requests for clarification and additional information may be sent to the investigator by the R&D UNICANCER pharmacovigilance unit or CRA sponsor of the trial to document and treat the case.

The physician-investigator should also attach to the form «notification of a SAE», whenever possible:

- a copy of the hospital report or extended hospitalization report
- a copy of all results of additional investigations carried out, including also relevant negative results, and enclosing the normal laboratory values
- a copy of the autopsy report if necessary
- any other document deemed to be useful and pertinent

All these documents must be anonymized.

In the event of pregnancies:

The occurrence of pregnancy is not considered as a SAE. If, however a female study patient becomes pregnant or is discovered to have been pregnant during the treatment period or within 7 months after the last dose of DS-8201a, the investigator must immediately notify the Sponsor of this event via the Pregnancy Notification Form in accordance with SAE reporting procedures.

The investigator should discontinue study treatment and counsel the patient, discussing the risks of the pregnancy and the possible effects on the fetus. Monitoring of the patient should continue until conclusion of the pregnancy.

Follow-up information regarding the course of the pregnancy, including perinatal and neonatal outcome and, where applicable, offspring information must be reported on the Pregnancy Notification Form.

While pregnancy is not considered as a SAE, any anomaly detected in the fetus or child, any elective termination of a pregnancy for medical reasons, or spontaneous abortion will be reported as a SAE.

9. TRANSLATIONAL STUDY

All patients enrolled in this study will to participate in the translational study. In addition, some patients will be solicited for additional optional translational study. Patients wanting to participate in the optional translational research will sign a separate informed consent forms. The collection and use of the patient’s biological samples will not modify or impact the diagnosis, medical care, and treatment administered to the patient.

The following biological samples will be collected:

- Relevant frozen and FFPE tumor samples (mandatory) of biopsy of metastasis will be collected at baseline and tumor progression.
- Additional tumor sample of the same site will be collected at 6 weeks post-treatment initiation : mandatory for cohort 1 patients and optional for cohort 2 and 3 patients
- Additional tumor sample of the same site will be collected at 24h post-treatment initiation, only in selected centers.
- Blood samples (mandatory) will be collected at baseline, after 1 cycle (D1C2), after 2 cycles (D1C3), after 4 cycles (D1C5), after 6 cycles (D1C7) and at the end of treatment (whatever the reason).

All the samples will be transported and centralized at "Centre des Ressources Biologiques d'UNICANCER", under the responsibility of Ms. Séverine Tablone-Eglinger, located at the following address:

Centre Léon Bérard, 28 rue Laënnec

69373 Lyon cedex 08, Bâtiment CHENEY B Rez de Chaussée

CRB échantillons biologiques - France

The tissue and blood samples collected will be used to identify biomarkers that may be predictive of response or toxicity to the proposed treatments and/or prognostic for breast cancer. The collected tumor tissue and blood samples may also be used to develop and validate diagnostic assays and allow the generation of statistically meaningful biomarker data. A detailed description of the translational study is provided in Appendix 6.

10. DESCRIPTION OF STATISTICAL METHODS

The first statistical analysis plan corresponds to the statistical analysis presented in this protocol. The SAP may be enriched and detailed before any statistical analysis, and may be revised as well during the course of the trial in case of substantial modification of the protocol or following recommendations of the Independent Data Monitoring Committee (IDMC). Any revision of the SAP will be validated by the Steering Committee.

10.1. Statistical hypothesis and sample size determination

As described in sections 10.1.1, 10.1.2 and 10.1.3, 162 patients have to be included in three cohorts to answer the main question of this study:

- Cohort 1 : 74 patients HER2 IHC3+ or IHC2+/ISH+

- Cohort 2 : 44 patients HER2 IHC1+ or IHC2+/ISH-
- Cohort 3 : 44 patients HER2 IHC0+

However, since the HER2 status is confirmed after inclusion, some switches may occur after inclusion from the initially allocated cohort to the confirmed HER2 status cohort.

Considering that cohort 2 will be the first to be terminated due to the higher frequency of HER2 IHC1+ or 2+/ISH- scores, we thus estimate that there will be around 25% of switches from cohort 1 and 3 to cohort 2, until they will be completed.

In order to reach the expected cohorts sample size, we estimate that around 30 additional patients may be necessary at the most to achieve the expected recruitment in cohort 1 and 3.

10.1.1. *Cohort 1: HER2 IHC3+ or IHC2+/ISH+*

The primary endpoint of the phase II is the rate of patients presenting best objective response. The following hypotheses are used for cohort 1:

- p0 = 30%, maximal unacceptable rate of patient presenting a best objective response for whom the experimental treatment will be considered as insufficiently active.
- p1 = 45%, minimal acceptable rate of patients presenting a best objective response for whom the experimental treatment will be considered as sufficiently active.

Using an A'Hern design (25) (alpha = 5%, 1-beta = 80%) and (p0 = 30%; p1 = 45%), 67 evaluable patients need to be included in the study. The decision rules are summarized in Table 2.

Table 2. *Number of patients required in the HER2 IHC3+ or IHC2+/ISH+ cohort*

Nb of patients	Insufficiently active	Sufficiently active
67 evaluable/ 74included	<27 successes	≥27 successes

Assuming a rate of around 10% of non-evaluable patients for the first assessment, to reach 67 evaluable patients, 67/90% = 74 patients HER2 IHC3+ or IHC2+/ISH+ have to be included.

10.1.2. *Cohort 2 HER2 IHC1+ or IHC2+/ISH-*

The primary endpoint of the phase II is the rate of patients presenting a best objective response. The following hypotheses are used for cohort 2:

- p0 = 20%, maximal unacceptable rate of patients presenting a best objective response for whom the experimental treatment will be considered as insufficiently active.
- p1 = 40%, minimal acceptable rate of patients presenting a best objective response for whom the experimental treatment will be considered as sufficiently active.

Using an A'Hern design (25) (alpha = 5%, 1-beta = 85%) and (p0 = 20%; p1 = 40%), 40 evaluable patients need to be included in the study. The decision rules are summarized in Table 3.

Table 3. *Number of patients required in the HER2 IHC1+ or IHC2+/ISH- cohort*

Nb of patients	Insufficiently active	Sufficiently active
40 evaluable/ 44 included	<13 successes	≥13 successes

Assuming a rate of around 10% of non-evaluable patients for the first assessment, to reach 40 evaluable patients, 40/90% = 44 patients HER2 1+ or 2+ have to be included.

10.1.3. *Cohort 3: HER2 IHC0+*

The primary endpoint is the rate of patients presenting a success. A success is defined as a patient presenting an objective response.

The short-term endpoint is the rate of patient without progression at 3 months.

The following hypotheses are used:

- Primary endpoint:

  - p10=20%, maximal unacceptable rate of patients presenting a success for whom the experimental treatment will be considered as insufficiently active.
  - p11=40%, minimal acceptable rate of patients presenting a success for whom the experimental treatment will be considered as sufficiently active.

- Short-term endpoint:

  - p20=30%, maximal unacceptable rate of patients without progression at 3 months for whom the experimental treatment will be considered as insufficiently active.
  - p21=50%, minimal acceptable rate of patients without progression at 3 months for whom the experimental treatment will be considered as sufficiently active.

Using a Kunz design (Kunz et al., SMMR 2017) with (alpha=5%, 1-beta=85%) and (p10=20%, p11=40%, p20=30% and p21=50%), 40 patients need to be included in the study and an interim analysis will be performed after inclusion of 16 patients. The decision rules are summarized in the table below:

Table: Number of patients required in the HER2 IHC0+ cohort

Step	Nb of patients	Insufficiently active	Sufficiently active
1	16	≤ 4 non-progression	
2	40 evaluable	≤ 12 successes	≥ 13 successes

Assuming a rate of around 10% of non-evaluable patients for the first assessment, to reach 40 evaluable patients, 40/0.9 = **44** patients HER2 0+ have to be included in the cohort 3

10.2. Trial populations to be analyzed

- ✓ Full Analysis Set: All patients who received at least one dose of study drug and who had a valid first post-baseline assessment of disease status (or who had progressive disease).
- ✓ Safety population: The safety population will include all patients who received the study drug at least once.

10.3. Planned statistical analysis

10.3.1. *Demographic data*

Continuous variables will be summarized by cohort using median, minimum, maximum and number of available observations. Qualitative variables will be summarized by cohort using: counts, percentages, number of missing data.

10.3.2. *Primary endpoint*

The primary endpoint will be analyzed on the FAS population. The primary endpoint of the phase II is the rate of patients presenting a best objective response under treatment, as assessed by the investigator. It will be assessed and described by cohort using frequency, percentage and 95% confidence intervals (Binomial exact).

Short term endpoint for cohort 3 (IHC0+) population will be analyzed on the FAS population. The short term endpoint of the cohort IHC0+ is the rate of patients presenting a non- progressive disease at 3 months according to RECIST v1.1 (local evaluation). It will be assessed and described using frequency, percentage and 95% confidence intervals (Binomial exact).

The recruitment will be interrupted in cohort 3 according to the methodology after the inclusion of 16 patients to perform the pre-planned interim analysis. The recruitment in the cohort will be resumed or definitely interrupted upon IDMC decision.

10.3.3. *Secondary endpoint*

- ➢ Secondary efficacy endpoints will be analyzed on the FAS population:

- For each cohort, survival rates (PFS, OS, and duration of response) will be estimated at different time points using the Kaplan-Meier method (with their respective confidence interval). Median survival times will be estimated by cohort with corresponding 95% confidence interval.
- Clinical benefit rates will be assessed and described by cohort using frequency, percentage, and 95% confidence intervals (Binomial exact).

- Safety analysis will be performed on the safety population, by cohort and on the overall population. Each of the following will be assessed:
  - AEs by toxicity grade
  - SAEs
  - Investigated drug-related AE
  - AE leading to interruption or dose reduction
  - Occurrence of AE with toxicity grade >2

  Frequency, percentage with 95% confidence interval will be computed for each event.

10.3.4. *Modifications of SAP and initial statistical analysis strategy*

Any modification/change made to the initial statistical analysis plan (SAP) will be described in details, well-argued and commented in an updated version of the SAP. These modifications may be complementary/exploratory analyses not envisaged initially.

11. OVERSIGHT COMMITTEES

11.1. Independent Data Monitoring Committee (IDMC)

An IDMC, with expertise and experience in the pathology, and without direct involvement in the conduct of the trial, will be set up specifically to guarantee:

- Effective protection of patients,
- Insure the ethical conduct of the trial,
- Benefit/risk ratio of the trial,
- Ensure the independent review of the scientific results during the trial and at the end of the trial.

The IDMC will meet at least when the 30 first patients will be included in the cohorts 1 and 2 for an interim safety analysis.

Another meeting is planned when the 16 first patients will be included in the cohort 3 for the pre-planned specific interim safety analysis.

The IDMC members will then establish the appropriate follow-up of the trial and the frequency of the IDMC meeting throughout the study conduct. The IDMC will be composed of at least:

- Two oncologists

- A statistician
- A pathologist

Data presented to IDMC are strictly confidential.

The IDMC may recommend the early termination of a cohort of or the trial if one of the following conditions is met:

- The results of the interim safety analysis.
- An unacceptable toxicity.
- Data available from the trial or any other source of information are sufficiently convincing to influence the therapeutic practice of the majority of clinicians.

The IDMC has only a consultative role; it will inform the sponsor who will decide whether the IDMC recommendation will be followed.

11.2. Steering Executive Committee

A Steering executive committee has been implemented for this trial. The Steering executive committee will meet physically (or at least through a teleconference) in a regular basis (at least twice a year) to discuss key questions related to the protocol (see examples listed below). Additional meetings may be organized, as required.

The Steering executive committee is composed of (non-exhaustive list):

- ➢ The principal investigator
- ➢ UNICANCER project manager
- ➢ The study statistician
- ➢ The referent pathologist
- ➢ Some investigators involved in the study
- ➢ At least one biologist

The steering committee will be responsible for:

- Approving the protocol and any amendments thereof.
- Maintaining the scientific integrity of the Study, according to the protocol.
- Ensuring the trial is conducted in an ethical manner; reviewing recommendations of the IDMC and determining actions to be taken as applicable in order to ensure the safety of the participants.
- Reviewing the recruitment rate and any kind of issues raised during the conduct of the study to adjust the protocol conditions via a protocol amendment, if needed.
- Reviewing and deciding on Research Projects requiring access to and use of Study Data and/or Biological Samples.
- Publications and presentations of the Study Data.

The Steering executive committee will assist UNICANCER in resolving issues and/or questions encountered during the trial and will consider with UNICANCER changes to the protocol as necessary.

12. QUALITY ASSURANCE

12.1. Data collection

All data necessary for the research must be entered into the study eCRFs in a timely manner. eCRFs will be completed by the principle investigator and other staff members duly designated. The data entered must be accurate and complete.

The study database will be hosted by:

Institut du Cancer Montpellier (ICM) – Val d'Aurelle

Unité de Biométrie – CTD INCa

208 rue des Apothicaires - Parc Euromédecine

34298 Montpellier Cedex 5 – France

Mrs Sophie Gourgou will be responsible for the trial database.

Database management will be provided by eCRF developed using the CSOnline module of Ennov Clinical® software. In case of technical problem with the eCRF, the investigator may refer to the specific operating procedure of the eCRF or directly contact:

ICM – Unité de Biométrie – CTD INCa

Data center UNICANCER

from Monday to Friday 9 am-5 pm

email: support.ecrf@icm.unicancer.fr

Fax: +33 (0)4 67 61 37 18

Tel: +33 (0)4 67 61 45 48/24 52

The access code (login) and passwords to access the eCRF will be sent directly to each users personal email account. The logins and personal passwords to connect to the eCRF, via the website - https://ecrf.icm.unicancer.fr/CSOnline - will automatically be generated by CSOnline.

A password non-disclosure certificate will be signed by the principal investigator of each center engaging his/her responsibility regarding the confidentiality of the access codes for all users of the eCRF at their center.

Trial data will be entered directly by the principle investigator or by designated staff members of each center, via the eCRF, and will be controlled and validated according to the standard procedures (included those in the software and the sponsor's quality assurance procedures). When using the

eCRF, traceability of access and changes made to the eCRF are traced by the software (audit trail). At the end of the trial and once all the eCRF data are validated, the investigator will login to the eCRF to sign all the pages to validate the data entered for each patient.

The sponsor will create and send an electronic copy (PDF file) of each patient's CRF to the corresponding investigator. This pdf file must be printed and signed by the investigator, and then archived at the investigator's site.

12.2. Access to data

The sponsor has direct access to all investigator sites, original records, source data/document and reports to allow quality control and auditing by the sponsor or on behalf of the sponsor.

Investigators will make available to the authorized persons the documents and the patients' individual data that are essential to monitor the trial on an ongoing basis, to perform quality control and audit of this research in accordance with national regulatory requirements.

12.3. Study monitoring

To ensure the authenticity and credibility of data in accordance with the current ICH Harmonized Tripartite Guideline for Good Clinical Practice (ICH-GCP), the sponsor has established a quality assurance system that consists of:

- The management and the monitoring of the trial according to UNICANCER procedures. The monitoring strategy is built according to a systematic, prioritized, risk-based approach, and is documented in the monitoring plan.
- The quality control of data at the investigational centers by the monitor(s), which involves:
  - ✓ Verifying that the protocol, as well as the current guidelines ICH-GCP, the national regulatory requirements, are adhered to.
  - ✓ Verifying the informed consent and the eligibility of each patient participating in the trial.
  - ✓ Verifying that the eCRF data is consistent and in agreement with the source documents.
  - ✓ Verifying the notification of each SAE.
  - ✓ Verifying the drug traceability (dispatching, storage, and accountability).
  - ✓ Verifying that patients are not already participating in another clinical study making them ineligible for this protocol. The monitor will also verify that patients have not participated in another study within the delay indicated in the non-inclusion criterion #17.
- The quality control of data by a centralized monitoring process. Centralized monitoring is a remote evaluation of data, performed in a timely manner, supported by appropriately qualified and trained persons (e.g., data managers, biostatisticians). Review, that may include statistical analyses, of accumulating data from centralized monitoring can be used to:
  - ✓ Identify missing data, inconsistent data, data outliers, unexpected lack of variability, and protocol deviations.
  - ✓ Examine data trends such as the range, consistency, and variability of data within and across sites.

- ✓ Evaluate for systematic or significant errors in data collection and reporting at a site or across sites, or potential data manipulation or data integrity problems.
- ✓ Analyze site characteristics and performance metrics.
- ✓ Select sites and/or processes for targeted on-site monitoring.

- The audit of participating investigational centers when deemed necessary.

The monitors/CRAs in charge of trial monitoring will be mandated by the sponsor. They must have direct access to all patient data required to perform their duty in accordance with the national regulatory requirements. The monitors/CRAs are bound by professional secrecy under the national regulatory requirements. Written reports must be issued to ensure the traceability of monitoring visits.

To ensure optimal research quality control the investigator will ensure that the monitor/CRA has direct access to all trial patient files.

12.4. Audits and inspections

As part of UNICANCER's audit program, the sponsor may audit some investigational centers. The center and the investigator agree that audits be carried out by sponsor or any person duly authorized during the trial and for at least 15 years after the trial.

The investigational center and the investigator agree to devote the time necessary for the audit procedures, allow the control of the trial documentation, and provide additional information requested by the sponsor.

A Competent Authority may also request a trial inspection (during the trial or after its completion). If a Competent Authority requests an inspection, the investigator must inform the sponsor immediately of this request. The investigator must allow the inspectors direct access to the trial documents and source documents.

The investigational center and the investigator agrees to devote the time necessary for inspections procedures, allow the control of the trial documentation, and provide additional information requested by the inspectors of the concerned Competent Authority.

13. ETHICAL AND REGULATORY CONSIDERATIONS

13.1. General requirements

The clinical study must be conducted in accordance with:

- The principles of ethics as stated in the last version of the Declaration of Helsinki.
- The Good Clinical Practices defined by the International Conference on Harmonization (ICH-E6(R2), 9 November 2016).
- Directive 2001/20/EC of 4 April 2001, of the European Parliament and of the Council on the approximation of the laws, regulations and administrative provisions of the Member States relating to implementation of good clinical practice in the conduct of clinical trials on medicinal products for human use.

- Regulation (EU) 2016/679 on the protection of natural persons with regard to the processing of personal data and on the free movement of such data, and repealing Directive 95/46/EC (General Data Protection Regulation).
- The country specific laws and regulations.

13.2. Patient identification

All patients will receive a unique patient identification number when signing the informed consent form by the patient and before any trial procedure is performed. This number will be used to identify the patient throughout the trial and must be used on all trial documentation related to this patient. The patient identification number must remain constant throughout the trial.

13.3. Patient information and consent

The patient information sheet and informed consent form must be written in accordance with the ICH Harmonized Tripartite Guideline for Good Practical Practice (Appendix 3) and applicable local regulations.

The English version of the patient information sheet (PIS) and informed consent form (ICF) will be considered as the templates that will be translated and adapted to the appropriate national and local regulations. The changes made and the reason for the changes must be provided to UNICANCER. The adapted documents must be validated by UNICANCER before being implemented in the specific country.

Prior to the participation of a patient in the trial, this patient will be informed both verbally and in writing about the objectives of the trial, its methods, anticipated benefits and potential risks and the discomfort to which they may be exposed. All items must be explained by the investigator in a language and in terms that are easy to understand by the patient. The patients must be given enough time to consider their participation and decide whether they wish to participate or not in the trial. Patients will also be informed that their participation is voluntary and that they have the right to withdraw from the trial at any time without giving the reasons and without this impacting their subsequent medical care.

The patient information sheet and the informed consent form must be associated within the same document to ensure that all information regarding the trial is provided to the patient. Patients will confirm their consent in writing prior to starting the trial and before undergoing any trial-related procedure. Two original informed consent forms must be personally dated and signed by the patient and investigator. An original copy will be filed in the Trial Master File (TMF). The other original patient information sheet and the signed informed consent form will be given to the patient.

In the event that the patient decides to withdraw from the trial, the patient is not obliged to give reason(s) for withdrawing. However, the investigator should make a reasonable effort to obtain the reason(s) while fully respecting the patient’s rights.

In conformance with the data protection regulation, the patient may use their right to access to, rectify or oppose the use of their personal data in the research. In these situations, the investigator shall inform the sponsor without delay in order to take the appropriate steps.

If any changes in the written patient information or informed consent form occur during the trial, the investigator will ensures that all patients impacted by the changes and still participating in the trial receive the updated patient information in a timely manner and are asked for written consent for the changes made.

13.4. Insurance compensation

UNICANCER, the sponsor of the trial certifies that it has taken out a civil liability insurance policy covering its civil liability for this clinical trial under its sponsorship. This insurance policy is in accordance with local laws and requirements. The insurance of the sponsor does not exempt the investigator and its team from maintaining their own liability insurance policy.

13.5. Investigator responsibilities

The principal investigator of each investigational center participating in the trial commits to conduct the trial as specified in this protocol and in accordance with the current Declaration of Helsinki (Appendix 2) as well as the current ICH Harmonized Tripartite Guideline for Good Clinical Practice (Appendix 3).

It is the responsibility of the principal investigator to:

- Provide to the sponsor with their curriculum vitae (CV) and those of their collaborators, and evidence that the center will be able to conduct the trial. The CV must be current (no older than 1 year), dated and signed.
- Identify the members of their team who participate in the trial and define each team members role and responsibilities.
- Start recruiting patients only after receiving approval from the sponsor.
- Be available for monitoring visits, audits, and investigator meetings (if applicable).

It is the responsibility of each principal investigator and each investigator team member to:

- Ensure the confidentiality of all data recorded during the trial.
- Collect the informed consent, written, dated, and signed personally by each individual research participant before any specific selection procedure for the trial.
- Regularly complete the eCRF for each patients included in the trial and allow CRAs, mandated by the sponsor, direct access to the source documents in order to validate the data collected in the eCRF.
- Declare to the sponsor as soon as being aware of, any serious adverse event occurring during the trial according to provisions of this protocol.
- Accept regular visits by the CRA(s) and possibly those of auditors mandated by the sponsor or the inspectors of the respective regulatory authorities.
- Date, correct, and sign the corrections made in the eCRF and the requests of the data correction forms (DCF) for each patient included in the trial.

13.6. Federation of the Patient Committees for Clinical Research in Cancerology

This committee reviews trial documents provided to patients in oncology clinical studies, and makes suggestions for improving these, in terms of the quality of information given to patients.

The “Ligue Nationale Contre le Cancer” and the French NCI (INCa) coordinate the French patient committees’ federation.

13.7. Human biological samples collection

Biological studies are necessary to increase the knowledge of diseases, which may allow the development of new and more effective treatments. These studies use human biological samples (blood and tumor samples) than are collected from patients either while they receive medical care (examination, surgery) or specifically for the research purpose.

These biological samples will be prepared, stored, shipped, and used for the purpose of research.

These biological samples are subject to written consent from the patient. This consent is revocable at any time during the trial. Similarly, at any time during the research, the patient has the possibility to request the destruction of their samples.

Concerning **genetic research**, patients must consent to participation in these studies after being informed of the proposed research, irrespective of the type of sample collected (already existing or specifically collected).

Furthermore, it must be noted that the results of biological studies may be published only if all data relative to the patients are made anonymous.

Refer to the Appendix 6 for more detailed information.

13.7.1. *Storage and use of disease assessment samples (blood, biopsy, tumor specimen, etc.)*

During the medical cares that have been realized, biological samples of tissues and/or cells (blood, tumor tissue) will be collected for medical purposes. A part of these samples may be stored and used for scientific researches.

These samples will be used to investigate potential biomarkers predictive of response to DS-8201a in the DAISY study.

The patient will be informed via a patient information sheet and, in the absence of opposition of his part, biological samples for research will be prepared, stored and used for this research.

Refer to the Appendix 6 for more detailed information.

13.7.2. *Collecting additional biological samples for research purpose*

One of the objectives of this research is to study 1) the bystander effect of DS8201 in human samples, 2) the immune effects of DS8201 on metastatic breast cancer tumor cells, and 3) the predictors of primary resistance or outcome to DS-8201a treatment.

To perform this research, additional blood and tumor samples will be collected:

- Mandatory tumor samples to be obtained for all the patients

  - Tumor biopsy at baseline (1 frozen + 2 FFPE)

  - Tumor biopsy at disease progression (Primary resistance PFS < 6 months, Secondary resistance PFS ≥ 6 months) (1 frozen + 2 FFPE)

- Mandatory tumor samples to be obtained for HER2 overexpressed patients, or optional for HER2 low or null patients

  - Tumor biopsy at 6 weeks after treatment initiation (30 patients) (1 frozen+ 1 FFPE)

- Optional tumor samples to be obtained in selected patients and centers

  - Tumor biopsy at 24h (D1) after treatment initiation (10 patients) (1 frozen+ 1 FFPE)

- Mandatory blood samples

  - Whole blood sample at baseline

  - Blood for ctDNA and CTC at baseline, post-C1-C2-C4-C6, and at the end of treatment (whatever the reason)

These biological samples will be prepared, stored and used for the purpose of the research.

In case of leftover samples, they will be used for additional further researches.

Optional samples are subject to a specific additional written consent from the patient. This specific consent for the optional translational research is revocable at any time. In addition, the patient has the right to request the destruction of their samples at any moment.

14. DATA PROCESSING AND CONSERVATION OF DOCUMENTS AND DATA OF THE RESEARCH

14.1. Data processing

14.1.1. *Under the responsibility of the sponsor*

The statistical data will be transferred to the trial statistician for analysis. The trial data remain the property of UNICANCER, the research sponsor.

The software Clinsight® will be used for data entry, management, and archiving of data. The statistical analysis will be performed using the STATA software package.

14.1.2. *In the investigational center, when computerized medical records are used*

If computerized patient records are used in a participating center to process or store trial data, the center must:

- Verify and document that the computer system used to process the data conforms with the requirements concerning data completeness, accuracy, and reliability with respect to expected performances (quality validation).

- Define and follow the standardized procedures related to these systems.
- Ensure that these systems allow modifications of collected data, that each modification is automatically authenticated, and that the data cannot be removed (i.e. any change or modification of the data must be traceable).
- Set up and maintain a security control to prevent unauthorized access to the data.
- Establish and regularly update the list of persons authorized to have access and modify the data.
- Carry out appropriate backups of the data.
- Ensure confidentiality, whenever it is applicable (e.g. during data input).
- Ensure that the individual computerized patient data are processed according to local regulations.

If data are transformed while being processed, it should always be possible to compare them with the original observations/records.

The computerized system used to identify trial patients must not be ambiguous and must allow the identification of all data collected for each patient while maintaining confidentiality in accordance with the national legal requirements.

14.2. Retention of documents by investigator sites

- Approvals from the responsible IEC/IRB for the trial protocol and all amendments.
- Authorizations from respective regulatory authorities for the trial protocol and all amendments.
- All source documents and laboratory records.
- eCRF copies.
- Patients' informed consent forms.
- Investigator master file (IMF) and Investigator master file-pharmacy (IMF-P).
- Any other pertinent trial document.

All trial documents must be kept in a locked and secured place and be considered as confidential.

Data will be archived under the responsibility of the principal investigator of each participating center according to the national regulatory requirements. The trial documents, including a list of the patients' identifications must be archived for a minimum period of 15 years after the end of the trial. UNICANCER will inform the investigational centers when the trial-related records are no longer required.

The investigator must maintain source documents for each trial patient.

All information in case report forms must be traceable and consistent with source documents, which are generally maintained in the patient's file. The source documents should contain all demographic

and medical information, laboratory data, radiology, electrocardiograms, etc., including the original copy of the signed patient information sheet and informed consent form.

The investigator must retain essential documents as described below. The investigator agrees to adhere to the document retention procedures by signing the protocol.

15. DATA OWNERSHIP AND CONFIDENTIALITY

By signing the protocol, the investigator agrees to keep all information provided by UNICANCER strictly confidential and to ensure similar confidentiality from their staff. This obligation does not cover information provided to the patients and information already publically available.

Trial documents provided by UNICANCER (protocols, investigators' brochures, CRFs, and other material) will be stored appropriately to ensure their confidentiality. The information provided by UNICANCER to the physician-investigator may not be disclosed to others without direct written authorization from UNICANCER.

The physician-investigator commits to not publish, spread or use in any manner, directly or indirectly, the scientific and technical information and results related to the trial.

16. PUBLICATION RULES

All information resulting from this trial is considered to be confidential, at least until appropriate analysis and checking has been completed by the sponsor, the principal investigator and the statistician of the trial.

Any publication, abstract or oral presentations including results of the trial must be submitted to the sponsor (UNICANCER) for approval.

Additionally, all communications, manuscripts or oral presentations must include a section mentioning UNICANCER as well as any institution, physician-investigators, collaborative research group, scientific society that has contributed to the trial, including organisations that have provided financial support.

The first author and writer of the main publication will be the principal investigator. The principal investigator may however designate another person to (co-) write the publication.

As for the main publication, authors are listed in the following order:

- The trial coordinator (first or last author).
- The other investigators will appear in the list of co-authors in decreasing order, according to the number of recruited patients regardless of their affiliation to a cooperative group.
- A person representing each cooperating group, if a representative is not listed in the sites with the highest recruitment rates.
- The statistician (the statistician's position is among the first three authors or the last author of the publication).
- A R&D UNICANCER representative.

Similarly, publication of the sub-studies (e.g. biological/ancillary studies) will include persons who have carried out the sub-studies as well as the names of all individuals who have contributed to these sub-studies and a sponsor representative.

It is desirable to include the contributors from weakly recruiting centers who have not been mentioned in the first article in the later publications.

Any conflict regarding publication authorship will initially be submitted to the trial IDMC and then to the CSR (Comité Stratégique Recherche [Strategic Research Committee]) for resolution in case of major disagreement.

UNICANCER will arbitrate and rule any dispute that may arise.

17. REFERENCES

1. Early Breast Cancer Trialists' Collaborative G. Effects of chemotherapy and hormonal therapy for early breast cancer on recurrence and 15-year survival: an overview of the randomised trials. Lancet. 2005;365(9472):1687-717.
2. Andre F, Slimane K, Bachelot T, Dunant A, Namer M, Barrelier A, et al. Breast cancer with synchronous metastases: trends in survival during a 14-year period. J Clin Oncol. 2004;22(16):3302-8.
3. Perou CM, Sorlie T, Eisen MB, van de Rijn M, Jeffrey SS, Rees CA, et al. Molecular portraits of human breast tumours. Nature. 2000;406(6797):747-52.
4. Dawson SJ, Provenzano E, Caldas C. Triple negative breast cancers: clinical and prognostic implications. Eur J Cancer. 2009;45 Suppl 1:27-40.
5. Turner N, Tutt A, Ashworth A. Hallmarks of 'BRCAness' in sporadic cancers. Nat Rev Cancer. 2004;4(10):814-9.
6. Cardoso F, Senkus E, Costa A, Papadopoulos E, Aapro M, Andre F, et al. 4th ESO-ESMO International Consensus Guidelines for Advanced Breast Cancer (ABC 4)dagger. Ann Oncol. 2018;29(8):1634-57.
7. Gelmon K, Chan A, Harbeck N. The role of capecitabine in first-line treatment for patients with metastatic breast cancer. Oncologist. 2006;11 Suppl 1:42-51.
8. Finn RS, Martin M, Rugo HS, Jones S, Im SA, Gelmon K, et al. Palbociclib and Letrozole in Advanced Breast Cancer. N Engl J Med. 2016;375(20):1925-36.
9. Loibl S, Turner NC, Ro J, Cristofanilli M, Iwata H, Im SA, et al. Palbociclib Combined with Fulvestrant in Premenopausal Women with Advanced Breast Cancer and Prior Progression on Endocrine Therapy: PALOMA-3 Results. Oncologist. 2017;22(9):1028-38.
10. Slamon DJ, Clark GM, Wong SG, Levin WJ, Ullrich A, McGuire WL. Human breast cancer: correlation of relapse and survival with amplification of the HER-2/neu oncogene. Science. 1987;235(4785):177-82.
11. Slamon DJ, Leyland-Jones B, Shak S, Fuchs H, Paton V, Bajamonde A, et al. Use of chemotherapy plus a monoclonal antibody against HER2 for metastatic breast cancer that overexpresses HER2. N Engl J Med. 2001;344(11):783-92.
12. Marty M, Cognetti F, Maraninchi D, Snyder R, Mauriac L, Tubiana-Hulin M, et al. Randomized phase II trial of the efficacy and safety of trastuzumab combined with docetaxel in patients with human epidermal growth factor receptor 2-positive metastatic breast cancer administered as first-line treatment: the M77001 study group. J Clin Oncol. 2005;23(19):4265-74.
13. Swain SM, Baselga J, Kim SB, Ro J, Semiglazov V, Campone M, et al. Pertuzumab, trastuzumab, and docetaxel in HER2-positive metastatic breast cancer. N Engl J Med. 2015;372(8):724-34.
14. Swain SM, Kim SB, Cortes J, Ro J, Semiglazov V, Campone M, et al. Pertuzumab, trastuzumab, and docetaxel for HER2-positive metastatic breast cancer (CLEOPATRA study): overall survival results from a randomised, double-blind, placebo-controlled, phase 3 study. Lancet Oncol. 2013;14(6):461-71.
15. Doroshow DB, LoRusso PM. Trastuzumab emtansine: determining its role in management of HER2+ breast cancer. Future Oncol. 2018;14(7):589-602.
16. Verma S, Miles D, Gianni L, Krop IE, Welslau M, Baselga J, et al. Trastuzumab emtansine for HER2-positive advanced breast cancer. N Engl J Med. 2012;367(19):1783-91.
17. Dieras V, Miles D, Verma S, Pegram M, Welslau M, Baselga J, et al. Trastuzumab emtansine versus capecitabine plus lapatinib in patients with previously treated HER2-positive advanced breast cancer (EMILIA): a descriptive analysis of final overall survival results from a randomised, open-label, phase 3 trial. Lancet Oncol. 2017;18(6):732-42.

18. Krop IE, Kim SB, Martin AG, LoRusso PM, Ferrero JM, Badovinac-Crnjevic T, et al. Trastuzumab emtansine versus treatment of physician's choice in patients with previously treated HER2-positive metastatic breast cancer (TH3RESA): final overall survival results from a randomised open-label phase 3 trial. Lancet Oncol. 2017;18(6):743-54.
19. Ogitani Y, Aida T, Hagihara K, Yamaguchi J, Ishii C, Harada N, et al. DS-8201a, A Novel HER2-Targeting ADC with a Novel DNA Topoisomerase I Inhibitor, Demonstrates a Promising Antitumor Efficacy with Differentiation from T-DM1. Clin Cancer Res. 2016;22(20):5097-108.
20. Ogitani Y, Hagihara K, Oitate M, Naito H, Agatsuma T. Bystander killing effect of DS-8201a, a novel anti-human epidermal growth factor receptor 2 antibody-drug conjugate, in tumors with human epidermal growth factor receptor 2 heterogeneity. Cancer Sci. 2016;107(7):1039-46.
21. Tamura K, Tsurutani J, Takahashi S, Iwata H, Krop IE, Redfern C, et al. Sagara Y, Doi T, Park H, Murthy RK, Redman RA, Jikoh T, Lee C, Sugihara M, Shahidi J, Yver A, Modi S. Trastuzumab deruxtecan (DS-8201a) in patients with advanced HER2-positive breast cancer previously treated with trastuzumab emtansine: a dose-expansion, phase 1 study. s. Lancet Oncol 2019; published online April 29. http://dx.doi.org/10.1016/S1470-2045(19)30292-X.
22. Modi S, Tsurutani J, Tamura K, Park H, Sagara Y, Murthy R, et al. Abstract P6-17-02: Trastuzumab deruxtecan (DS-8201a) in subjects with HER2-low expressing breast cancer: Updated results of a large phase 1 study. Cancer Research. 2019;79(4 Supplement):P6-17-02-P6-17-02.
23. Twelves C, Jove M, Gombos A, Awada A. Cytotoxic chemotherapy: Still the mainstay of clinical practice for all subtypes metastatic breast cancer. Crit Rev Oncol Hematol. 2016;100:74-87.
24. Modi S, Saura C, Yamashita T, Park YH, Kim SB, Tamura K, Andre F, Iwata H, Ito Y, Tsurutani J, Sohn J, Denduluri N, Perrin C, Aogi K, Tokunaga E, Im SA, Lee KS, Hurvitz SA, Cortes J, Lee C, Chen S, Zhang L, Shahidi J, Yver A, Krop I; DESTINY-Breast01 Investigators. Trastuzumab Deruxtecan in Previously Treated HER2-Positive Breast Cancer. N Engl J Med. 2020 Feb 13;382(7):610-621. doi: 10.1056/NEJMoa1914510. Epub 2019 Dec 11.25. A'Hern RP. Sample size tables for exact single-stage phase II designs. Stat Med. 2001;20(6):859-66.

18. APPENDICES

- Appendix 1: Performance status evaluation – WHO scale
- Appendix 2: World Medical Association - Declaration of Helsinki
- Appendix 3: ICH Harmonized Tripartite Guideline for Good Clinical Practice (ICH-GCP)
- Appendix 4: A brief summary of tumor classification RECIST v1.1
- Appendix 5: Toxicity criteria (NCI-CTCAE)
- Appendix 6: Translational study
- Appendix 7: Dose modification for DS-8201a
- Appendix 8: Actions required in cases of increases in liver biochemistry and evaluation of Hy's Law

Appendix 1. Performance status evaluation – WHO scale

Performance status<br>ECOG-ZUBROD/ WHO	grade
Fully active, able to carry on all pre-disease performance without Restriction.	0
Restricted in physically strenuous activity but ambulatory and able to carry out work of a light or sedentary nature, e.g. light house work, office work.	1
Ambulatory and capable of all self-care but unable to carry out any work activities; up and about more than 50% of waking hours.	2
Capable of only limited selfcare, confined to bed or chair more than 50% of waking hours.	3
Completely disabled. Cannot carry on any self-care. Totally confined to bed or chair.	4
Dead	5

Developed by the Eastern Cooperative Oncology Group and published by Oken M, Creech R, Tormey D, et al. Toxicity and response criteria of the Eastern Cooperative Oncology Group. Am J Clin Oncol. 1982;5:649-655.

Appendix 2. World Medical Association - Declaration of Helsinki

The current Declaration of Helsinki can be found on the World Medical Association web page via the link provided below:

http://www.wma.net/

Appendix 3. ICH Harmonized Tripartite Guideline for Good Clinical Practice (ICH-GCP)

The current ICH-GCP can be found on the European Medicine Agency web page via the link provided below:

http://www.ema.europa.eu/

Appendix 4. A brief summary of tumor classification RECIST v1.1

(Eur. J. Cancer, 45(2009), 228-247 [47])

Full article available at: http://ctep.cancer.gov/

"New response evaluation criteria in solid tumours: Revised RECIST guideline (version 1.1)" E.A. Eisenhauer, P. Therasse, J. Bogaerts, L.H. Schwartz, D. Sargent, R. Ford, J. Dancey, S. Arbuck, S. Gwyther, M. Mooney, L. Rubinstein, L. Shankar, L. Dodd, R. Kaplan, D. Lacombe, J. Verweij.

Summary:

Measurability of tumor at baseline:

At baseline, tumor lesions/lymph nodes will be categorized measurable or non-measurable as follows:

Measurable

Tumor lesions: Must be accurately measured in a least one dimension (longest diameter in the plane of measurement is to be recorded) with a minimum size of:

- ≥10 mm by CT scan (CT scan slice thickness no greater than 5 mm;
- ≥10 mm calliper measurement by clinical exam;
- 20 mm by chest (=X-ray);

Malignant lymph nodes: To be considered pathologically enlarged and measurable, a lymph node must be ≥ 15 mm in short axis when assessed by CT scan (CT scan slice thickness recommended to be no greater than 5 mm). At baseline and in follow-up, only the short axis will be measured and followed.

Non-measurable

All other lesions, including small lesions (longest diameter <10 mm or pathological lymph nodes with ≥ 10 to <15 mm short axis) as well as truly non-measurable lesions. Lesions considered truly non-measurable include: leptomeningeal disease, ascites, pleural or pericardial effusion, inflammatory breast disease, lymphangitic involvement of skin or lung, abdominal masses/abdominal organomegaly identified by physical exam that is not measurable by reproducible imaging techniques.

Remark:

Bone lesions, cystic lesions, and lesions previously treated with local therapy require special considerations regarding lesion measurability (see below):

Bone lesions:

- Bone scan, PET scan or plain films are not considered adequate imaging techniques to measure bone lesions. However, these techniques can be used to confirm the presence or disappearance of bone lesions.
- Lytic bone lesions or mixed lytic-blastic lesions, with identifiable soft tissue components, that can be evaluated by cross sectional imaging techniques such as CT or MRI can be considered as measurable lesions if the soft tissue component meets the definition of measurability described above.
- Blastic bone lesions are non-measurable.

Target lesions

When more than one measurable lesion is present at baseline all lesions up to a **maximum of five lesions total (and a maximum of two lesions per organ)** representative of all involved organs should be identified as target lesions and will be recorded and measured at baseline. Target lesions should be selected on the basis of their size (lesions with the longest diameter), be representative of all involved organs, but in addition should be those that lend themselves to reproducible repeated measurements. Pathological nodes which are defined as measurable and may be identified as target lesions must meet the criterion of a short axis of ≥ 15 mm by CT scan.

The baseline sum diameters will be used as reference to further characterize any objective tumor regression in the measurable dimension of the disease.

Non-target lesions

All other lesions (or sites of disease) including pathological lymph nodes should be identified as non-target lesions and should also be recorded at baseline. Measurements are not required and these lesions should be followed as ‘present’, ‘absent’, or in rare cases ‘unequivocal progression’ during the trial.

Response criteria:

Target lesions

Complete Response (CR): Disappearance of all target lesions. Any pathological lymph nodes (whether target or non-target) must have reduction in short axis to <10 mm

Warning: lymph nodes identified as target lesions should always have the actual short axis measurement recorded (measured in the same anatomical plane as the baseline examination), even if the nodes regress to below 10 mm on trial. This means that when lymph nodes are included as target lesions, the ‘sum’ of lesions may not be zero even if complete response criteria are met, since

a normal lymph node is defined as having a short axis of <10 mm. In order to qualify for CR, each node must achieve a short axis <10 mm.

Partial Response (PR): At least a 30% decrease in the sum of diameters of target lesions, taking as reference the baseline sum diameters.

Progressive Disease (PD): At least a 20% increase in the sum of diameters of target lesions, taking as reference the smallest sum on study (this includes the baseline sum if that is the smallest on study). In addition to the relative increase of 20%, the sum must also demonstrate an absolute increase of at least 5 mm. (Note: the appearance of one or more new lesions is also considered progression).

Warning: when a progression is recorded with respect to the Nadir but there is a response with respect to baseline, progression must be considered.

Stable Disease (SD): Neither sufficient shrinkage to qualify for PR nor sufficient increase to qualify for PD, taking as reference the smallest sum diameters while on trial.

Non-target lesions

Complete Response (CR): Disappearance of all non-target lesions and normalization of tumor marker level. All lymph nodes must be non-pathological in size (<10 mm short axis).

Non-CR/Non-PD: Persistence of one or more non-target lesion(s) and/or maintenance of tumor marker level above the normal limits.

Progressive Disease (PD): Unequivocal progression (see comments below) of existing non-target lesions. (Note: the appearance of one or more new lesions is also considered progression).

Overall response:

Target lesions	Non-target lesions	New lesions		Overall response
CR	CR	No	=	**CR**
CR	Non CR/Non PD	No	=	**PR**
CR	Not evaluated	No	=	**PR**
PR	Non PD or not all evaluated	No	=	**PR**
SD	Non PD or not all evaluated	No	=	**SD**
Not all evaluated	Non PD	No	=	**Not-evaluable**
PD	No change	Yes or No	=	**PD**
No change	PD	Yes or No	=	**PD**
No change	No change	Yes	=	**PD**

Imaging disease assessments should be repeated at least 4 weeks after assessment of a PR or CR.

Special considerations regarding baseline lesion measurability

Bone lesions:

- Bone scan, PET scan or plain films are not considered adequate imaging techniques to measure bone lesions. However, these techniques can be used to confirm the presence or disappearance of bone lesions.
- Lytic bone lesions or mixed lytic-blastic lesions, with identifiable soft tissue components, that can be evaluated by cross sectional imaging techniques such as CT or MRI can be considered as measurable lesions if the soft tissue component meets the definition of measurability described above.

Cystic lesions:

- Lesions that meet the criteria for radiographically defined simple cysts should not be considered as malignant lesions (neither measurable nor non-measurable) since they are, by definition, simple cysts.
- 'Cystic lesions' thought to represent cystic metastases can be considered as measurable lesions, if they meet the definition of measurability described above. However, if non-cystic lesions are present in the same patient, these are preferred for selection as target lesions.

Lesions with prior local treatment:

- Tumour lesions situated in a previously irradiated area, or in an area subjected to other loco-regional therapy, are usually not considered measurable unless there has been demonstrated progression in the lesion. Trial protocols should detail the conditions under which such lesions would be considered measurable.

Appendix 5. Toxicity criteria (NCI-CTCAE)

In the present trial, adverse events will be recorded according to the

Common Terminology Criteria for Adverse Events (CTCAE) Version 5.0, *(Published: November 27, 2017)*

Toxicity evaluation scale provided separately in attached documents or download it from the NCI website

NATIONAL CANCER INSTITUTE

Cancer Therapy Evaluation Program

http://ctep.cancer.gov/

Appendix 6. Translational study

Translational study

TITLE:

Deciphering mechanisms of action of DS8201

STUDY COORDINATOR:

FABRICE ANDRÉ AND MAGALI LACROIX-TRIKI

CONTACT DETAILS:

FABRICE ANDRÉ

Gustave Roussy
Villejuif-France
Phone: +33 (0)1 42 11 43 71
Email: fabrice.andre@gustaveroussy.fr

MAGALI LACROIX-TRIKI

Gustave Roussy
Villejuif-France
Phone: +33 (0)1 42 11 44 55
Email: Magali.LACROIX-TRIKI@gustaveroussy.fr

STUDY DESCRIPTION/DESIGN:

The translational study group will characterize the mechanism of action of DS-8201a and identify biomarkers that may be predictive of response/resistance to DS-8201a treatments for metastatic breast cancer. They will use tissue and blood samples collected during the DAISY study.

BACKGROUND INFORMATION AND RATIONAL:

HER2, a member of the epidermal growth factor receptor family of transmembrane receptors, is amplified and overexpressed in 25% to 30% of breast cancer tumors and is associated with a poor prognosis (1). Several HER2-targeting therapies such as the antibody-drug conjugate trastuzumab (2) and T-DM1 (3) have been approved worldwide for patients with HER2-positive breast tumors, which are defined as either IHC 3+ or IHC 2+/ISH+. However, there is no HER2-targeting therapy targeting HER2-low expressing tumors such as IHC 1+/2+/ISH-.

DS-8201a is an antibody-drug conjugate (ADC) composed of a recombinant humanized anti-HER2 immunoglobulin G1 monoclonal antibody with the same amino acid sequence of trastuzumab, conjugated to exatecan, a DNA topoisomerase 1 inhibitor, with potential antineoplastic activity (4). *In vitro* and *in vivo* studies demonstrated that DS-8201a binds specifically to the HER2 extracellular domain and does not bind to other HER family proteins. Upon administration of anti-HER2 ADC conjugate DS-8201a, the antibody moiety targets and binds to HER2 on tumor cells. Upon antibody/antigen binding and internalization, the exatecan derivative moiety binds to and inhibits

Top1-DNA complexes, which results in an inhibition of DNA replication, cell cycle arrest and tumor cell apoptosis. In addition, DS-8201a induces antibody-dependent cell-mediated cytotoxicity and causes a bystander killing effect, thereby killing neighboring HER2-expressing tumor cells (5).

However, little is known about the mechanism of action of DS-8201a in breast cancer patients, thus, the translational study will help to describe the bystander effect and immune effects of DS-8201a in human samples. Furthermore, tumor and blood samples from breast cancer patients involved in the Daisy study will be used to investigate biomarkers predictive clinical response to DS-8201a treatment.

OBJECTIVES:

1. Exploration of bystander effect of DS-8201 in human samples.
2. Description of immune effects of DS-8201.
3. Develop predictors of primary resistance or outcome.
4. Identify mechanisms of secondary resistance

SAMPLES REQUIRED:

- Tumor biopsy of metastasis: frozen, and FFPE samples.
- Whole blood sample.
- Blood samples for ctDNA and CTC.

SAMPLE STORAGE AND COLLECTION:

COLLECTION

Mandatory tumor samples to be obtained for all the patients

- Tumor biopsy at baseline (1 frozen + 2 FFPE)
- Tumor biopsy at disease progression: Primary resistance PFS < 6 months, Secondary resistance PFS ≥ 6 months (1 frozen + 2 FFPE)

Mandatory tumor samples to be obtained for HER2 3+ or HER2 2+/ISH+ patients

Tumor biopsy at 6 weeks (D43) after treatment initiation (30 patients) (1 frozen + 1 FFPE)

Optional tumor samples

- to be obtained for HER2 2+/ISH- or HER2 1+ or HER2 0+

  Tumor biopsy at 6 weeks (D1 Cycle 3) **before** 3rd DS-8201a administration (1 frozen + 1 FFPE)
- to be obtained in selected centers

  Tumor biopsy 24h after treatment initiation (D2) (10 patients) (1 frozen + 1 FFPE)

Patients who accept to undergo a biopsy at 24h post-C1 may not be the same than those who accept a biopsy at D43 post-C1.

Mandatory blood samples

- Whole blood sample at baseline
- Blood for ctDNA and CTC at baseline, at D1, post-C1-C2-C4-C6, and at the end of treatment (whatever the reason)

STORAGE

All the samples will be transported and centralized at "Centre des Ressources Biologiques d'UNICANCER" located at the following address:

Centre Léon Berard, 28 rue Laënnec
69373 Lyon cedex 08, Bâtiment CHENEY B Rez de Chaussée
CRB échantillons biologiques- France

PROCESSING OF SAMPLES:

1. Exploration of bystander effect of DS-8201a in human samples

While bystander effect has been described *in vivo* and *in vitro*, there is no evidence for such effect in human. In order to detect bystander effect, we will use samples obtained 24h after the 1st administration of DS-8201a. Patients for this specific aim will be selected in the three cohorts, to ensure a diversity in baseline HER2 expression pattern. Tumor cells will be analyzed by flow cytometry and sorted based on HER2 expression. We will therefore have two sorted cancer cell population, ie HER2-expressing and HER2-non expressing cells. In both cell populations, we will perform dosage of the two component of DS-8201a: trastuzumab and exatecan. The hypothesis is that trastuzumab is not detectable in HER2-negative cells, while exatecan is. At the opposite, we expect both trastuzumab and exatecan to be detected in HER2-positive cells. This experiment will be repeated in ten patients in order to have data about frequency of bystander effect, average concentration of exatecan in HER2-negative cancer cells and some preliminary data of molecular correlation with bystander effect (MDR, Annexin V, etc…). For these ten patients, the data on bystander effect will be correlated with the pattern of HER2 expression at baseline as assessed by IHC and gene-protein assay (see below, Aim 3).

If data on these ten patients generate a hypothesis about molecular mechanisms of bystander effect, more biopsies at D1 will be done. These analyses will be done on FFPE and frozen material collected in three selected centers.

2. Description of immune effects of DS-8201a

Several preclinical studies have suggested that ADC could modulate adaptive immune response through antigen release and immunogenic cell death. Nevertheless, this has never been shown in human. In order to address this question, we will collect biopsies at D43 (+/- 7 days) and will assess local immune response. This will be mandatory in 30 patients with HER2 IHC 3+ or HER2 IHC2+/ISH+ tumor and optional for patients with HER2 IHC2+/ISH- or HER2 IHC1+ or HER2 0+ tumor. Tumor samples will be stained for CD3, CD8, FoxP3, MECA-79, CD11b, and CD14 and will be compared with baseline samples. Immuno-stains will be performed using multiplex protein detection (digital spatial profiling). Stained slides will be digitalized and processed using automated image analysis. In addition, T-cell receptor (TcR) sequencing will be done at baseline and D43 (+/- 7 days).

3. Develop predictors of primary resistance or outcome

The baseline samples obtained in the 162 patients included in the trial will be analyzed by morphology, IHC staining, gene-protein assay, and WES.

- First, slides stained with Hematoxylin-Eosin (HE) will be centrally reviewed by two breast pathologists to assess histopathological criteria (tumor cellularity, histological type, pleomorphism, mitotic activity, necrosis, stromal cells and tumor-infiltrating lymphocytes (TILs)). Slides will also be digitalized and processed using automated image analysis to explore multiple physical random parameters (ie without *a priori*) that could be implemented in a predictor (phenomics).
- Secondly, a two-step analysis will be implemented. During the first step, four sets of biomarkers will be investigated to phenotype the tumors:
  - First, HER2 by ImmunoFluorescence (IF) in order to quantify HER2 expression at the surface of each cell using digital pathology. The HER2 gene-protein assay, that allows determination of HER2 protein (IHC) and *HER2* gene (Dual-ISH) status on the same slide, will be performed to capture intra-tumor heterogeneity for HER2 at the genomic and protein levels. Different types of intra-tumor heterogeneity (regional and interspersed) will be considered. This technique will also allow central review of the HER2 status using the last ASCO/CAP guidelines (6).
  - Second, four markers for clathrin, caveolin, LAMP1 and dynamin 2 in order to explore whether the endosome machinery is predictive for efficacy.
  - Third, biomarkers of drug resistance to exatecan.
  - Fourth, tumor microenvironment with CD3, CD8, FoxP3, CD20, TAM (tumor associated macrophages), MDSC (myeloid-derived suppressor cells), and fibroblasts and endothelial cells biomarkers.
  - For all these markers, highly multiplex protein detection will be performed to allow digital pathology analysis and save the tumor tissue material.

  These analyses will be performed by using highly multiplexed protein detection assays such as the Digital Spatial Profiling/Technology Access Program (nanostring technologies) to allow digital pathology analysis. Image feature extraction and machine learning will be used to predict which pattern of staining is predictive.

  For the second step, one slide will be stained with the biomarkers selected from the first step analysis. New machine learning approaches will be used to integrate all markers in a single multiprotein/gene predictor. Image feature extraction and machine learning will be done by CentraleSupélec Research Center, a center dedicated to applied mathematics.
- Thirdly, whole exome sequencing will be performed on tumor tissue samples to explore genomic alterations that could mediate resistance to DS-8201a.
- Finally, blood sample analysis will include :
  - ctDNA analyses will be performed, using state of the art platforms, with NGS (dedicated breast cancer panel), with the aim of searching for genetic changes driving the early or late resistance. Objectives are:
    - ✓ To define which molecular alterations on plasma are acquired after resistance to DS 8201.
    - ✓ To identify which molecular alterations on plasma are associated with early progression to DS-8201a.
    - ✓ To define whether ctDNA biomarkers could allow early response assessment and/or early diagnosis of progression.

- CTC will be monitored prior to treatment and, if positive, at D1-C2, C3, C5 and C7 to assess for a modification of CTCs during DS-8201a. CTC will be measured using the CellSearch® system in order to:
  - ✓ Describe the variation of CTC levels between the baseline (before the first administration of DS-8201a) and after 3 weeks of treatment
  - ✓ Evaluate the predictive value of the variation of CTC levels on the objective response, the clinical benefit, and survival.
  - ✓ To explore changes over time in tumor cells phenotype induced by ADC

4. Identify mechanisms of secondary resistance

Biopsies obtained after resistance to DS8201 will be analyzed by IHC for proteins that are involved in the mechanism of action and/or are predictive according to the above specific aims 1, 2, 3. In addition, we will perform whole exome sequencing and RNA-seq in resistant samples in order to detect genomic variants that could mediate escape from ADC therapy.

REFERENCES

1. Slamon DJ, Clark GM, Wong SG, Levin WJ, Ullrich A, McGuire WL. Human breast cancer: correlation of relapse and survival with amplification of the HER-2/neu oncogene. Science. 1987;235:177-82.
2. Slamon DJ, Leyland-Jones B, Shak S, Fuchs H, Paton V, Bajamonde A, et al. Use of chemotherapy plus a monoclonal antibody against HER2 for metastatic breast cancer that overexpresses HER2. The New England journal of medicine. 2001;344:783-92.
3. Verma S, Miles D, Gianni L, Krop IE, Welslau M, Baselga J, et al. Trastuzumab emtansine for HER2-positive advanced breast cancer. The New England journal of medicine. 2012;367:1783-91.
4. Royce ME, Hoff PM, Dumas P, et al. Phase I and pharmacokinetic study of exatecan mesylate (DX-8951f): a novel camptothecin analog. J Clin Oncol 2001;19:1493/500.
5. Ogitani Y, Aida T, Hagihara K, Yamaguchi J, Ishii C, Harada N, Soma M2, Okamoto H, Oitate M, Arakawa S, Hirai T, Atsumi R, Nakada T, Hayakawa I, Abe Y, Agatsuma T. DS-8201a, A Novel HER2-Targeting ADC with a Novel DNA Topoisomerase I Inhibitor, Demonstrates a Promising Antitumor Efficacy with Differentiation from T-DM1. Clin Cancer Res. 2016 Oct 15;22(20):5097-5108.
6. Wolff AC, Hammond MEH, Allison KH, Harvey BE, Mangu PB, Bartlett JMS, Bilous M, Ellis IO, Fitzgibbons P, Hanna W, Jenkins RB, Press MF, Spears PA, Vance GH, Viale G, McShane LM, Dowsett M. Human Epidermal Growth Factor Receptor 2 Testing in Breast Cancer: American Society of Clinical Oncology/College of American Pathologists Clinical Practice Guideline Focused Update. J Clin Oncol. 2018 Jul 10;36(20):2105-2122.

Appendix 7. Dose modification for DS-8201a

For studies using NCI-CTCAE version 5.0:

Worst toxicity CTCAE v5.0Grade (unless otherwise specified)	***Dose Management Guidelines for DS-8201a***
No toxicity	*Maintain dose and schedule*
Infusion-Related Reaction	
Grade 1<br>(Mild transient reaction; infusion interruption not indicated; intervention not indicated)	*If infusion related reaction (such as fever and chills, with and without nausea/vomiting, pain, headache, dizziness, dyspnea, hypotension) is observed during administration, the infusion rate should be reduced by 50% and subjects should be closely monitored.<br>If no other reactions appear, the subsequent infusion rate could be resumed at the initial planned rate.*
Grade 2<br>(Therapy or infusion interruption indicated but responds promptly to symptomatic treatment (e.g., antihistamines, nonsteroidal anti-inflammatory drugs (NSAIDs), narcotics, IV fluids); prophylactic medications indicated for ≤ 24 hrs)	*Administration of DS-8201a should be interrupted and symptomatic treatment started (e.g. antihistamines, NSAIDs, narcotics, IV fluids).<br>If the event resolves or improves to grade 1, infusion can be re-started at a 50% reduced infusion rate.<br>Subsequent administrations should be conducted at the reduced rate.*
Grade 3 or 4<br>(Prolonged or life-threatening consequences, urgent intervention indicated)	*Administration of DS-8201a should be discontinued immediately and permanently.<br>Urgent intervention indicated. Antihistamines, steroids, epinephrine, bronchodilators, vasopressors, intravenous fluid therapy, oxygen inhalation etc., should be administered.*
Hematologic Toxicity	
Neutrophil Count Decreased and/or White Blood Cell Count Decreased	
Grade 3	*Delay dose until resolved to ≤ Grade 2, then maintain dose*
Grade 4	*Delay dose until resolved to ≤ Grade 2,<br>Reduce dose 1 level*
Febrile Neutropenia<br>(absolute neutrophil count < 1 x 10^9/L, fever > 38.3°C or a sustained temperature of ≥ 38 °C for more than one hour)	*Delay dose until resolved,<br>Reduce dose by 1 level*
Lymphocyte Count Decreased	
Grade 1 to Grade 3 lymphopenia	*No dose modification*
Grade 4<br>(< 0.2 x 10^9/L)	*Delay dose until resolved to ≤ Grade 2:<br>- If resolved in ≤ 14 days from day of onset, maintain dose*

Worst toxicity CTCAE v5.0Grade (unless otherwise specified)	***Dose Management Guidelines for DS-8201a***
	- If resolved in > 14 days from day of onset, reduce dose 1 level
Anaemia	
Grade 3<br>(Hemoglobin (Hb) <8.0 g/dL); transfusion indicated	*Delay dose until resolved to ≤ Grade 2, then maintain dose*
Grade 4<br>Life threatening consequences; urgent intervention indicated	*Delay dose until resolved to ≤ Grade 2, then reduce dose 1 level*
Platelet Count Decreased	
Grade 3<br>(platelets <50 – 25 x 10^9/L)	*Delay dose until resolved to ≤ Grade 1:<br>- If resolved in ≤ 7 days from day of onset, maintain dose<br>- If resolved in > 7 days from day of onset, reduce dose 1 level*
Grade 4<br>(platelets < 25 x 10^9/L)	*Delay dose until resolved to ≤ Grade 1, then reduce dose 1 level*
Cardiac Toxicity	
Symptomatic congestive heart failure (CHF)	*Discontinue subject from study treatment*
Decrease in Left ventricle ejection fraction (LVEF) 10-20% (absolute value), but LVEF > 45%	*Continue treatment with DS-8201a*
LVEF 40% to ≤ 45% and decrease is < 10% (absolute value) from baseline	*Continue treatment with DS-8201a<br>Repeat LVEF assessment within 3 weeks*
LVEF 40% to ≤ 45% and decrease is ≥ 10-20% (absolute value) from baseline	*Interrupt DS-8201a dosing<br>Repeat LVEF assessment within 3 weeks.<br>If LVEF has not recovered to within 10% (absolute value) from baseline, discontinue subject from study treatment<br>If LVEF recovers to within 10% from baseline, resume study drug treatment*
LVEF < 40% or > 20% (absolute value) drop from baseline	*Interrupt DS-8201a dosing<br>Repeat LVEF assessment within 3 weeks.<br>If LVEF < 40% or > 20% drop from baseline is confirmed, discontinue subject from study treatment*
Electrocardiogram QT prolonged	
Grade 3<br>(Average QTc> 500 ms or >60 ms change from baseline)	*Delay dose until resolved to ≤ Grade 1 (QTc ≤ 480 ms), determine if another medication the subject was taking may be responsible and can be adjusted or if there are any changes in serum electrolytes that can be corrected, then if attributed to DS-8201a, reduce dose 1 level*
Grade 4	*Discontinue subject from study treatment*

Worst toxicity CTCAE v5.0Grade (unless otherwise specified)	***Dose Management Guidelines for DS-8201a***
(Torsade de pointes or polymorphic ventricular tachycardia or signs/symptoms of serious arrhythmia)	

Worst toxicity CTCAE v5.0Grade (unless otherwise specified)	***Dose Management Guidelines for DS-8201a***
Pulmonary Toxicity	*If a subject develops radiographic changes potentially consistent with ILD/pneumonitis or develops an acute onset of new or worsening pulmonary or other related signs/symptoms such as dyspnea, cough or fever, rule out ILD/pneumonitis.*<br>*If the AE is confirmed to have an etiology other than ILD/pneumonitis, follow the management guidance outlined in the "Other Non-Laboratory Adverse Events" dose modification section below.*<br>*If the AE is suspected to be ILD/pneumonitis, treatment with study drug should be interrupted pending further evaluations.*<br>*Evaluations should include:*<br>• *high resolution CT*<br>• *pulmonologist consultation (Infectious Disease consultation as clinically indicated)*<br>• *Blood culture and CBC. Other BLOOD tests could be considered as needed*<br>• *Consider bronchoscopy and bronchoalveolar lavage if clinically indicated and feasible*<br>• *pulmonary function tests and pulse oximetry (SpO2)*<br>• *arterial blood gases if clinically indicated*<br><br>*Other tests could be considered, as needed.*<br>*If the AE is confirmed to be ILD/pneumonitis, follow the management guidance as outlined below.*<br>*All events of ILD regardless of severity or seriousness will be followed until resolution including after drug discontinuation.*
Grade 1	*The administration of DS-8201a must be interrupted for any ILD/pneumonitis events regardless of grade.*<br>• *Monitor and closely follow-up in 2 to 7 days for onset of clinical symptoms and pulse oximetry*<br>• *Consider follow-up imaging in 1-2 weeks (or as clinically indicated).*<br>• *Consider starting systemic steroids (e.g. at least 0.5 mg/kg/day prednisone or equivalent) until improvement, followed by gradual taper over at least 4 weeks.*

Worst toxicity CTCAE v5.0Grade (unless otherwise specified)	***Dose Management Guidelines for DS-8201a***
	• *If worsening of diagnostic observations despite initiation of corticosteroids, then follow Grade 2 guidelines.**<br><br>*For Grade 1 events, DS-8201a can be restarted only if the event is fully resolved to Grade 0, then:*<br>- *If resolved in ≤ 28 days from day of onset, maintain dose*<br>- *If resolved in > 28 days from day of onset, reduce dose 1 level*<br><br>*However, if the event grade 1 ILD/pneumonitis occurs beyond cycle day 22 and has not resolved within 49 days from the last infusion, the drug should be discontinued.*<br><br>** If patient is asymptomatic, then patient should still be considered as Grade 1 even if steroid treatment is given*
Grade 2	*Permanently discontinue subject from study treatment.*<br>• *Promptly start and treat with systemic steroids (e.g., at least 1 mg/kg/day prednisone or equivalent) for at least 14 days or until complete resolution of clinical and chest CT findings , then followed by a gradual taper over at least 4 weeks.*<br>• *Monitor symptoms closely.*<br>• *Re-image as clinically indicated.*<br>• *If worsening or no improvement in clinical or diagnostic observations in 5 days,*<br>• *Consider increasing dose of steroids (e.g., 2 mg/kg/day prednisone or equivalent) and administration may be switched to intravenous (e.g. methylprednisolone).*<br>• *Re-consider additional work-up for alternative etiologies as described above.*<br>• *Escalate care as clinically indicated.*
Grade 3 and 4	*Permanently discontinue subject from study treatment.*<br><br>• *Hospitalization required.*

Worst toxicity CTCAE v5.0Grade (unless otherwise specified)	*Dose Management Guidelines for DS-8201a*
	• *Promptly initiate empiric high-dose methylprednisolone IV treatment (e.g., 500-1000 mg/day for 3 days), followed by at least 1.0 mg/kg/day of prednisone (or equivalent) or at least 14 days or until complete resolution of clinical and chest CT findings, then followed by a gradual taper over at least 4 weeks.*<br>• *Re-image as clinically indicated.*<br>• *If still no improvement within 3 to 5 days,*<br>• *Re-consider additional work-up for alternative etiologies as described above.*<br>*Consider other immuno-suppressants and/or treat per local practice.*
Ocular	
Grade 3	*Delay dose until resolved to ≤ Grade 1:*<br>*If resolved in ≤ 7 days from day of onset, maintain dose*<br>*If resolved in > 7 days from day of onset, reduce dose 1 level*
Grade 4	*Discontinue subject from study treatment*
Blood creatinine increased	
Grade 3 (> 3.0 to 6.0 x upper limit of normal [ULN])	*Delay dose until resolved to ≤ Grade 2 or baseline, then reduce dose 1 level*
Grade 4 (> 6.0 x ULN)	*Discontinue subject from study treatment*
Hepatic Toxicity	
Aspartate aminotransferase (AST) or alanine aminotransferase (ALT) with simultaneous Blood bilirubin increased	
AST/ALT ≥ 3.0 x ULN with simultaneous TBL > 2.0 x ULN	*Delay study medication until drug-induced liver injury can be ruled out.*<br>*If drug-induced liver injury is ruled out, the subject should be treated accordingly, and resumption of study drug may occur after discussion between the Investigator and Sponsor.*<br>*If drug-induced liver injury cannot be ruled out from diagnostic workup, permanently discontinue study treatment.*<br>*Monitor AST/ALT and TBL twice weekly until resolution or return to baseline.*
Aspartate aminotransferase (AST) or alanine aminotransferase (ALT)	
Grade 2 (>3.0 - 5.0 x ULN if baseline was normal; >3.0 - 5.0 x baseline if baseline was abnormal)	*No action for Grade 2 AST/ALT*

Worst toxicity CTCAE v5.0Grade (unless otherwise specified)	***Dose Management Guidelines for DS-8201a***
Grade 3 (>5.0 - 20.0 x ULN if baseline was normal; >5.0 - 20.0 x baseline if baseline was abnormal)<br>*In subjects without liver metastases and subjects with liver metastases and baseline level ≤ 3 x ULN*	*Repeat testing within 3 days. Delay dose until resolved to ≤ Grade 1 if baseline ≤ 3 x ULN, otherwise delay dose until resolved to ≤ baseline, then:*<br>*If resolved in ≤ 7 days from day of onset, maintain dose*<br>*If resolved in > 7 days from day of onset, reduce dose 1 level*
Grade 3: (>8.0 - 20.0 x ULN if baseline was normal; >8.0 - 20.0 x baseline if baseline was abnormal)<br>*In subjects with liver metastases, if the baseline level was > 3 x ULN*	*Repeat testing within 3 days. Delay dose until resolved to ≤ baseline level, then:*<br>*If resolved in ≤ 7 days from day of onset, maintain dose*<br>*If resolved in > 7 days from day of onset, reduce dose 1 level*
Grade 4 (>20.0 x ULN if baseline was normal; >20.0 x baseline if baseline was abnormal)	*Discontinue subject from study treatment*
Blood Bilirubin increased	
Grade 2 (>1.5 - 3.0 x ULN if baseline was normal; >1.5 - 3.0 x baseline if baseline was abnormal)	*If no documented Gilbert's syndrome or liver metastases at baseline, delay dose until resolved to ≤ Grade 1:*<br>- *If resolved in ≤ 7 days from day of onset, maintain dose*<br>- *If resolved in > 7 days from day of onset, reduce dose 1 level*<br><br>*If documented Gilbert's syndrome or liver metastases at baseline, continue study treatment*
Grade 3 (>3.0 - 10.0 x ULN if baseline was normal; >3.0 - 10.0 x baseline if baseline was abnormal)	*If no documented Gilbert's syndrome or liver metastases at baseline, repeat testing within 3 days. Delay dose until resolved to ≤ Grade 1:*<br>- *If resolved in ≤ 7 days from day of onset, reduce dose 1 level*<br>- *If resolved in > 7 days from day of onset, discontinue DS-8201a*<br><br>*If documented Gilbert's syndrome or liver metastases at baseline, repeat testing within 3 days. Delay dose until resolved to ≤ Grade 2:*<br>- *If resolved in ≤ 7 days from day of onset, reduce dose 1 level*<br>- *If resolved in > 7 days from day of onset, discontinue DS-8201a*
Grade 4 (>10.0 x ULN if baseline was normal; >10.0 x baseline if baseline was abnormal)	*Discontinue subject from study treatment*
Blood Alkaline Phosphatase Increased	

Worst toxicity CTCAE v5.0Grade (unless otherwise specified)	***Dose Management Guidelines for DS-8201a***
Grade 3 (>5.0 - 20.0 x ULN if baseline was normal; >5.0 - 20.0 x baseline if baseline was abnormal)<br>or<br>Grade 4 (>20.0 x ULN if baseline was normal; >20.0 x baseline if baseline was abnormal)	*No modification unless determined by the Investigator to be clinically significant or life-threatening.*
Gastrointestinal	
Nausea	
Grade 3	*Delay dose until resolved to ≤ Grade 1<br>If resolved in ≤ 7 days from day of onset, maintain dose<br>If resolved in > 7 days from day of onset, reduce dose 1 level*
Diarrhoea/Colitis	
Grade 3	*Delay dose until resolved to ≤ Grade 1<br>If resolved in ≤ 3 days from day of onset, maintain dose<br>If resolved in > 3 days from day of onset, reduce dose 1 level*
Grade 4	*Discontinue subject from study treatment*
Other Laboratory Adverse Events	
Grade 3	*Delay dose until resolved to ≤ Grade 1 or baseline level:<br>If resolved in ≤ 7 days from day of onset, maintain dose<br>If resolved in > 7 days from day of onset, reduce dose 1 level*
Grade 4	*Discontinue subject from study treatment*
Other Non-Laboratory Adverse Events	
Grade 3	*Delay dose until resolved to ≤ Grade 1 or baseline:<br>If resolved in ≤ 7 days from day of onset, maintain dose<br>If resolved in > 7 days from day of onset, reduce dose 1 level*
Grade 4	*Discontinue subject from study treatment*

All dose modifications should be based on the worst preceding toxicity.
CTCAE: Common Terminology Criteria for Adverse Events.

Appendix 8. Actions required in cases of increases in liver biochemistry and evaluation of Hy's Law

1. Introduction

This appendix describes the process to be followed in order to identify and appropriately report cases of Hy's Law. It is not intended to be a comprehensive guide to the management of elevated liver biochemistries.

During the course of the study the investigator will remain vigilant for increases in liver biochemistry. The investigator is responsible for determining whether a patient meets potential Hy's Law criteria at any point during the study. Hy's law criteria are met if there is no alternative explanation for the elevations in liver biochemistry other than drug induced liver injury caused by the DS-8201a.

The investigator is responsible for recording data pertaining to potential Hy's law/Hy's law cases and for reporting Adverse Events (AE) and Serious Adverse Events (SAE).

2. Definitions

2.1. Potential Hy's Law

Aspartate aminotransferase (AST) or alanine aminotransferase (ALT) ≥3 × ULN **and** total bilirubin >2 × ULN occurring either at different time points or simultaneously during the study following the initiation of study medication irrespective of an increase in alkaline phosphatase (ALP).

2.2. Hy's Law

AST or ALT ≥3 x ULN **together with** bilirubin > 2 x ULN, where no other reason, other than the DS-8201a, can be found to explain the combination of increases, *e.g.* elevated ALP indicating cholestasis, viral hepatitis, or another drug.

For potential Hy's law and Hy's law the elevation in transaminases must precede or be coincident with (*i.e.* on the same day) the elevation in bilirubin, but there is no specified period within which the elevations in transaminases and bilirubin must occur.

3. Identification of potential Hy's Law cases

In order to identify cases of potential Hy's law it is important to perform a comprehensive review of laboratory data for any patient who meets any of the following identification criteria in isolation or in combination:

- ALT ≥3 x ULN
- AST ≥3 x ULN
- bilirubin >2 x ULN

The investigator will without delay review each new laboratory report and if the identification criteria are met will:

- Determine whether the patient meets potential Hy's law criteria (see Section 2.1. for definition) by reviewing laboratory reports from all previous visits.
- Promptly enter the laboratory data into the eCRF.

4. Follow-up

4.1. Potential Hy's Law criteria not met

If the patient does not meet potential Hy's law criteria the investigator will perform follow-up on subsequent laboratory results according to the guidance provided in the clinical study protocol.

4.2. Potential Hy's Law criteria met

If the patient does meet potential Hy's law criteria the investigator will:

- Monitor the patient until liver biochemistry parameters and appropriate clinical symptoms and signs return to normal or baseline levels, or as long as medically indicated.
- Investigate the etiology of the event and perform diagnostic investigations.
- If at any time the potential Hy's law case meets serious criteria, report it as an SAE in the eCRF.

5. Review and assessment of potential Hy's Law cases

The instructions in this section should be followed for all cases where potential Hy's law criteria are met.

No later than 3 weeks after the biochemistry abnormality was initially detected, the investigator contacts the sponsor in order to review available data and agree on whether there is an alternative explanation for meeting potential Hy's law criteria other than drug-induced liver injury caused by DS-8201a.

According to the outcome of the review and assessment, the investigator will follow the instructions below.

- If there is an agreed alternative explanation for the ALT or AST and bilirubin elevations, a determination of whether the alternative explanation is an AE will be made and subsequently whether the AE meets the criteria for a SAE:
  - If the alternative explanation is **not** an AE, record the alternative explanation on the appropriate eCRF.
  - If the alternative explanation is an AE/SAE, record the AE/SAE in the eCRF accordingly and follow the standard processes.
- If it is agreed that there is **no** explanation that would explain the ALT or AST and bilirubin elevations other than DS-8201a:
  - Report an SAE (report term 'Hy's Law') according to standard processes:
    - The 'Medically Important' serious criterion should be used if no other serious criteria apply.

- ❖ As there is no alternative explanation for the Hy's law case, a causality assessment of 'related' should be assigned.

- If there is an unavoidable delay of over 3 weeks to obtain the information necessary to assess whether or not the case meets the criteria for Hy's law, then it is assumed that there is no alternative explanation until such time as an informed decision can be made:
  - ➢ Report an SAE (report term 'Potential Hy's Law') applying serious criteria and causality assessment as per above.
  - ➢ Continue follow-up and review according to agreed plan. Once the necessary supplementary information is obtained, repeat the review and assessment to determine whether Hy's law criteria are met. Update the SAE report according to the outcome of the review amending the reported term if an alternative explanation for the liver biochemistry elevations is determined.

Appendix 9. Instructions related to severe acute respiratory syndrome coronavirus 2 (SARS-COV-2)

Prior and Concomitant Medications

Concomitant treatment with chloroquine or hydroxychloroquine is not allowed during the study treatment. If treatment with chloroquine or hydroxychloroquine treatment is absolutely required for SARS-CoV-2 (ie COVID-19), study treatment must be interrupted. If chloroquine or hydroxychloroquine is administered, then a wash-out period of no less than 14 days is required before restarting study treatment.

SARS-CoV-2 Assessment(s)

All confirmed or suspected SARS-CoV-2 infection events must be recorded in the eCRF. If a subject presents to the clinic with symptoms suggestive of SARS-CoV-2, but the real-time RT-PCR test is not available at the site, a [nasopharyngeal swab/saliva] sample kit will be provided for sample collection to be tested at a central laboratory. The results will be provided to the site from the central laboratory.

Dose modification criteria for suspected or confirmed SARS-CoV-2

All confirmed or suspected SARS-CoV-2 infection events must be recorded in the eCRF. Dose modifications will be based on the worst CTCAE grade. All interruptions or modifications must be recorded on the AE and drug administration eCRFs. **Please use CTCAE v5.0 general grading criteria to evaluate COVID-19.** All dose modifications (discontinuation, interruptions or reductions) must be recorded on the AE and drug administration eCRFs.

Dose modification criteria

If SARS-CoV-2 infection is suspected, interrupt DS 8201a and rule out SARS-CoV-2 per local guidance.

- If SARS-CoV-2 is ruled out, follow dose modification and management guidelines as outlined in the CE protocol template.
- If SARS-CoV-2 is confirmed or diagnosis is suspected after evaluation, follow dose modification as outlined below and manage SARS-CoV-2 per local guidance until recovery of SARS-CoV-2. SARS-CoV-2 recovery is defined as no signs/symptoms SARS-CoV-2, at least 1 negative RT-PCR test result*,and nearly or completely resolved chest CT findings. Then follow below dose modifications:

SARS-CoV-2 Dose Modification Criteria

SARS-CoV-2 Worst Toxicity NCI-CTCAE Version 5.0 Grade (unless otherwise specified)	Schedule Modification for DS8201a
Grade 1	Resume study drug at the same dose[a]

SARS-CoV-2 Worst Toxicity NCI-CTCAE Version 5.0 Grade (unless otherwise specified)	Schedule Modification for DS8201a
Grade 2	Resume study drug at the same dose if chest CT findings are completely resolved[a]<br>Reduce by 1 dose level if chest CT findings are nearly resolved
Grade 3	Reduce by 1 dose level if chest CT findings are completely resolved<br>Discontinue study drug if chest CT findings are **not** completely resolved
Grade 4	Discontinue study drug

SARS-CoV-2 = severe acute respiratory syndrome coronavirus 2 (SARS-CoV-2); CT = computed tomography
[a] Closely monitor signs/symptoms after resuming study treatment, initially with a phone call every 3 days for the first week, and then with a weekly phone call thereafter, for a total of 6 weeks.

- In addition to the recommendations outlined in the table above, Investigators may consider dose modifications of the study drug according to the subject's condition and after discussion with the study Medical Monitor or designee.
- If an event is suspected to be drug related ILD/pneumonitis, manage per protocol ILD/pneumonitis management guideline.

* If PCR testing is not available, the subject must not have any sign/symptoms for at least 2 weeks, in addition to meeting the requirement for chest CT imaging.
